# Analysis of Solid‐State Luminescence Emission Amplification at Substituted Anthracenes by Host–Guest Complex Formation

**DOI:** 10.1002/chem.202003017

**Published:** 2020-10-27

**Authors:** Timo Schillmöller, Paul Niklas Ruth, Regine Herbst‐Irmer, Dietmar Stalke

**Affiliations:** ^1^ Institut für Anorganische Chemie Georg-August-Universität Göttingen Tammannstraße 4 37077 Göttingen Germany

**Keywords:** aggregation-induced emission, fluorescence, host–guest systems, main group elements, solid-state fluorescence

## Abstract

Small robust organic molecules showing solid‐state luminescence are promising candidates for optoelectronic materials. Herein, we investigate a series of diphenylphosphanyl anthracenes [9‐PPh_2_‐10‐R‐(C_14_H_8_)] and their sulfur oxidised analogues. The oxidation causes drastic changes in the molecular structure as the new orientation of the bulky (S)PPh_2_ substituent induces a strong butterfly bent structure of the anthracene core, which triggers a strong bathochromic shift resulting in a green solid‐state fluorescence. As the emission properties change only slightly upon aggregation the origin of the emission is attributed to a typical monomer fluorescence. The host–guest complexes of [9‐(S)PPh_2_‐10‐Ethyl‐(C_14_H_8_)] with four basic arenes reveal an emission enhancement up to five‐times higher quantum yields compared to the pure host. Less interchromophoric interactions and a restriction of intramolecular motion within the host molecules due to fixation by weak C−H⋅⋅⋅π interactions with the co‐crystallised arene are responsible for that emission enhancement.

## Graphical abstract

**A series of** (diphenylphosphanyl)anthracenes and their sulfur‐oxidation products were synthesised and their photophysical properties investigated. Intra‐ and intermolecular features were quantified and correlated to their solid‐state emission properties. Co‐crystallisation of small aromatic molecules lead to in amplification of the solid‐state emission.

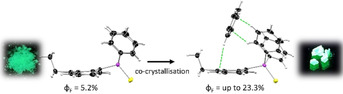

## Introduction

Organic luminescent solid‐state materials have spawned great interest in fundamental research and various applications over the last two decades. The great potential in practical utilization of those materials in OLEDs,^[1]^ OFETs,^[2]^ sensors,^[3]^ and lasers^[4]^ have been documented in a vast number of previous publications. Nevertheless, most applications in the molecular regime are still dominated by d‐ or f‐block organometallic coordination complexes due to their excellent optoelectronic properties.[^5^, ^6^] However, organic luminescent materials without any metals are beneficial in many respects compared to commonly used materials: simple synthesis, lower costs, and superior environmental compatibility.^[7]^ Even if the photophysical properties of common organic chromophores, such as polyaromatic hydrocarbons, are investigated widely and well understood in solution the material's profile often changes drastically upon aggregation in the solid‐state.^[8]^ Frequently the excellent luminescence properties in solution are completely vanished in the solid‐state and fluorescence is nearly quenched upon aggregation (*Aggregation‐caused‐quenching*, ACQ).[^9^, ^10^, ^11^, ^12^, ^13^] Furthermore, the electronic structure of the molecule and the inter‐ and intramolecular interaction pathways in the solid‐state can cause a variety of non‐radiative decays and fluorescence quenching (Förster‐,^[14]^ Dexter‐,^[15]^ excimer‐ or exciplex‐quenching^[12]^). Various strategies have been established to overcome the quenching in the solid‐state and to obtain efficient luminescent solid‐state materials. When these compounds are not emissive in solution and only emit upon aggregation the phenomenon is called *Aggregation‐Induced‐Emission* (AIE), which has been first observed by Jelley^[16]^ and Scheibe^[17]^ in the 1930s^[18]^ and became more popular in the 2000s by the work of Tang.[^9^, ^10^, ^11^, ^19^] AIE‐luminogens are usually non‐emissive in solution due to non‐radiative relaxation pathways like rotation or vibration. In the solid‐state these channels are blocked upon aggregation and excitation results in an efficient radiative decay.^[20]^ Several molecular building blocks with well‐known rotations like tetraphenylethylene (TPE) show AIE‐behaviour and therefore a whole cornucopia of luminescent compounds of this type are known and investigated.^[21]^

Covalent routes aim to the introduction of bulky substituents to the fluorophore to reduce interchromophoric interactions and obtain efficient monomer‐like emission not only in solution but also in the aggregated state.^[22]^ Introduction of donor‐acceptor moieties facilitates tuneability of the absorption and emission properties via varying the HOMO–LUMO gap of the fluorophores.

In addition to the covalent strategies, non‐covalent routes are also used for optimizing and tuning emission properties.^[23]^ Weak non‐covalent interactions like hydrogen‐bonds, π‐π and C−H⋅⋅⋅π interactions are known to modulate photophysical properties in the solid‐state.^[24]^ Through rational molecular design these interactions are used to obtain distinct molecular assemblies resulting in efficient excimer or exciplex emission instead of fluorescence quenching.^[25]^ Excimer and exciplex type interaction usually follows a bathochromic shift of the emission and extends fluorescence’ lifetimes.^[26]^ Exciplex emission can also be obtained by host–guest complexes or supramolecular assemblies.^[27]^ Face‐to‐face interactions of the host molecule and suitable guests can tune the emission wavelengths and effectiveness via a Charge‐Transfer (CT) or exciplex mechanism.^[28]^ Some of these systems show interesting vapochromic behaviour and are also potential candidates for chemical sensing of volatile, organic compounds.[^6^, ^29^] One of the first sensors for toluene based on a host–guest system we reported in 2003.^[30]^ The unusual photophysical properties of a disubstituted diphenyl(thiophosphoranyl) anthracene revealed a reversible intense green emission upon co‐crystallisation with toluene and a nearly complete fluorescence quenching when the toluene was removed under reduced pressure. The fluorescence could be restored with the addition of a small amount of toluene. As other aromatic compounds like benzene originally were not able to recover the fluorescence the compound was considered as a potential chemosensor for detecting toluene and the structural properties were studied intensely.[^31^, ^32^] Later, further dipheny(thiophosphoranyl) substituted polyaromatic hydrocarbons have been reported and especially the anthracenyl derivative revealed interesting structural features like a strong bending of the anthracene core, which was assumed to influence the spectroscopic properties.^[33]^

For a further investigation we synthesized and characterised a class of thiophosphoranyl anthracenes in the present study to investigate the structure‐property‐relationship especially in terms of solid‐state luminescence.

## Results and Discussion

### Synthesis

**Scheme 1:**
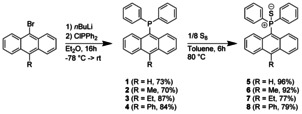
Syntheses of diphenylphosphanyl anthracenes [9‐PPh_2_‐10‐R‐(C_14_H_8_)] (**1**–**4**) with various substituents in 10‐position and their corresponding sulfur oxidised products [9‐(S)PPh_2_‐10‐R‐(C_14_H_8_)] (**5**–**8**).

Four diphenylphosphanyl anthracenes [9‐PPh_2_‐10‐R‐(C_14_H_8_)] (**1**–**4**, R=H, Me, Et, Ph) with various substituents in the 10‐position and their sulfur‐oxidised products (**5**–**8**) were synthesized from the corresponding bromoanthracenes adapting literature procedures (Scheme 1).[^33^, ^34^] Lithium‐halogen exchange with *n*BuLi at low temperatures and subsequent addition of chlorodiphenylphosphane affords the diphenylphosphanyl anthracenes in good yields. **1**–**4** reveal respectable stability against air and moisture as solids and get oxidised only slowly in aerated solutions. The ^31^P‐NMR chemical shifts are in a typical region of about −25 ppm. The P(iii) atom can easily be oxidised to P(v) by elemental sulfur in toluene at 80 °C. The ^31^P‐NMR signals of the resulting thiophosphoranyl anthracenes are shifted downfield to about +34 ppm, which is slightly different compared to other diphenyl thiophosphoranyl substituted arenes.^[33]^ They usually exhibit chemical shifts in a narrow range of 42–43 ppm (Table S1).

### Structural Properties

**Figure 1:**
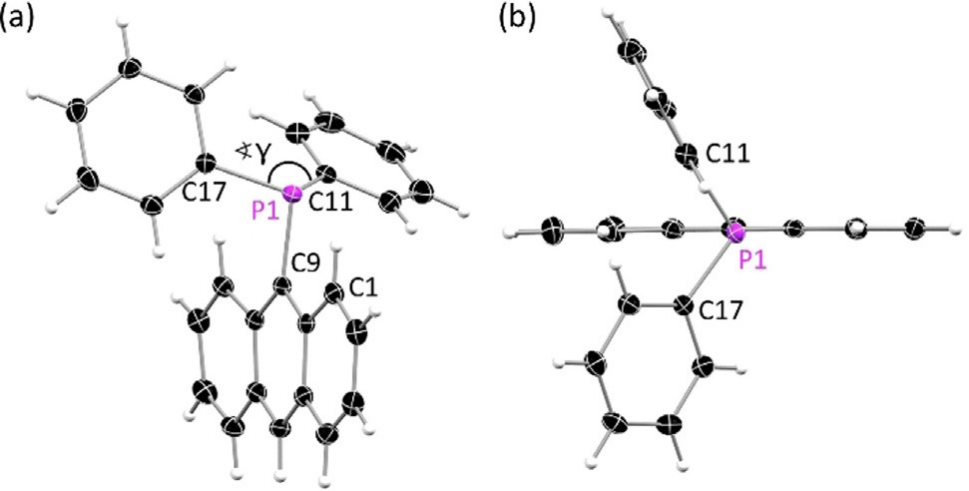
(a) Molecular structure of 9‐diphenylphosphanyl anthracene [9‐PPh_2_(C_14_H_9_)] (**1**). (b) View along the P1‐C9‐C10 axis reveals the orientation of the phenyl substituents above and below the anthracene plane and the planarity of the anthracene moiety. Anisotropic displacement parameters are depicted at the 50 % probability level. Only one molecule of the asymmetric unit is shown. Crystallographic details are given in Table S2.

Crystallisations of **1**–**4** from dichloromethane (DCM) or toluene solutions afford single crystals suitable for structure determination. **1**–**3** crystallise in the triclinic space group *P* $\bar{1}$ , while **4** adopts a monoclinic crystal system in the space group *C*2/*c*. The asymmetric units of **1** and **2** consist of two slightly different molecules, while in **3** and **4** only one molecule is present. **4** co‐crystallises with one toluene molecule. The anthracene moieties are nearly planar, and the phosphorus atom adopts a pyramidal geometry with one phenyl group above and one below the anthracene moiety (Figure 1, Table 1). Therefore, the lone pair is located nearly in the anthracene plane. The phenyl group in the 10‐position of **4** reveals a rather orthogonal orientation towards the anthracene plane, with an intersection angle of the two planes of 66.78(6)° (Figure S1).

**Table 1:** Structural properties of diphenylphosphanyl anthracenes **1**–**4**, their sulfur‐oxidised products **5**–**8** and co‐crystals **7 a**–**7 d**.

	SPCC [°]	γ [°]	α [°]
[9‐PPh_2_(C_14_H_9_)] (**1**)	–	106.38(8) 104.92(7)	6.82(15) 1.58(16)
[9‐PPh_2_‐10‐Me‐(C_14_H_8_)] (**2**)	–	109.21(6) 105.27(7)	3.07(16) 1.95(13)
[9‐PPh_2_‐10‐Et‐(C_14_H_8_)] (**3**)	–	106.62(7)	6.58(11)
[9‐PPh_2_‐10‐Ph‐(C_14_H_8_)] (**4**)	–	105.77(9)	7.50(18)
[9‐(S)PPh_2_(C_14_H_9_)] (**5**)	80.84(10) 89.91(10)	101.65(6) 100.95(4)	11.67(13) 16.86(11)
[9‐(S)PPh_2_‐10‐Me‐(C_14_H_8_)] (**6**)	85.54(11) 85.72(11)	98.91(6) 98.92(6)	16.95(10) 17.36(10)
[9‐(S)PPh_2_‐10‐Et‐(C_14_H_8_)] (**7**)	87.89(13) 89.01(12)	98.50(7) 99.29(7)	14.83(17) 15.62(12)
[9‐(S)PPh_2_‐10‐Et‐(C_14_H_8_)] (**7 a**)	79.43(13)	99.99(8)	9.42(11)
[9‐(S)PPh_2_‐10‐Et‐(C_14_H_8_)] (**7 b**)	78.54(15)	100.39(9)	7.78(11)
[9‐(S)PPh_2_‐10‐Et‐(C_14_H_8_)] (**7 c**)	80.06(10)	100.37(6)	8.05(8)
[9‐(S)PPh_2_‐10‐Et‐(C_14_H_8_)] (**7 d**)	84.19(9)	98.47(5)	16.60(9)
[9‐(S)PPh_2_‐10‐Ph‐(C_14_H_8_)] (**8**)	86.1(2)	98.24(13)	10.7(2)

**Figure 2:**
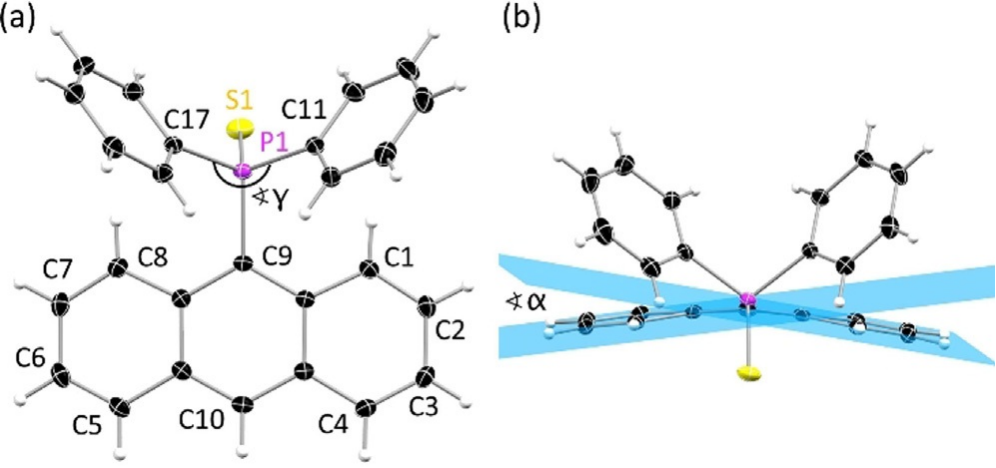
(a) Molecular structure of the oxidized [9‐(S)PPh_2_(C_14_H_9_)] (**5**) showing the new orientation of the substituent with a decreased C11‐P‐C17 angle γ. (b) The new orientation and the increased steric strain leads to a butterfly deformation of the anthracene plane which is characterized by the folding angle α. Anisotropic displacement parameters are depicted at the 50 % probability level. Only one molecule of the asymmetric unit is shown. Crystallographic details are given in Table S3.

The P−C bond lengths of 1.82 Å (P−*C_Ph_*) and 1.86 Å (P−C9) are in a typical range for tertiary aromatic phosphanes.^[35]^ The overall crystal packing revealed only weak interactions between the fluorophores. The anthracene planes of neighbouring molecules show only little overlap and no significant π–π interactions as the phenyl groups above and below the anthracene shield the aromatic plane. The solid‐state structure is mainly built up by weak C−H⋅⋅⋅π interactions (Figure S3–S6). The crystal packings of **1**–**4** are shown in Figure S7 and S8. Upon oxidation of the phosphorus atom with sulfur the molecular structural parameters and the crystal packing changes dramatically (Figure S9–S12). Single crystals for structure determination were obtained by recrystallisation from toluene (**5**, **6**), ethyl acetate (EtOAc) (**7**) or cyclohexane (**8**). [9‐(S)PPh_2_(C_14_H_9_) (**5**) and [9‐(S)PPh_2_‐10‐Et‐(C_14_H_8_)] (**7**) crystallise in the triclinic space group *P* $\bar{1}$ with two slightly different molecules in the asymmetric unit. **6** crystallises in the monoclinic space group *P*2_1_/*c* with also two molecules present in the asymmetric unit, while **8** reveals only one molecule in the asymmetric unit and adopts an orthorhombic crystal system in the non‐centrosymmetric space group *P*2_1_2_1_2_1_. As expected, the P−C bond lengths are slightly shorter compared to unoxidised **1**–**4** and in a range of 1.82 Å to 1.84 Å, but this shortening is more pronounced for the P−C9 bond. Furthermore, the oxidation induces a new orientation of the substituent relative to the anthracene moiety in all four compounds. The two phenyl groups are now both located above the anthracene plane, while the sulfur is underneath (Figure 2). The sulfur‐phosphorus bond is oriented nearly orthogonal to the anthracene plane with S1‐P1‐C9‐C8A torsion angles ranging from 78.54(15)°–89.91(10)° (Table 1). The sulfur atom increases the steric demand compared to the lone pair and as a consequence the C11‐P1‐C17 angle (γ) decreases. (Figure 2). Moreover, a strong distortion of the anthracene plane is observed resulting in an along C9⋅⋅⋅C10 bent butterfly orientation with the wings towards the sulfur atom, which has been observed for **5** in previous studies.[^31^, ^32^, ^33^] The grade of that butterfly arrangement is quantified by the folding angle *α*, which is the intersecting angle between two planes through the outer four C‐atoms (C1 to C4 and C5 to C8) (Figure 2 a). In order to investigate, whether the different orientation of the phenyl groups in **1** and **5** is a crystal packing effect or inherent to the structures themselves, theoretical geometry optimisations were carried out at the D3‐B3LYP//def2‐TZVP level. The lowest energy structure of **1** shows the expected orientation of the phenyl groups above and below the anthracene, with a calculated γ of 104.92° and near planar arrangement of the anthracene with a negligible folding angle *α* of only 1.58° In contrast the energetic minimum of **5** shows an orientation of the two phenyl groups at the same side of the anthracene plane. The angle γ is reduced to only 96.97° and the anthracene plane shows a considerable distortion with the angle *α* being 11.35°. Depictions of the optimised structures, as well as additional information can be found in the supporting information. From these calculations we conclude that the solid‐state structures do indeed represent energetic minima in the gas phase on their own right.

**Figure 3:**
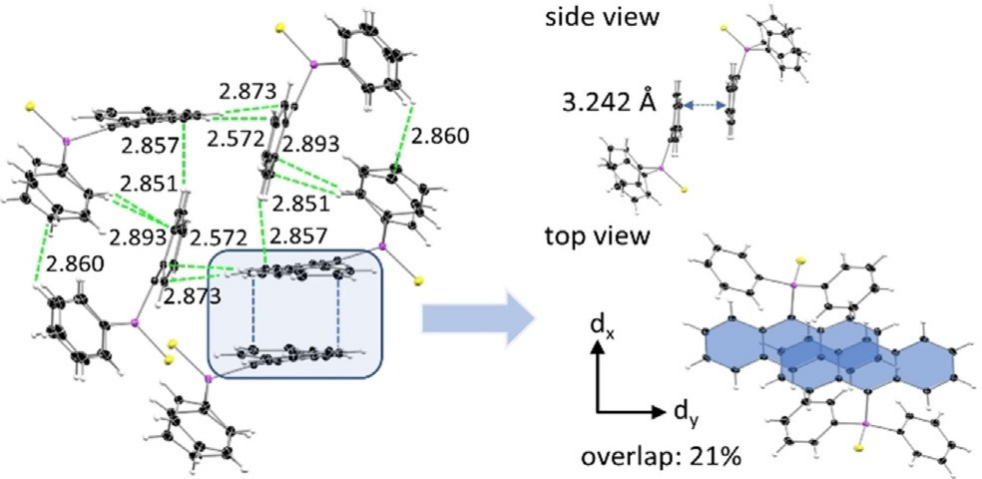
Excerpt of the crystal packing of [9‐(S)PPh_2_(C_14_H_9_)] (**5**) with various C−H⋅⋅⋅π‐interactions in the range of 2.57–2.89 Å (green) and face‐to‐face interactions of two slightly overlapped anthracene moieties (blue).

**Figure 4:**
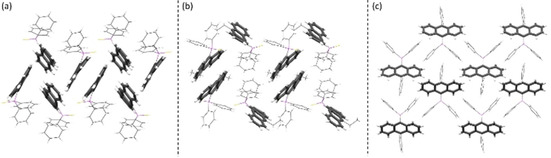
Crystal packing motifs observed in the solid‐state structures of the thiophosphoranyl anthracenes. (a) Dimeric herringbone packing with edge‐to‐face interactions of neighbouring anthracenes in **5**. (b) Isolated dimeric stacking as seen in **6** and **7**. (c) In **8** only weak intermolecular interactions are observed due to a second bulky substituent in 10‐position.

In addition to the molecular changes the oxidation of the P(iii) to a P(v) atom leads to changes in the crystal packing. While the phosphanyl anthracenes **1**–**4** reveal only weak C−H⋅⋅⋅π‐interactions and no close contacts of the anthracene moieties, the thiophosphoranyl derivatives show a variety of interactions, which are also controlled by the substituent in the 10‐position. The monosubstituted [9‐(S)PPh_2_‐(C_14_H_9_)] (**5**) displays strong edge‐to‐face interactions between two adjacent anthracenes resulting in a dimeric herringbone packing motif (Figure 3). Further interactions are present between the phenyl H‐atoms and the anthracene π‐system and between two phenyl groups. C−H⋅⋅⋅π‐interactions are in a range from 2.758–2.893 Å. The face‐to‐face interactions between two anthracenes are in an antiparallel fashion and can be considered as rather weak, with an estimated overlap^[36]^ of 21 % and a π–π distance of 3.242 Å. The methyl‐ (**6**) and ethyl substituted (**7**) derivatives exhibit similar packing motifs. As expected, introduction of the substituents in the 10‐position inhibit strong edge‐to‐face interactions of the anthracene moieties. Instead, the face‐to‐face interactions are more pronounced with an increased overlap of the aromatic planes. The anthracene site with the alkyl groups attached holds a higher electron density due to the electron‐donating character of the alkyl substituent. Therefore, a larger antiparallel overlap with the more electron poor site of the adjacent anthracene is possible. The overlap increases from 21 % (**5**) to 33 % (**6**) and 43 % (**7**), which goes along with a constant decrease of the offset along the short molecular anthracene axes (d_x_) (Figure 3, Table 2). The crystal packing layers (Figure 4, S14–S15). Furthermore, C−H⋅⋅⋅π‐ interactions with distances from 2.740 to 2.897 Å are present (Figure S17–S18). With the introduction of the phenyl substituent in 10‐position a third packing motif is observed. As anticipated, neither edge‐to‐face nor strong face‐to‐face interactions between two anthracenes are possible due to the twisted orientation of that substituent. C−H⋅⋅⋅π interactions in the range of 2.778 Å and 2.872 Å between phenyl groups at the phosphorous and anthracene and between the outer rings of the anthracene moieties are the dominant non‐covalent contacts (Figure S19). Compared to the former discussed structures the anthracene is nearly prevented from any stronger interchromophoric interactions by the two bulky substituents (Figure 4 c).

**Table 2:** Structural parameters of thiophosphoranyl anthracenes **5**–**8** and host guest co‐crystals **7 a**–**7 d**.

	Overlap [%]	d_π–π_ [Å]	d_x_ [Å]	d_y_ [Å]
**5**	21.1	3.242	1.344	3.227
**6**	33.3	3.372	1.056	2.450
**7**	42.8	3.386	0.603	2.814
**7 a**	22.2	3.370	1.478	1.634
**7 b**	22.9	3.405	1.468	1.552
**7 c**	16.9	3.405	1.652	1.384
**7 d**	0	–	–	–
**8**	0	–	–	–

During the investigation of [9‐(S)PPh_2_‐10‐Et‐(C_14_H_8_)] (**7**) we discovered the ability of the latter to form host–guest co‐crystals with small arenes when they were used as solvent for crystallisation. In addition to the solvent free structure, which was obtained by recrystallisation from ethyl acetate, four host–guest complexes with one molecule of **7** and one benzene (**7 a**), pyridine (**7 b**), toluene (**7 c**) and quinoline (**7 d**) each, present in the asymmetric unit, were obtained. For the smaller arenes benzene, pyridine and toluene almost identical orientations of the hosts relative to the guest were obtained (Figure S22).

**Figure 5:**
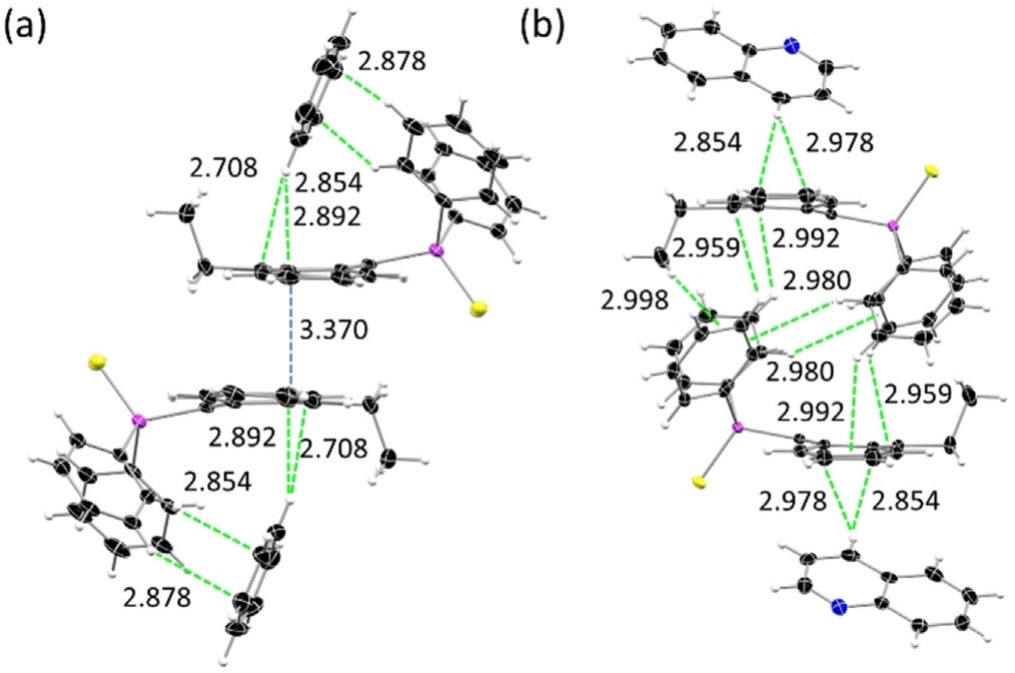
Molecular structure of the host–guest complex of C_6_H_6_@[9‐(S)PPh_2_‐10‐Et‐(C_14_H_8_)] (**7 a**). The benzene molecule is located inside the cradle formed by the diphenyl‐ and ethyl groups and hold in position by several weak C−H⋅⋅⋅π‐interactions (green). (b) The larger quinoline of the host–guest complex **7 d** does not fit inside the pocket and is located on the opposite site of the anthracene plane.

**Figure 6:**
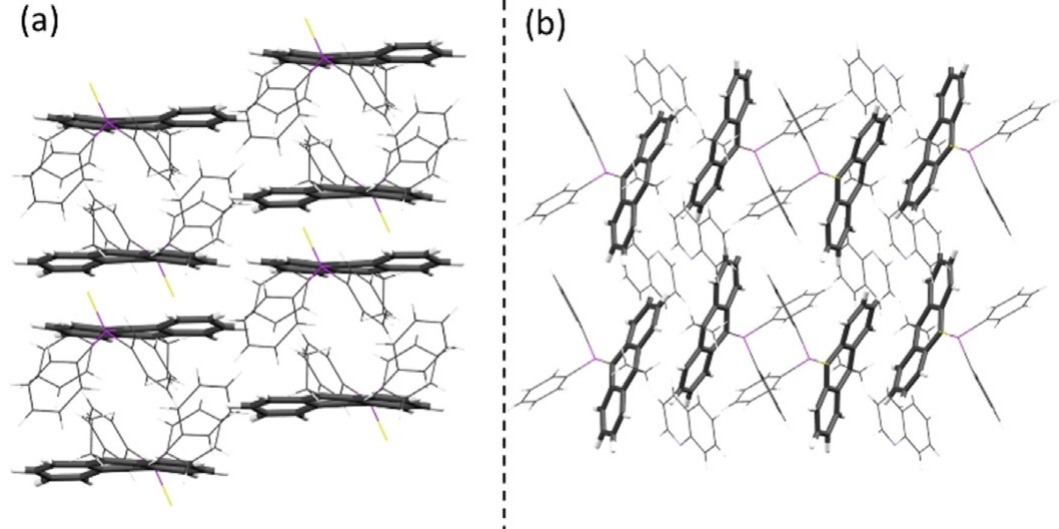
(a) Crystal packing of the anthracene moieties as found in the co‐crystals **7 a**—**7 c** and (b) changed orientation of the anthracenes in **7 d**.

The phenyl groups of the substituent at the 9‐position and the ethyl group in the 10‐position form a cradle in which the solvent is embedded above one outer anthracene ring (Figure 5 a). The aromatic planes of the solvent and of the anthracene enclose an angle of about 65°. The molecular structure is affected slightly by the inclusion of the solvent. The strongest influence manifests itself in the folding angle α of the anthracene which is significantly decreased with values between 7.78(11)° and 9.42(11)°. Hence, a more planar structure is obtained compared to the solvent free structure of **7** (Table 2, Figure S20, S23). The dimeric structure and the face‐to‐face interactions are still present, but the incorporation of the solvent induces a decrease of the overlapping area to 17–23 %. Remarkably, the offset of the overlapping anthracenes along the long molecular anthracene axes (d_y_) is clearly reduced in all co‐crystals due to the more planar anthracene. The offset along the short axes is increased, resulting in an overall smaller overlapping area. The crystal packing also changes upon co‐crystallisation of the solvent. For **7 a**–**7 c** a sheet structure with all anthracenes parallel oriented is obtained (Figure 6 a, S24–S25). When quinoline is co‐crystallised further changes in the structure occur. Probably due to its larger size it does not fit inside the cradle anymore and a different position related to the anthracene is adopted. The guest molecule now is located at the opposite side of the anthracene plane (Figure 5 b). The quinoline plane encloses a smaller angle with the anthracene core of about 35 °and is closer to a plan‐parallel orientation. Therefore, inter‐host face‐to‐face interactions are neglected and only C−H⋅⋅⋅π interactions between the H4 of the quinoline and the anthracene π‐system are present. Again, a change in the crystal packing of the anthracene scaffolds is observed (Figure 6 b). It should be noted that the benzene and the toluene hosts in **7 a** and **7 c**, respectively, adopt a minor disorder and a second position can be found, generated by a rotation about the C_6_‐perimeter midpoint of about 60 °.

Varying the substituents in the 10‐position causes different packing motifs with strong edge‐to‐face, face‐to‐face and only minute interchromophoric interactions. For **7** four co‐crystals could be obtained which also lead to a change in the crystal packing and the dominant interactions. Therefore, a suitable set of structures is obtained to investigate the structure‐property relationship without changing the electronic structures of the fluorophores substantially.

### Photophysical Properties

The absorption spectra of **1**–**4** in diluted THF solution are dominated by the parent anthracene. A higher energetic absorption around 260 nm and a structured absorption band in the range of 340 to 420 nm are present (Figure S42). The absorption of **1** is red‐shifted of about 20 nm compared to unsubstituted anthracene. Introduction of a second substituent leads to a further shift of around 10 nm. The lower energy absorption is assigned to the S_0_→S_1_ transition and of typical π→π* character as known for many anthracene derivatives. A possible charge transfer from the phosphane lone pair could not be observed. No further absorption at higher wavelengths, which has been reported for the S_0_→^1^CT transition of the corresponding diphenylamino anthracene, was detected.^[37]^

**Figure 7:**
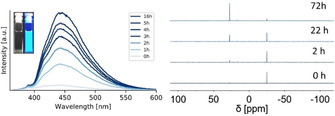
Emission spectra of **1** in a non‐degassed THF solution (10^−5^ m) recorded over a period of 16 h reveal an increase in intensity due to oxidation of phosphorus. The inset shows the solution after 16 h in daylight (left) and under UV irradiation (right). The slow oxidation can be monitored via time‐dependent ^31^P‐NMR spectroscopy in non‐degassed [D_8_]THF. The decrease of the signal around −25 ppm and the increase of the signal of the oxidation product (+30 ppm) go along with the increasing of the emission intensity.

The emission of the phosphanyl anthracenes **1**–**4** is nearly completely quenched in solution and in the solid‐state, which is remarkable as the amine homolog of **1** reveals a bright emission at least in solution (Φ_F_=0.92^35^). The intense fluorescence of the amine is attributed to the S_0_←^1^CT emission.^[37]^ However, in non‐degassed THF solution an increase of the emission intensity over time can be observed (Figure 7). The P(iii) gets oxidised slowly, which leads to an intense blue emission of the oxidised species, which has been reported earlier.^[38]^ The oxidation process can be monitored via ^31^P‐NMR spectroscopy and shows a slow decrease of the original signal at −25 ppm and an arising signal around +30 ppm, which can be assigned to the oxidation product [9‐(O)PPh_2_(C_14_H_9_)]. The deviation in the time scale is due to different used concentration as the emission spectra were measured in very diluted solution (10^−5^ m) to avoid self‐quenching effects and leads probably to a faster oxidation then the more concentrated NMR‐sample. The oxophosphoranyl anthracenes are highly emissive in solution but emit only weakly in the solid‐state and were therefore not further investigated.[^30^, ^39^]

**Figure 8:**
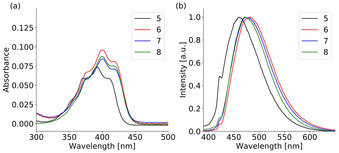
(a) UV/VIS spectra of thiophosphoranyl anthracenes (**5**–**8**) in diluted THF solution (10^−5^ m). Only the relevant region of the S_0_→S_1_ transition is shown. (b) Normalized emission spectra of **5**–**8** in diluted THF solution (10^−5^ m, λ_ex_=375 nm). The small shoulder at around 420 nm originates from scattering of the solvent as it clearly shifts with the excitation wavelength.

**Figure 9:**
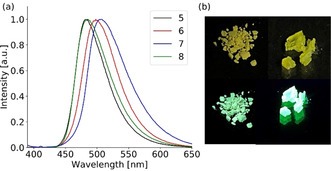
(a) Normalized solid‐state emission spectra of thiophosphoranyl anthracenes **5**–**8** (λ_ex_=375 nm). (b) A sample of **8** (left) and crystals of **7 c** (right) under daylight (bottom) and UV‐light (top).

The absorption spectra of the sulfur‐oxidation products **5**–**8** in diluted solution are comparable to the parent phosphanes (Figure 8). Two bands are visible in the spectra and the lower energy vibronic bands are assigned to the S_0_→S_1_ transition located at the anthracene. They experience a further bathochromic shift of 10–15 nm upon oxidation. In the emission spectra the vibronic structure is completely lost and only one broad, unstructured emission band is observed. The emission wavelengths peak in the range of 462–480 nm and range up to 550 nm. The small bathochromic shift of **6**, **7** and **8** is attributed to the inductive effect of the second substituent in the 10‐position. Even if the emission band is almost unstructured (the small shoulder at 420 nm originates from scattering of the solvent) a charge transfer character is still unlikely as the emission wavelength is not affected by the solvent polarity. The obtained lifetimes are within a few nanoseconds and in the typical range of fluorescence emission from the S_1_ state (Table S17). The broad unstructured emission and the bathochromic shift up to 480 nm are quite unusual for in‐solution emission of simple anthracene derivatives, which usually emit in the region from 380–450 nm. Since a charge transfer was excluded the strong deformation of the anthracene plane is probably responsible for the unusual unstructured, broad and red‐shifted emission in solution as was also assumed earlier.^[33]^ The recently reported positional isomers of **5** revealed a nearly planar anthracene core and therefore a typical vibronic emission band, which was also less red‐shifted.^[40]^ The low quantum yields can be explained by a photoinduced electron transfer (PET) from the sulfur lone pairs towards the anthracene π‐system, which leads to a strong fluorescence quenching. In the solid‐state all compounds exhibit a bright green emission after UV irradiation, which is obvious even for the naked eye (Figure 9). The emission is still unstructured, which is not unusual in the solid‐state.

Compared to the emission in solution a further bathochromic shift between 20 and 30 nm is observed and the emission peaks between 484 and 506 nm. The lifetimes are only slightly affected and are still in the range of typical singlet‐state fluorescence emission (Table 3). The shape of the spectra is comparable to the solution‐state and the broadening is even less pronounced, which can be quantified by a smaller full width at half maximum (Table S18). The only minor changes of the photophysical properties between the solution‐ and solid‐state exclude a strong influence of the packing on the emission wavelengths. Even the dimeric stacking of **6** and **7**, which reveal the typical geometry for excimer emission, has only little influence because the emission wavelength is shifted by only 10–20 nm, compared to **8**, which shows no π–π interactions. Although the overlap of the anthracene dimers reaches values up to 43 % the formation of excimers is not likely. The evaluation of the photophysical properties in solution and in the solid‐state indicate that the emission in the solid‐state is ascribed to a monomer emission. This is remarkable as emission wavelengths in the green region around 500 nm are rare for small anthracene molecules, when no CT or excimer emission is present. The strong deformation induced by the bulky substituent, which was evaluated above, is probably responsible for this unusual bathochromic shift and green emission around 500 nm. The strong intermolecular interactions and short interchromophoric distances in **5**–**7** result in effective radiationless pathways and therefore in lower quantum yields. The introduction of the bulkier phenyl group as substituent in the 10‐position decreases the interaction between two fluorophores and a more intense monomer emission is observed for **8** with quantum yields up to 25.4 %.

**Table 3:** Photophysical data of thiophosphoranyl anthracenes (**5**–**8**) and host guest‐complexes (**7 a**–**7 d**) of [9‐(S)PPh_2_‐10‐Et‐(C_14_H_8_)] (**7**) in the solid‐state.

	λ_em_^[a]^ [nm]	τ^[b]^ [ns]	Φ_F_^[c]^ [%]	k_r_ [μs^−1^]	k_nr_ [μs^−1^]
**5**	484	2.1	4.0±1.0	19.1	457.1
**6**	495	2.4	4.7±0.2	19.6	397.1
**7**	506	4.9	5.2±0.4	10.6	192.7
**7 a**	485	6.4	23.3±0.8	36.4	119.8
**7 b**	489	5.8	14.6±1.0	25.1	147.0
**7 c**	480	5.6	22.2±0.7	39.6	138.9
**7 d**	494	4.9	18.2±1.2	36.5	163.9
**8**	498	8.4	25.4±0.8	30.2	88.8

[a] λ_ex_=375/400 nm. [b] λ_ex_=375 nm, emission detected at λ_em_.[c] For **7 a**–**7 d** the initial value was taken (see text for details).

**Figure 10:**
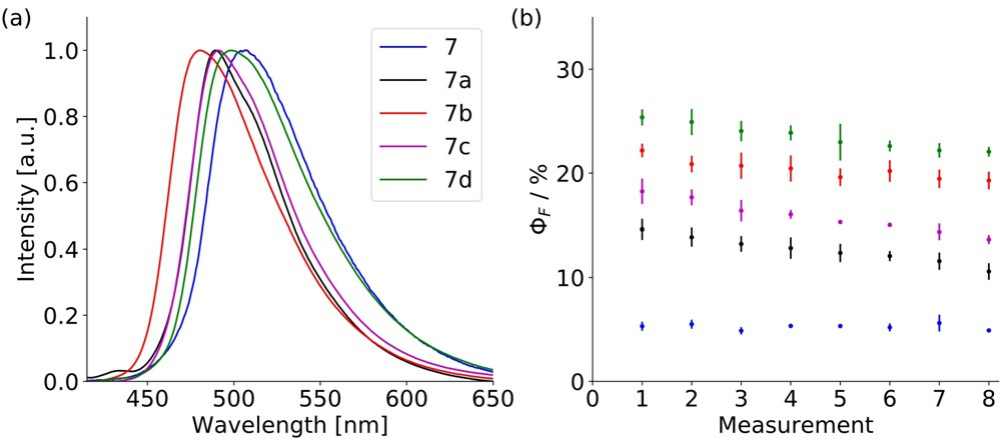
(a) Normalized solid‐state emission spectra of **7** and **7 a**–**7 d** (λ_ex_=400 nm). (b) Quantum yield decay of **7** and **7 a**–**7 d** over time in subsequent measurements (same colour code as (a)).

With this observation in mind, we investigated the co‐crystals **7 a**–**7 d**. The emission wavelengths are only little affected upon co‐crystallisation of various arenes and show only a minute blue shifted emission, which goes along with the less distorted anthracene plane (**7 a**–**7 c**) and decreasing π‐π interactions due to the lower overlap of the anthracenes (Figure 10). At first sight counterintuitively, the quantum yields Φ_F_ [%] of the co‐crystals are about three to five times higher than the obtained values for pure **7** and reach values up to 23.3 %. We assign two factors to be responsible for the observed emission enhancement upon co‐crystallisation: (i) The changed crystal packing to a sheet structure gives less interchromophoric interactions. Especially in **7 d** the strong face‐to‐face interactions and the dimeric stacking vanishes upon quinoline co‐crystallisation. (ii) Secondly, the co‐crystallisation of the arenes inside the formed cradle imposes several weak C−H⋅⋅⋅π interactions between the guest and the anthracene as well as the phenyl groups of the host molecule. This reduces the intramolecular motion of the host considerably and potential radiationless pathways are blocked. As the consequence the radiative rates k_r_ of the host–guest complexes are increased and non‐radiative rates k_nr_ are reduced compared to **7**. These two effects result overall in higher quantum yields in the solid‐state. As emission wavelengths and lifetimes are only slightly affected an exciplex mechanism for emission enhancement is not likely.

Evaluating the quantum yields of the host–guest complexes **7 a**–**7 d** a slight decay was detected over acquisition time (Figure 10 b). This observation further confirms the importance of the co‐crystallised solvent. The slow decrease of the quantum yields can be explained by slow evaporation of the guest molecules from the crystal lattice. Their transient to the gas phase increases the internal motion again and hence also the rate of the non‐radiative decay. The release of the solvent is promoted further by the irradiation during the measurements. Furthermore, it is hard to obtain the exact same measuring conditions as the amount of evaporated solvent during the preparation of the sample (crystal picking, drying, grinding), is hard to determine.

With the obtained data an influence of the volatility and/or disorder on the decay rate of the quantum yield could not be investigated. Therefore, further studies with a similar disubstituted system, which co‐crystallises with a lot more solvents will facilitate a more detailed view into the underlying processes of evaporation and the role of the solvent.

## Conclusions

We synthesized and characterized four structurally simple anthracene phosphanes and their sulfur oxidised products. The oxidation causes drastic changes in the molecular structure. The two phenyl groups are now both located at the same side of the anthracene plane and the bulky substituent induces a strong butterfly bent structure of the anthracene core. The deformation of the aromatic plane triggers a strong bathochromic shift of the emission wavelength, which resulted in a green emission in the solid‐state. Analyses of different crystal packing motifs could show that the emission wavelength is just slightly affected by the intermolecular interactions. As the emission spectra are quite similar in solution and in the solid‐state the origin of the emission is attributed to a typical monomer fluorescence, which further only gives a nanosecond lifetime. Solid‐state emission for structurally simple anthracene compounds around 500 nm, which does not result from an excimer or charge‐transfer process and is attributed to monomer emission is quite rare. The host–guest complexes of **7** with four small aromatic molecules reveal an emission enhancement and up to five‐times higher quantum yields compared to the pure host. Less interchromophoric interactions and a restriction of intramolecular motion within the host molecules due to fixation by weak C−H⋅⋅⋅π interactions with the co‐crystallised arene are made responsible for the emission enhancement. The concept of co‐crystallisation induced enhanced emission will further be evaluated with a similar system which co‐crystallises with a wider range of arenes and also in different host/guest ratios, which allows a more detailed analysis.

## Experimental Section

Reactions using air‐ and moisture sensitive compounds were performed under an atmosphere of N_2_ or Ar using standard Schlenk techniques.^[41]^ Solvents were dried with standard techniques. Commercially available 9‐bromoanthracene and 9‐bromo‐10‐phenylanthracene were purchased and used without further purification. Chlorodiphenylphosphane was distilled before use. Elemental sulfur was purified by sublimation. 9,10‐dibromoanthracene,^[42]^ 9‐bromo‐10‐methylanthracene and 9‐bromo‐10‐ethylanthracene were synthesized according to literature procedures.^[43]^ UV/Vis and fluorescence measurements were performed in analytical grade solvents. NMR spectroscopic data were recorded on a Bruker Avance 400 MHz and a Bruker Avance 300 MHz spectrometer and referenced to the deuterated solvent. EI mass spectra were recorded using a *MAT 95* device with electron ionization (EI‐MS: 70 eV). ESI spectra were obtained from a BRUKER micrOTOF instrument. Elemental analyses (C, H, S) were carried out on a Vario EL3 at the Mikroanalytisches Labor, Institut für Anorganische Chemie, University of Göttingen. UV/Vis spectra were recorded on an Agilent Cary 50 spectrometer using quartz cuvettes. Fluorescence measurements were carried out on a Horiba Jobin–Yvon Fluoromax‐4 spectrometer equipped with a 150 W xenon arc lamp as excitation source and a photomultiplier as detector. A front‐face setup was used for collecting emission spectra of solid samples. Absolute quantum yields were determined with the Quanta‐ϕ integrating sphere. Lifetime measurements were performed with the TCSPC setup using a 375 nm pulsed laser diode.

Crystallographic data were collected with an Ag (for **3**) or Mo‐IμS microfocus source.^[44]^ All data were integrated with SAINT.^[45]^ A multiscan absorption correction (SADABS)^[46]^ and a 3 λ correction^[32]^ (except for **3**, **7 a** and **7 c**) was applied. The structures were solved by direct methods (SHELXT)^[47]^ and refined on *F* ^2^ using the full‐matrix least‐squares methods of SHELXL^[48]^ within the ShelXle GUI.^[49]^ Disordered groups were modelled with DSR.^[50]^ Deposition Numbers 1991516, 1991517, 1991518, 1991519, 1991520, 1991521, 1991522, 1991523, 1991524, 1991525, 1991526, 1991527 contain the supplementary crystallographic data for this paper. These data are provided free of charge by the joint Cambridge Crystallographic Data Centre and Fachinformationszentrum Karlsruhe Access Structures service www.ccdc.cam.ac.uk/structures.

General procedure for the preparation of diphenylphosphenylanthracenes **1**–**4**: The corresponding bromoanthracene (1.0 equiv.) was dissolved in Et_2_O and cooled to −78 °C. A solution of *n*BuLi in hexane (2.2 m, 1.05 equiv.) was added dropwise over 10 min. The mixture was stirred for another 30 min and then chlorodiphenylphosphane (1.05 equiv.) was added dropwise over 10 min. The mixture was allowed to warm to ambient temperature overnight, while a yellow solid precipitated, which was filtered off. The solvent from the filtrate was evaporated, and the residue was dissolved in DCM or toluene and filtered again. Finally, the solvent was evaporated, and the solids combined and washed with hexane. Recrystallisation from DCM or toluene gave the target compounds as yellow crystals.

General procedure for the oxidation to the thiophosphoranyl anthracenes **5**–**8**: The corresponding diphenylphosphanylanthracene (1.0 equiv.) (**1**–**4**) was dissolved in toluene together with elemental sulfur (1.5 equiv.) and heated to 80 °C for 6 h. Afterwards the solvent was removed under reduced pressure and the residue recrystallised from toluene or EtOAc. The desired products were obtained as yellow crystals.

General procedure for preparation of host–guest complexes (**7 a**–**7 d**): [9‐(S)PPh_2_‐10‐Et‐(C_14_H_8_)] (**7**) was recrystallised from the corresponding aromatic solvent. Slow cooling overnight afforded crystals with included solvent. Unit cells of several crystals were determined for verification of successful co‐crystallisation.

Analytical data of the compounds **1**–**8** can be found in the Supporting Information.

Theoretical calculations were carried out using the Gaussian16 software package. For geometry optimization the B3LYP^[51]^ functional was employed, using the def2‐TZVP basis set,^[52]^ with dispersion corrected for by Grimme's empirical D3‐correction.^[53]^ Coordinates of the optimised structures are given in the Supporting Information.

## Conflict of interest

The authors declare no conflict of interest.

## Supporting information

As a service to our authors and readers, this journal provides supporting information supplied by the authors. Such materials are peer reviewed and may be re‐organized for online delivery, but are not copy‐edited or typeset. Technical support issues arising from supporting information (other than missing files) should be addressed to the authors.

Supplementary
