# Supplementary material for: Analysis of Solid‐State Luminescence Emission Amplification at Substituted Anthracenes by Host–Guest Complex Formation
Source: Chemistry. 2020 Oct 27;26(72):17390–8. doi: 10.1002/chem.202003017 (PMC7821099; doi:10.1002/chem.202003017)
Supplement: Supplementary file 1 — Supplementary [file CHEM-26-17390-s001.pdf]

Chemistry–A European Journal

Supporting Information

Analysis of Solid-State Luminescence Emission Amplification at Substituted Anthracenes by Host–Guest Complex Formation

Timo Schillmöller, Paul Niklas Ruth, Regine Herbst-Irmer, and Dietmar Stalke*[a]

Content:

S1. Analytical Data

S2. X-Ray Crystallographic Analysis

S3. Photophysical Data

S4. Computations

S5. NMR Spectroscopic Data

S6. References

S1. Analytical data

[9-PPh$_2$(C$_{14}$H$_9$)] (1)

Yield 1.03 g (73%). Yellow crystals. ^{1}H NMR (300 MHz, CDCl$_3$): δ [ppm] = 8.79 (dd, $^{3}J_{HH}$ = 8.8 Hz, $^{4}J_{PH}$ = 4.6 Hz, 2H, H$_{1,8}$), 8.67 (s, 1H, H$_{10}$), 8.07 (dd, $^{3}J_{HH}$ = 8.4 Hz, 2H, H$_{4,5}$), 7.49 – 7.41 (m, 6H, *o*-Ph, H$_{3,6}$), 7.37 – 7.32 (m, 2H, H$_{2,7}$), 7.30 – 7.26 (m, 6H, *m*-Ph, *p*-Ph). ^{13}C{^{1}H} NMR (75 MHz, CDCl$_3$): δ [ppm] = 137.0 (d, $^{3}J_{CP}$ = 6.0 Hz, 2C, C$_{4a,10a}$), 136.8 (d, $^{2}J_{CP}$ = 6.8 Hz, 2C, C$_{8a,9a}$), 132.1 (s, 1C, C$_{10}$), 132.0 (d, $^{1}J_{CP}$ = 4.5 Hz, 2C, *i*-Ph), 131.7 (d, $^{2}J_{CP}$ = 18.3 Hz, 4C, *o*-Ph), 129.6 (s, 1C, C$_9$), 129.4 (s, 2C, C$_{4,5}$), 128.7 (d, $^{3}J_{CP}$ = 24.8 Hz, 2C, C$_{1,8}$), 128.5 (d, $^{3}J_{CP}$ = 5.7 Hz, 4C, *m*-Ph), 127.7 (s, 2C, *p*-Ph), 126.1 (s, 2C, C$_{2,7}$), 125.2 (s, 2C, C$_{3,6}$). ^{31}P{^{1}H} NMR (121 MHz, CDCl$_3$): δ [ppm] = -24.69 (s). HRMS (EI): calcd for C$_{26}$H$_{19}$P: 363.1291, found: 363.1297. Elemental Analysis: calcd (%): C: 86.2, H: 5.28; found: C: 85.0, H: 5.35.

[9-PPh$_2$-10-Me-(C$_{14}$H$_8$)] (2)

Yield 0.55 g (70%). Yellow crystals. ^{1}H NMR (300 MHz, CD$_2$Cl$_2$): δ [ppm] = 8.83 (dd, $^{3}J_{HH}$ = 8.9 Hz, $^{4}J_{HH}$ = 1.2 Hz, 2H, H$_{4,5}$), 8.41 (ddd, $^{3}J_{HH}$ = 8.9 Hz, $^{4}J_{PH}$ = 3.0 Hz, $^{4}J_{HH}$ = 1.2 Hz, 2H, H$_{1,8}$), 7.49 (ddd, , $^{3}J_{HH}$ = 8.9 Hz, $^{3}J_{HH}$ = 6.4 Hz, $^{4}J_{HH}$ = 1.2 Hz, 2H, H$_{2,7}$), 7.43 – 7.37 (m, 4H, *o*-Ph), 7.32 (ddd, $^{3}J_{HH}$ = 8.9 Hz, $^{3}J_{HH}$ = 6.4 Hz, $^{4}J_{HH}$ = 1.3 Hz, 2H, H$_{3,6}$), 7.27 – 7.22 (m, 6H, *m*-Ph, *p*-Ph), 3.21 (s, 3H, C*H*$_3$). ^{13}C{^{1}H} NMR (75 MHz, CD$_2$Cl$_2$): δ [ppm] = 137.7 (d, $^{1}J_{CP}$ = 14.7 Hz, 2C, *ipso*-Ph), 136.8 (d, $^{2}J_{CP}$ = 12.7 Hz, 2C, C$_{8a,9a}$), 136.6 (d, $^{3}J_{CP}$ = 1.6 Hz, 2C, C$_{4a,10a}$), 131.8 (d, $^{2}J_{CP}$ = 18.5 Hz, 2C, *o*-Ph), 131.2 (s, 1C, C$_{10}$), 129.7 (d, $^{4}J_{CP}$ = 25.6 Hz, 2C, C$_{4,5}$), 128.9 (d, $^{3}J_{CP}$ = 5.6 Hz, 4C, *m*-Ph), 128.0 (s, 2C, *p*-Ph), 127,1 (d, $^{1}J_{CP}$ = 18.6 Hz, 1C, C$_9$), 126.1 (s, 2C, C$_{1,8}$), 125.6 (d, $^{4}J_{CP}$ = 1.7 Hz, C$_{2,7}$) , 15.3 (s, 1C, *C*H$_3$). ^{31}P{^{1}H} NMR (121 MHz, CD$_2$Cl$_2$): δ [ppm] = -24.30 (s). HRMS (EI): calcd for C$_{27}$H$_{21}$P: 376.1381, found: 376.1373. Elemental Analysis: calcd (%): C: 86.15, H: 5.62; found: C: 83.96, H: 5.64.

[9-PPh$_2$-10-Et-(C$_{14}$H$_8$)] (3)

Yield 2.06 g (87%). Yellow crystals. ^{1}H NMR (300 MHz, CDCl$_3$): δ [ppm] = 8.90 (dd, J_{HH} = 8.8, 5.3 Hz, 2H, H$_{1,8}$), 8.41 (dd, J_{HH} = 8.8 Hz, 1.1 Hz, 2H, H$_{4,5}$), 7.53 – 7.43 (m, 6H, H$_{3,6}$, *o*-Ph), 7.37 – 7.25 (m, 8H, H$_{2,7,}$ *m*-Ph, *p*-Ph), 3.77 (q, $^{3}J_{HH}$ = 7.6 Hz, 2H, C*H*$_2$), 1.59 (t, $^{3}J_{HH}$ = 7.6 Hz, 3H, C*H*$_3$). ^{13}C{^{1}H} NMR (75 MHz, CDCl$_3$): δ [ppm] = 141.8 (d, $^{4}J_{CP}$ = 1.9 Hz, 1C, C$_{10}$), 137.0 (d, $^{1}J_{CP}$ = 14.3 Hz, *i*-Ph), 136.3 (d, $^{1}J_{CP}$ = 13.0 Hz, C$_9$), 131.5 (d, $^{2}J_{CP}$ = 18.3 Hz, 4C, *o*-Ph), 129.6 (d,, $^{3}J_{CP}$ = 6.2 Hz, 2C, C$_{1,8}$)129.3 (s, 2C, C$_{4a,10a}$) 128.4 (d, $^{3}J_{CP}$ = 5.5 Hz, 4C, *m*-Ph), 127.4 (s, 2C, C$_{3,6}$), 126.7 (d, J$_{CP}$ = 18.5 Hz, C$_{8a,9a}$), 125.3 (d, $^{4}J_{CP}$ = 1.8 Hz, 2C, C$_{2,7}$), 125.2 (d, $^{4}J_{CP}$ = 1.6 Hz, 2C, p-Ph), 125.0 (s, 2C, C$_{4,5}$), 21.9 (s, *C*H$_2$), 15.5 (s, *C*H$_3$). ^{31}P{^{1}H} NMR (121 MHz, CDCl$_3$): δ [ppm] = -24.13 (s). HRMS (ESI^{+}): calcd for C$_{28}$H$_{24}$P^{+} [M+H]$^{+}$: 391.1610, found: 391.1602 Elemental Analysis: calcd (%): C: 86.13, H: 5.94; found: C: 85.63, H: 5.94.

[9-PPh$_2$-10-Ph-(C$_{14}$H$_8$)] (4)

Yield 0.37 g (84%). Yellow crystals. ^{1}H NMR (300 MHz, C$_6$D$_6$): δ [ppm] = 91 – 8.87 (m, 2H, H$_{4,5}$), 7.73 – 7.71 (m, 2H, H$_{1,8}$), 7.66 – 7.59 (m, 3H, H$_{12,14,16}$), 7.52 – 7.48 (m, 6H, H$_{13,15}$, *o*-Ph), 7.34 – 7.27 (m, 10H, H$_{2,3,6,7,}$ *m*-Ph, *p*-Ph). ^{13}C{^{1}H} NMR (75 MHz, C$_6$D$_6$): δ [ppm] = 141.9 (s, 1C, C$_{10}$), 138.9 (s, 1C, C$_{11}$), 136.8 (d,$^{1}J_{CP}$ = 14.2 Hz, 1C, C$_9$), 136.9 (d, $^{1}J_{CP}$ = 13.1 Hz, 2C, *i*-Ph), 131.7 (d, $^{2}J_{CP}$ = 18.3 Hz, 4C, *o*-Ph), 131.0 (s, 2C, C$_{4a,10a}$), 130.5 (d, $^{2}J_{CP}$ = 4.5 Hz, 2C, C$_{8a,9a}$), 128.7 (s, 2C, C$_{4.5}$), 128.6 (d, $^{3}J_{CP}$ = 5.3 Hz, 4C, m-Ph), 128.2 (s, 2C, C$_{13,15}$), 127.9 (s, 3C, C$_{12,14,16}$), 127.6 (d, $^{2}J_{CP}$ = 12.0 Hz, 2C, C$_{1,8}$), 125.5 (s, 2C, C$_{2,7}$), 124.9 (s, 2C, C$_{3,6}$). ^{31}P{^{1}H} NMR (121 MHz, C$_6$D$_6$): δ [ppm] = -24.11 (s). HRMS (ESI^{+}): calcd for C$_{32}$H$_{24}$P^{+}[M+H]$^{+}$: 439.1610, found: 439.1612. Elemental Analysis: calcd (%): C: 87.65, H: 5.20; found: C: 86.41, H: 5.20.

[9-(S)PPh$_2$(C$_{14}$H$_9$)] (5)

Yield 0.84 g (96 %). Yellow crystals. ^{1}H NMR (300 MHz, CDCl$_3$),): δ [ppm] = 8.66 (s, 1H, H_{10}), 8.05 – 7.96 (m, 4H, $H_{1,8}$, $H_{4,5}$), 7.87 – 7.75 (m, 4H, *o*-Ph), 7.40 – 7.25 (m, 8H, $H_{2,7}$, *m*-Ph, *p*-Ph), 7.10 – 7.03 (m, 2H, $H_{3,6}$). ^{13}C{^{1}H} NMR (75 MHz, CDCl$_3$): δ [ppm] =137.2 (d, $^1J_{CP}$ = 82.5 Hz, 2C, $_{ipso}$-Ph), 133.8 (d, $^4J_{CP}$ = 3.8 Hz, 1C, C_{10}), 133.4 (d, $^2J_{CP}$ = 8.0 Hz, 2C, $C_{8a,9a}$), 131.6 (d, $^3J_{CP}$ = 11.2 Hz, 2C, $C_{4a,10a}$), 131.0 (d, $^2J_{CP}$ = 10.5 Hz, 4C, *o*-Ph), 130.7 (d, $^4J_{CP}$ = 3.0 Hz, 2C, *p*-Ph), 129.2 (s, 2C, $C_{4,5}$), 128.6 (d, $^3J_{CP}$ = 12.6 Hz, 4C, *m*-Ph), 127.5 (d, $^3J_{CP}$ = 10.0 Hz, 2C, $C_{1,8}$), 125.8 (s, 2C, $C_{3,6}$), 125.2 (s, 2C, $C_{2,7}$), 122.7 (d, $^1J_{CP}$ = 87.3 Hz, 1C, C_9). ^{31}P{^{1}H} NMR (121 MHz, CDCl$_3$): δ [ppm] = 34.2 (s). HRMS (EI): calcd for $C_{26}H_{19}PS$: 394.0945, found: 394.0943. Elemental Analysis: calcd (%): C: 79.17, H: 4.86, S: 8.63; found C: 77.09, H: 4.83, S: 8.13.

[9-(S)PPh$_2$-10-Me-(C$_{14}$H$_8$)] (6)

Yield 0.20 g (92 %). Yellow crystals. ^{1}H NMR (300 MHz, CDCl$_3$),) δ [ppm] = 8.34 – 8.31 (m, 2H, $H_{4,5}$), 8.10 – 8.07 (m, 2H, $H_{1,8}$), 7.79 – 7.72 (m, 4H, *o*-Ph), 7.40 – 7.35 (m, 2H, $H_{3,6}$), 7.36 – 7.22 (m, 6H, *m*-Ph, *p*-Ph), 7.07 – 7.02 (m 2H, $H_{2,7}$), 3.20 (s, 3H, C*H*$_3$). ^{13}C{^{1}H} NMR (75 MHz, CDCl$_3$): δ [ppm] = 137.8 (d, $^4J_{CP}$ = 4.1 Hz, 2C, $C_{4a,10a}$), 137.3 (d, $^1J_{C,P}$ = 82.8 Hz, 2C, *i*-Ph), 133.0 (d, *J* = 7.6 Hz,2C, $C_{8a,9a}$), 130.8 (d, $^2J_{C,P}$ = 10.3 Hz, 2C, *o*-Ph), 130.4 (d, $^4J_{C,P}$ = 2.9 Hz, 2C, *p*-Ph), 130.2 (s,C_{10}), 128.3 (d, $^3J_{C,P}$ = 12.5 Hz, 2C, *m*-Ph), 128.0 (d, $^3J_{C,P}$ = 10.7 Hz, $C_{1,8}$), 125.0 (m, 6C, $C_{2,3,4,5,6,7}$), 120.7 (d, $^1J_{C,P}$ = 92.1 Hz, C_9), 15.3 (s, *C*H$_3$). ^{31}P{^{1}H} NMR (121 MHz, CDCl$_3$): δ [ppm] = 34.44 (s). HRMS (EI): calcd for $C_{27}H_{21}PS$: 408.1102, found: 408.1110. Elemental analysis: calcd (%): C: 79.39, H: 5.18, S: 7.58; found: C: 78.53, 5.21, 7.90.

[9-(S)PPh$_2$-10-Et-(C$_{14}$H$_8$)] (7)

Yield 0.43 g (77 %). Yellow crystals. ^{1}H NMR (300 MHz, [D$_8$]THF): δ [ppm] = 8.38 (dd, $^3J_{HH}$ = 8.9 Hz, $^4J_{PH}$ = 2.2 Hz, 2H, $H_{1,8}$), 8.09 (d, $^3J_{HH}$ = 8.9 Hz, 2H, $H_{4,5}$), 7.75 (dd, $^3J_{HH}$ = 8.0 Hz, $^4J_{HH}$ = 1.4 Hz, 4H, *o*-Ph), 7.37 (dd, $^3J_{HH}$ = 8.9 Hz, $^3J_{HH}$ = 6.5 Hz, 2H, $H_{2,7}$), 7.30 – 7.19 (m, 6H, *m*-Ph, *p*-Ph), 6.99 (dd, $^3J_{HH}$ = 8.9 Hz, $^3J_{HH}$ = 6.5 Hz, 2H, $H_{3,6}$), 3.75 (q, $^3J_{HH}$ = 7.5 Hz, 2H, C*H*$_2$), 1.52 (t, $^3J_{HH}$ = 7.5 Hz, 3H, C*H*$_3$). ^{13}C{^{1}H} NMR (75 MHz, [D$_8$]THF): δ [ppm] =144.5 (d, $^3J_{CP}$ = 3.9 Hz, 2C, $C_{4a,10a}$), 139.5 (d, $^1J_{CP}$ = 82.3 Hz, 2C, *ipso*-Ph,), 134.2 (d, $^2J_{CP}$ = 7.7 Hz, 2C, $C_{8a,9a}$), 131.8 (d, $^2J_{CP}$ = 10.2 Hz, 2C, *o*-Ph), 131.1 (d, $^4J_{CP}$ = 2.9 Hz, 2C, *p*-Ph), 130.4 (d, $^4J_{CP}$ = 10.9 Hz, 1C, C_{10}), 129.4 (d, $^4J_{CP}$ = 10.8 Hz, 2C, $C_{4,5}$), 129.2 (d, $^3J_{CP}$ = 12.4 Hz, 4C, *m*-Ph), 126.1 (s, 2C, $C_{2,7}$), 125.7 (s, 2C, $C_{1,8}$), 125.5 (s, 2C, $C_{3,6}$), 124.0 (d, $^1J_{CP}$ = 90.9 Hz, 1C, C_9), 23.0 (s, 1C, CH_2), 16.2 (s, 1C, CH_3). ^{31}P{^{1}H} NMR (121 MHz, [D$_8$]THF): δ [ppm] = 31.69 (s). HRMS (EI): calcd for $C_{28}H_{23}PS$: 423.1331, found: 423.1321. Elemental analysis: calcd (%): C: 79.59, H: 5.49, S: 7.59; found: C: 79.24, H: 5.45, S: 8.00.

[9-(S)PPh$_2$-10-Ph-(C$_{14}$H$_8$)] (8)

Yield 0.21 g (79 %). Yellow crystals. ^{1}H NMR (300 MHz, CDCl$_3$): δ [ppm] = 8.12 – 8.09 (m, 2H, $H_{1,8}$), 7.89 – 7.81 (m, 4H, *o*-Ph), 7.67 – 7.57 (m, 5H, $H_{4,5,12,14,16}$), 7.47 – 7.44 (m, 2H, $H_{13,15}$), 7.37 – 7.28 (m, 6H, *p*-Ph, *m*-Ph), 7.23 – 7.18 (m, 2H, $H_{3,6}$), 7.06 – 7.01 (m, 2H, $H_{2,7}$). ^{13}C{^{1}H} NMR (75 MHz, CDCl$_3$): δ [ppm] = 143.9 (s, 1C, C_{10}),143.8 (s, 1C, C_{11}), 138.3 (s, 2C, $C_{8a,9a}$), 137.3 (d, $^1J_{CP}$ = 82.7 Hz, 2C, *i*-Ph), 133.0 (d, $^3J_{CP}$ = 7.7 Hz, 2C, $C_{4a,10a}$), 130.9 (d, $^2J_{CP}$ = 10.4 Hz, 4C, *o*-Ph), 130.8 (s, 2C, $C_{13,15}$), 130.6 (d, $^4J_{CP}$ = 3.1 Hz, 2C, *p*-Ph) 128.5 (d, $^3J_{CP}$ = 12.9 Hz, 4C, *m*-Ph), 128.0 (s, 3C, $C_{12,14,16}$), 127.8 (s, 2C, $C_{4,5}$), 127.5 (d, $^2J_{CP}$ = 10.6 Hz, 2C, $C_{1,8}$), 125.3 (s, 2C, $C_{2,7}$), 124.9 (s, 2C, $C_{3,6}$), 122.6 (d, $^1J_{CP}$ = 88.7 Hz, C_9). ^{31}P{^{1}H} NMR (121 MHz, CDCl$_3$): δ [ppm] = 34.55 (s). HRMS (EI): calcd for $C_{32}H_{23}PS$: 470.1258, found: 470.1250. Elemental Analysis: calcd (%): C: 81.68, H: 4.93, S: 6.81; found: C: 80.57, H: 4.90, S: 6.80.

Table S1. ^{31}P-NMR resonances of thiophosphoranyal anthracens **5** - **8** and of literature known thiophosphoranyl aromatic hydrocarbons.

Compound	δ(^{31}P) / ppm
[9-(S)PPh$_2$Anthracenyl] (**5**)	34.2 in $CDCl_3$; 34.37[3] in C_6D_6
[9-(S)PPh$_2$-10-Me-Anthracenyl] (**6**)	34.4 in $CDCl_3$
[9-(S)PPh$_2$-10-Et-Anthracenyl] (**7**)	34.5 in $CDCl_3$
[9-(S)PPh$_2$-10-Ph-Anthracenyl] (**8**)	34.5 in $CDCl_3$
(S)PPh$_3$	43.4[1] in $CDCl_3$
[1-(S)PPh$_{2-}$Naphtyl]	42.6[2] in $CDCl_3$
[9-(S)PPh$_2$Phenantryl]	42.85[3] in C_6D_6
[1-(S)PPh$_2$Pyreny]	42.65[3] in C_6D_6

S2. X-Ray Crystallographic Analysis

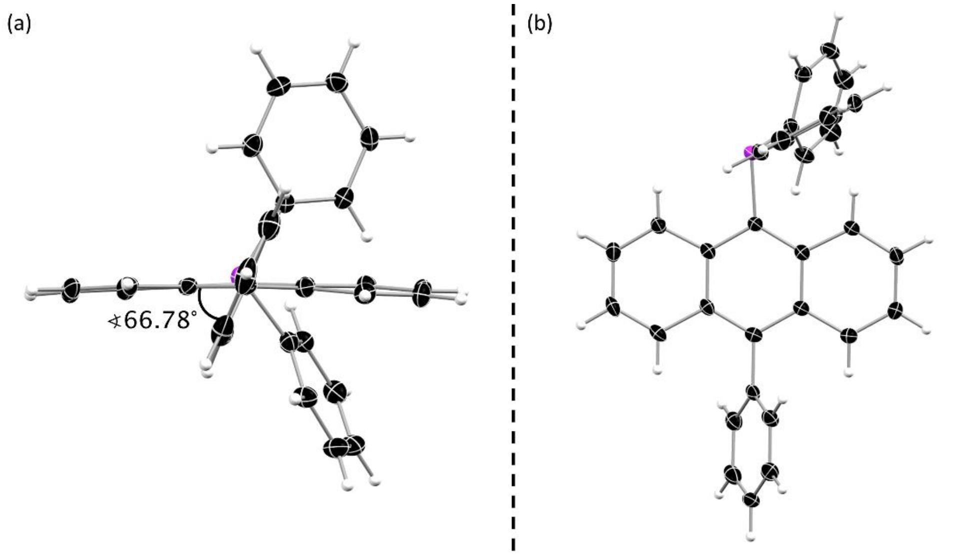

Fig. S1. (a) Side view of the solid-state structure of [9-PPh_2-10-Ph-($C_{14}H_8$)] (**4**) revealing the orientation of the phenyl substituent in 10-position with an intersection angle of 66.78(6)° . (b) The front view shows the shielding of the anthracene core by the two substituents in 9- and 10-position.

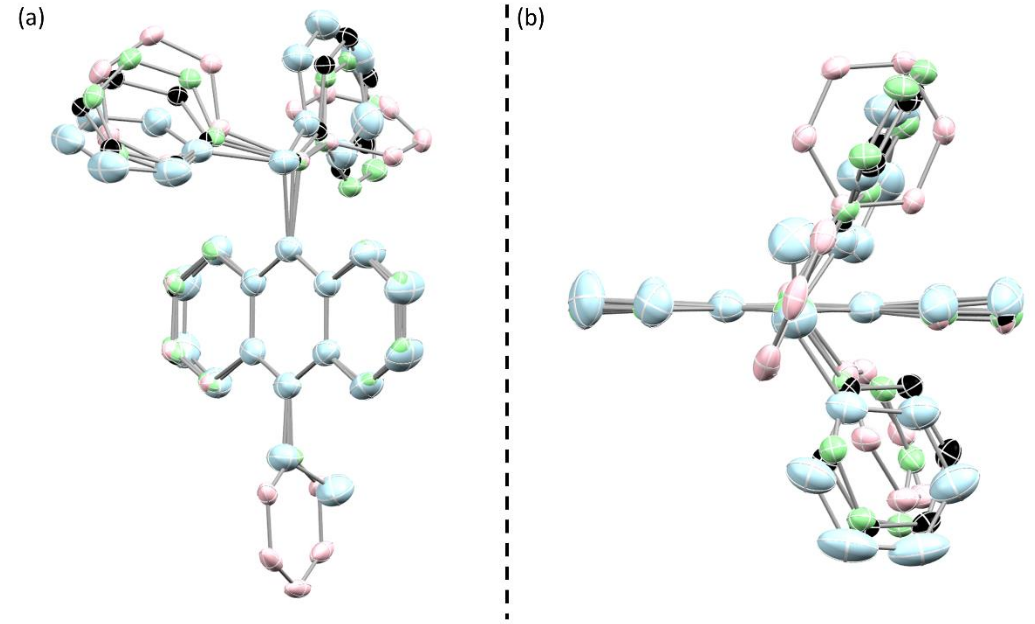

Fig. S2. (a) Front view of the overlay of the molecular structures **1** (black), **2** (green), **3** (blue) and **4** (red) and (b) side view. Co-crystallised solvent and hydrogen atoms are omitted for clarity.

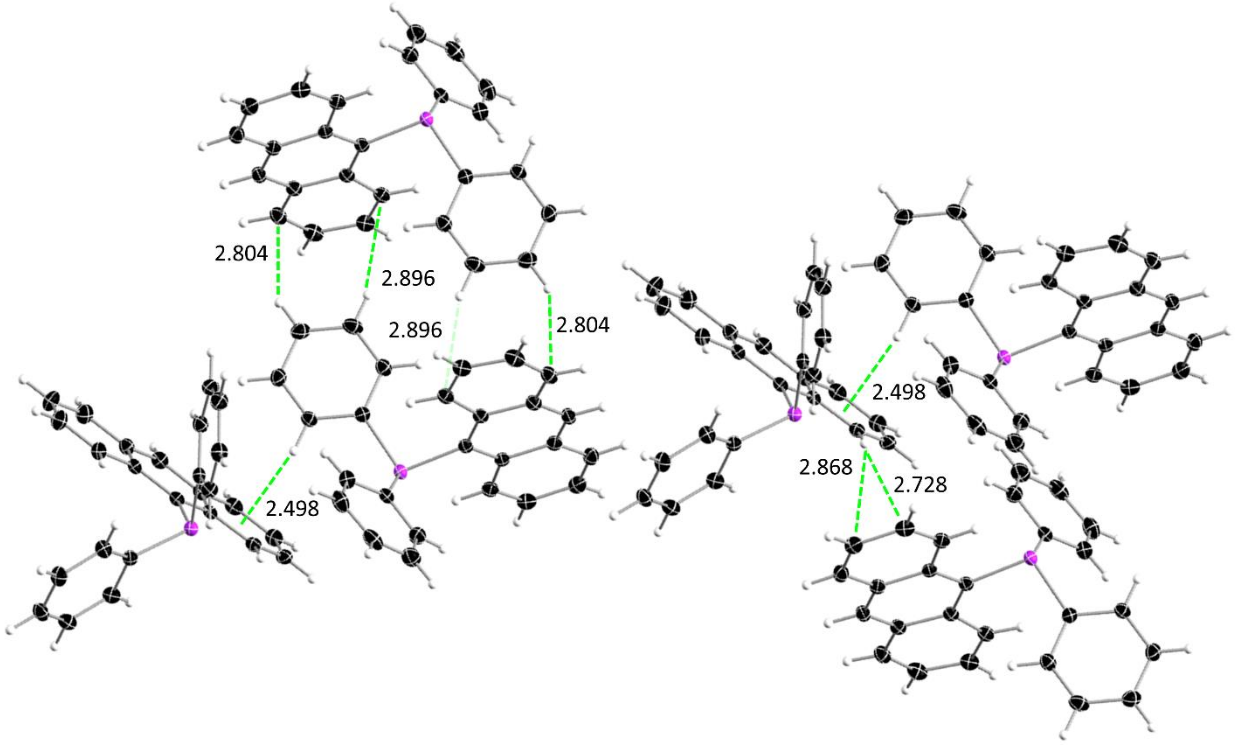

Fig. S3. Shortest C–H···π distances (in Å) as found in the solid-state structure of **1.**

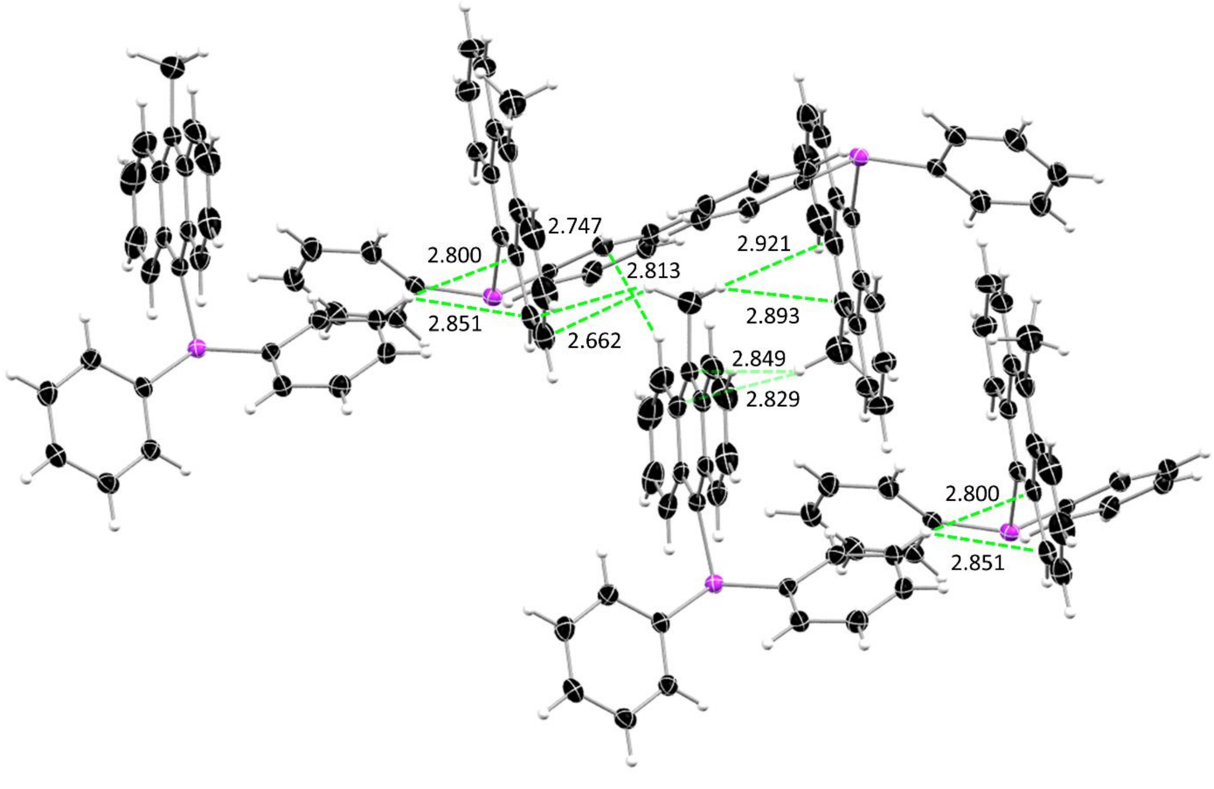

Fig. S4. Shortest C–H···π distances (in Å) as found in the solid-state structure of **2.** Disordered hydrogens of the methyl-group are omitted for clarity.

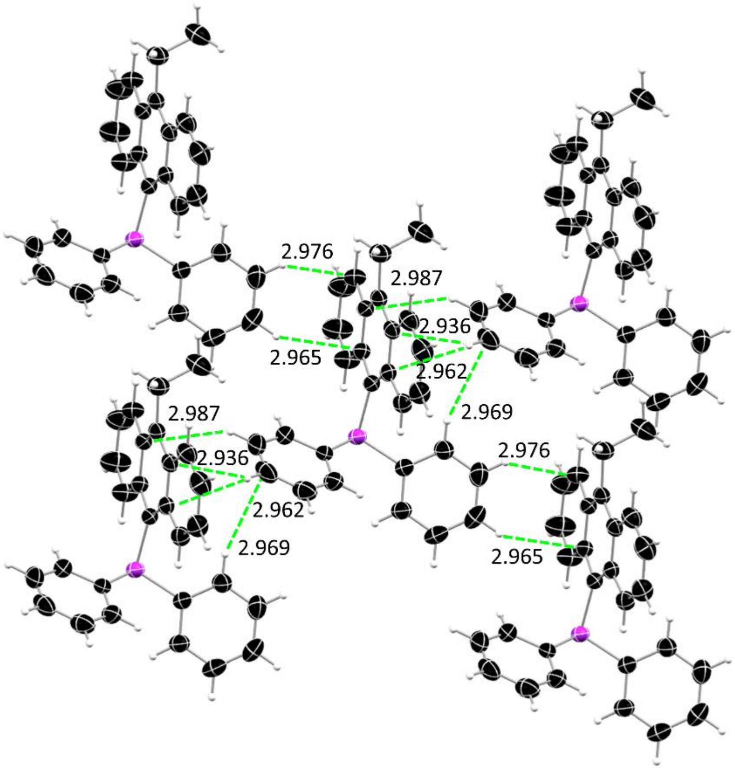

Fig. S5. Shortest C–H···π distances (in Å) as found in the solid-state structure of **3**.

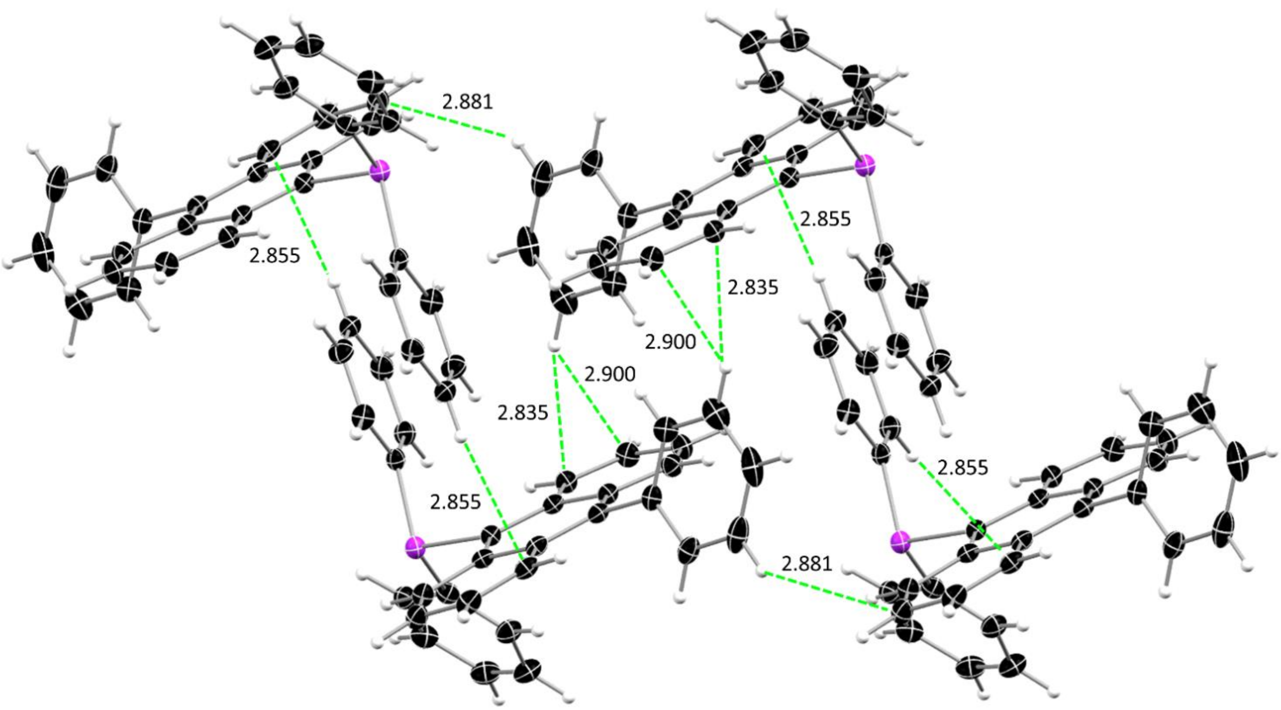

Fig. S6. Shortest C–H···π distances (in Å) as found in the solid-state structure of **4**. Co-crystallised toluene is omitted for clarity.

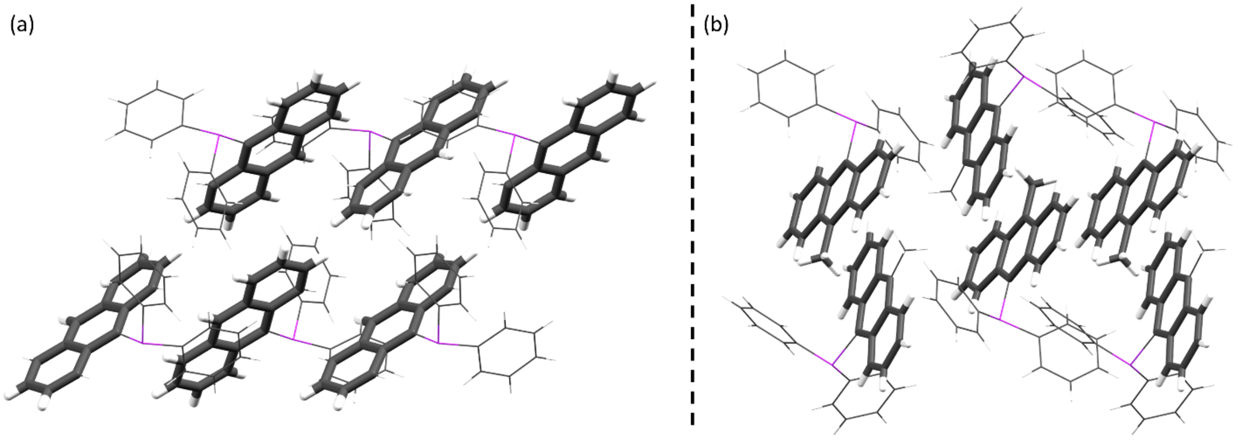

Fig. S7. Crystal packing of **1** (a) and **2** (b) showing the orientation of the anthracene moieties (bold).

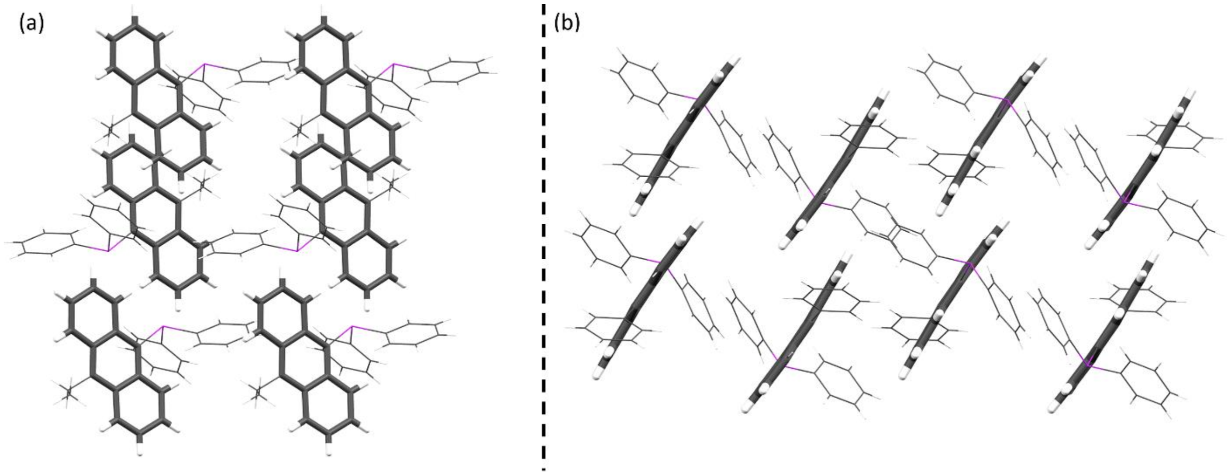

Fig. S8. Crystal packing of **3** (a) and **4** (b) showing the orientation of the anthracene moieties (bold).

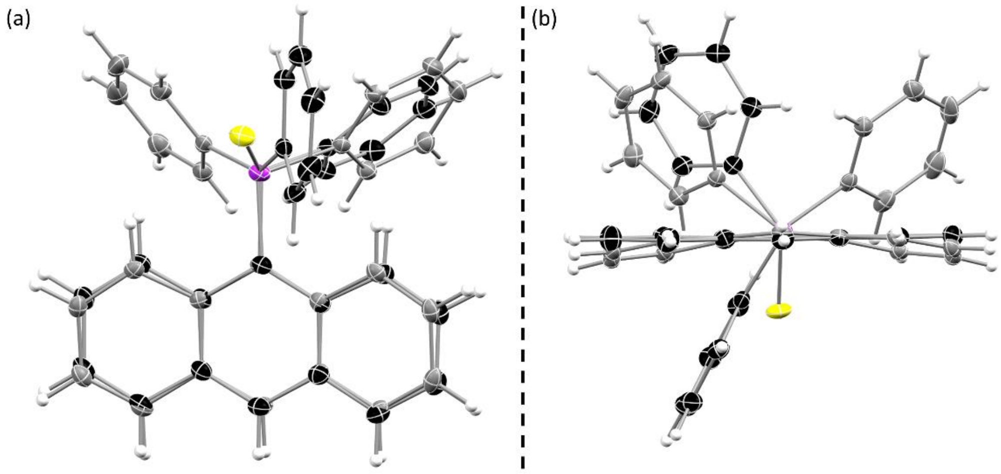

Fig. S9. (a) Front view and (b) side view of the structural overlay of **1** (black) and **5** (grey) revealing the changes of the molecular structure upon oxidation. Only one molecule of the asymmetric unit is shown.

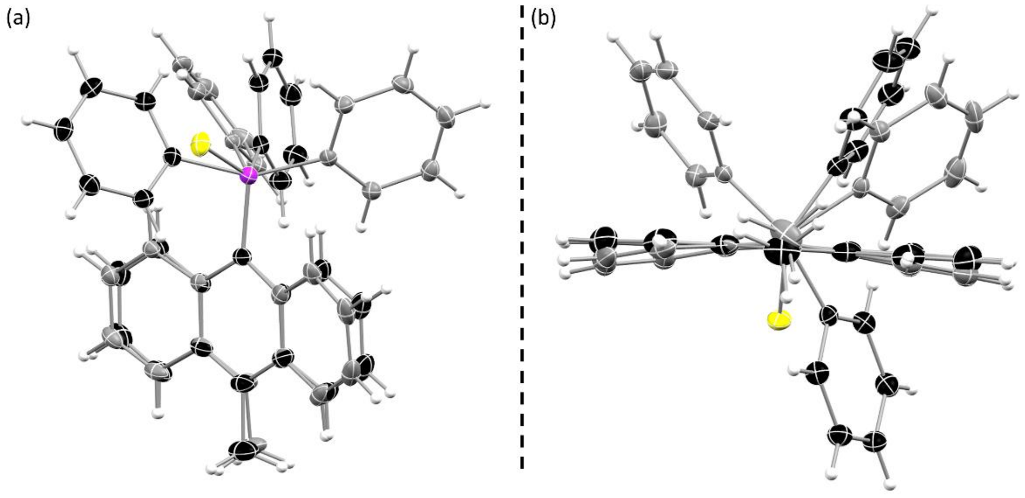

Fig. S10. (a) Front view and (b) side view of the structural overlay of **2** (black) and **6** (grey) revealing the changes of the molecular structure upon oxidation. Disordered hydrogen atoms at the methyl groups are omitted for clarity. Only one molecule of the asymmetric unit is shown.

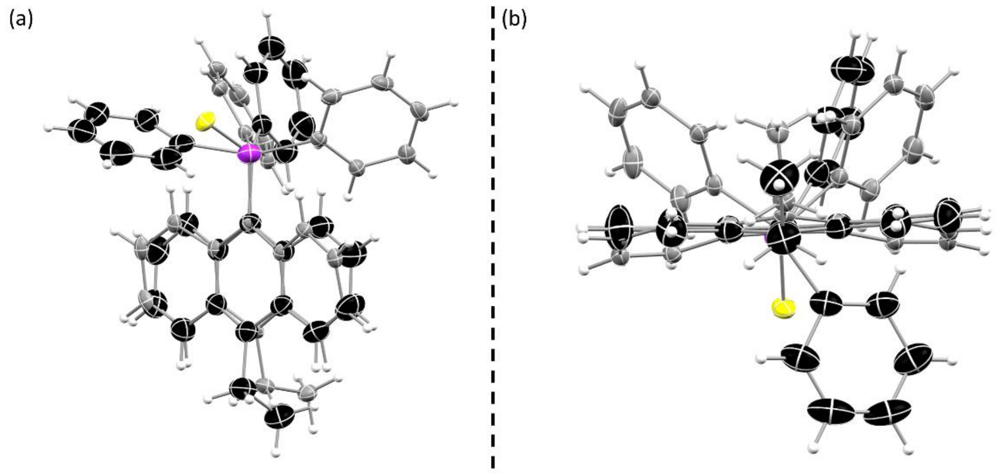

Fig. S11. (a) Front view and (b) side view of the structural overlay of **3** (black) and **7** (grey) revealing the changes of the molecular structure upon oxidation. For **7** Only one molecule of the asymmetric unit is shown.

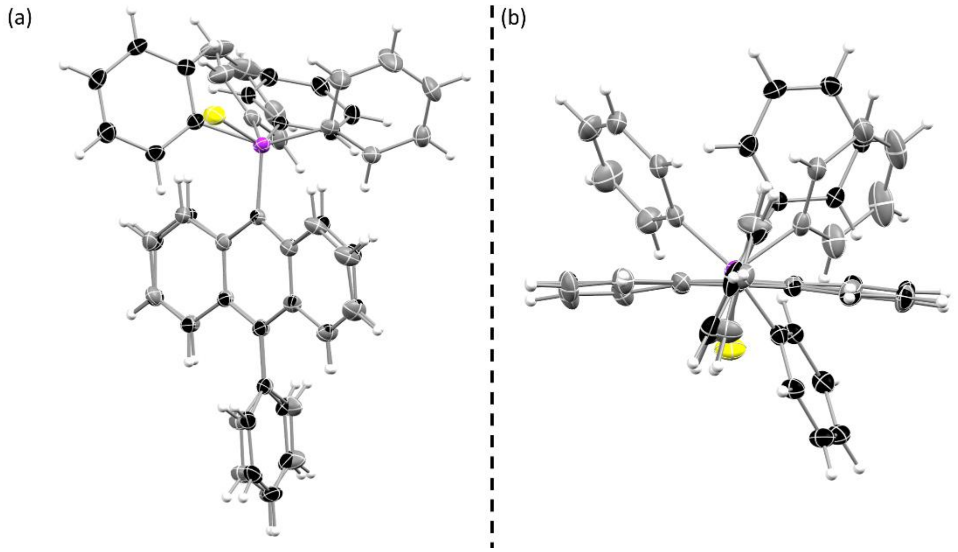

Fig. S12. (a) Front view and (b) side view of the structural overlay of **4** (black) and **8** (grey) revealing the changes of the molecular structure upon oxidation. Co-crystallised solvent in the structure of **4** is omitted for clarity.

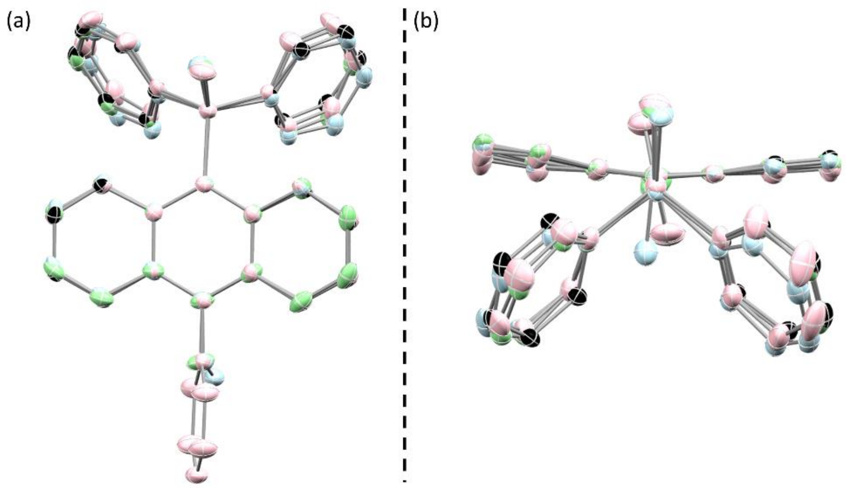

Fig. S13. (a) Front view of the overlay of the molecular structures **5** (black), **6** (green), **7** (blue) and **8** (red) and (b) side view. Hydrogen atoms are omitted for clarity.

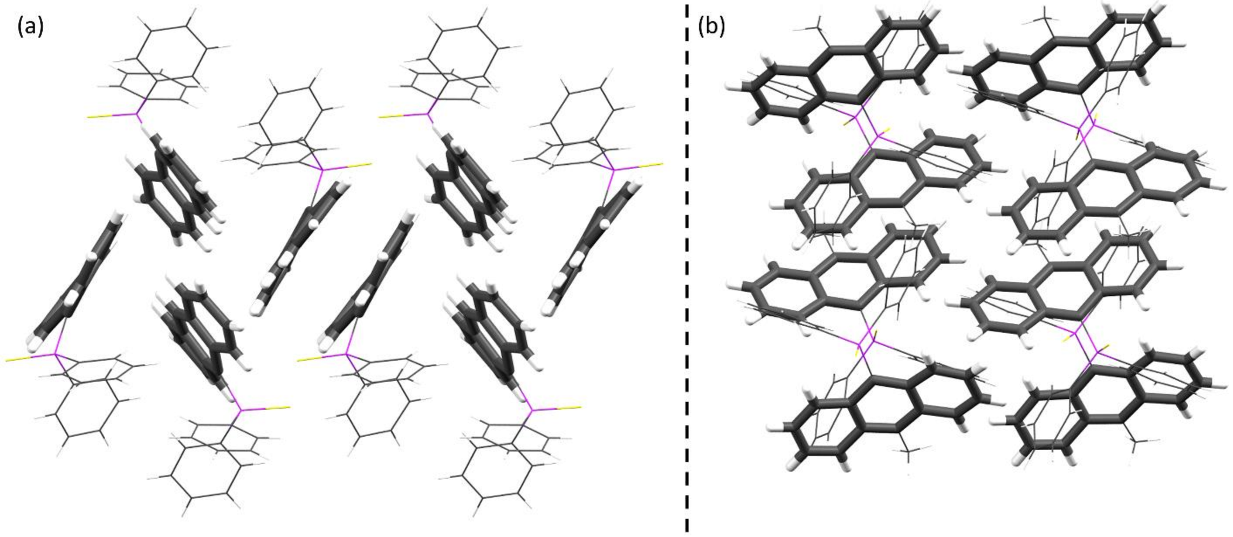

Fig. S14. Crystal packing of **5** (a) and **6** (b) showing the orientation of the anthracene moieties (bold).

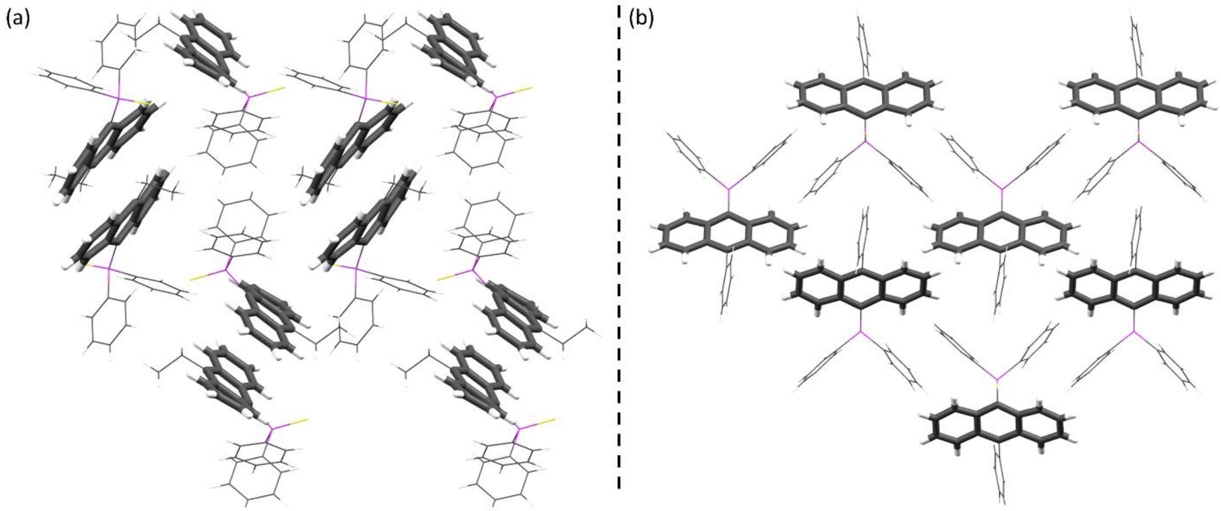

Fig. S15. Crystal packing of **7** (a) and **8**(b) showing the orientation of the anthracene moieties (bold).

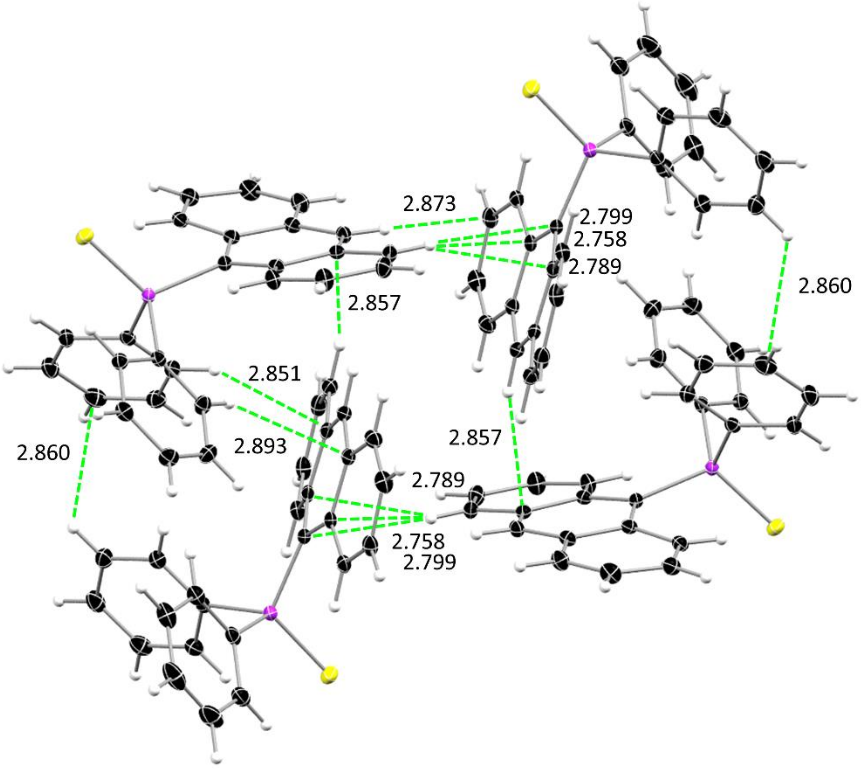

Fig. S16. Shortest C–H···π distances as found in the solid-state structure of **5**.

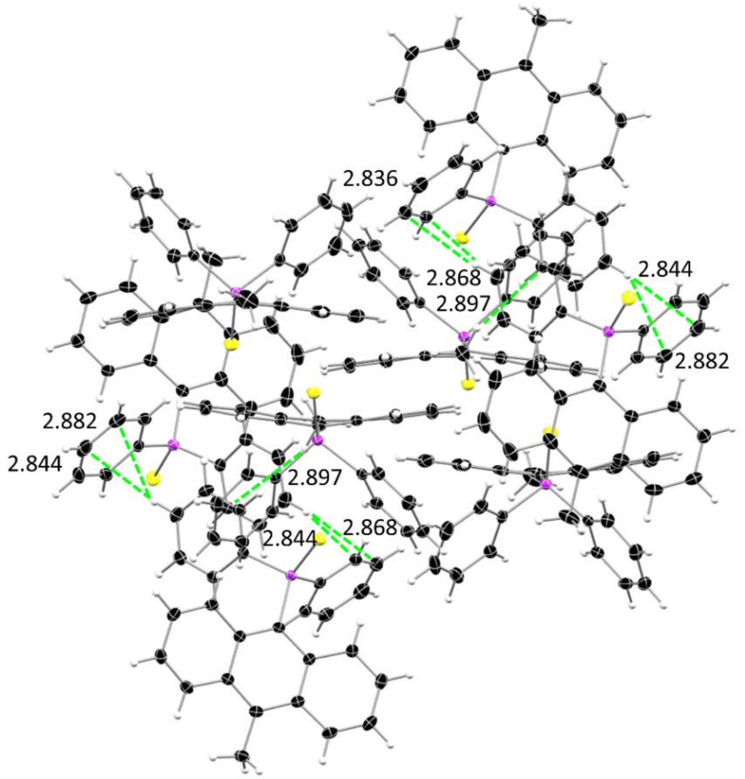

Fig. S17. Shortest C–H···π distances as found in the solid-state structure of **6**. Disordered hydrogens of the methyl group are omitted for clarity.

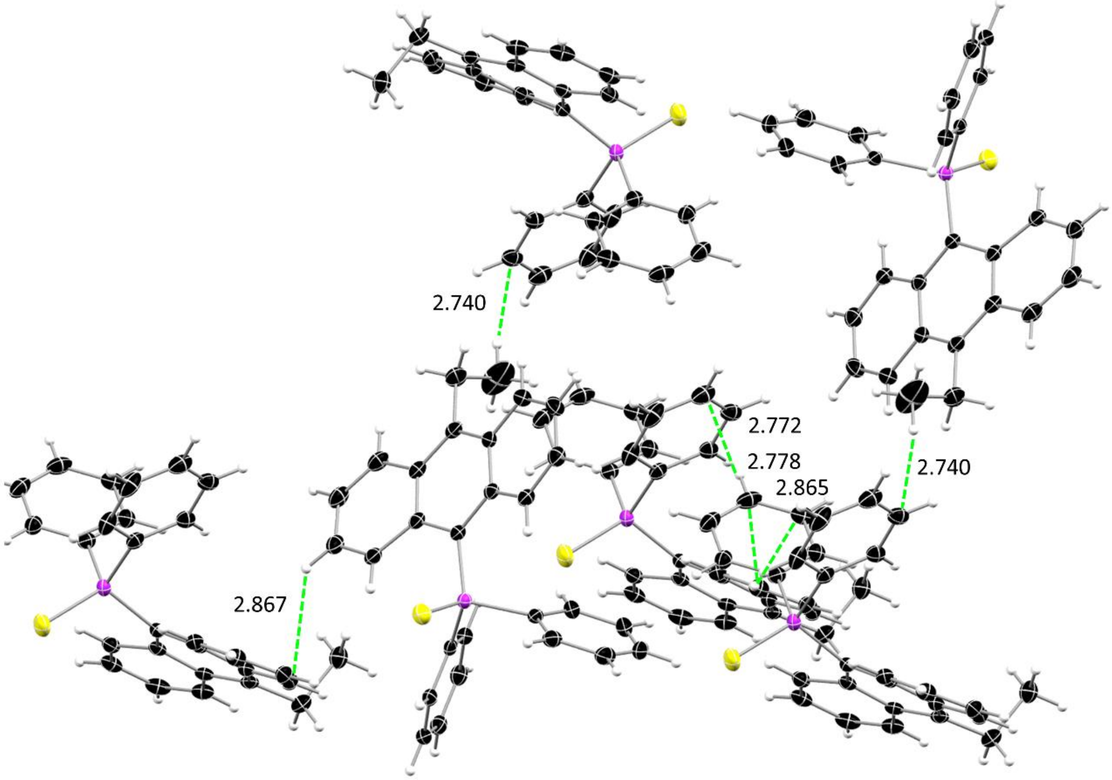

Fig. S18. Shortest C–H···π distances as found in the solid-state structure of **7**.

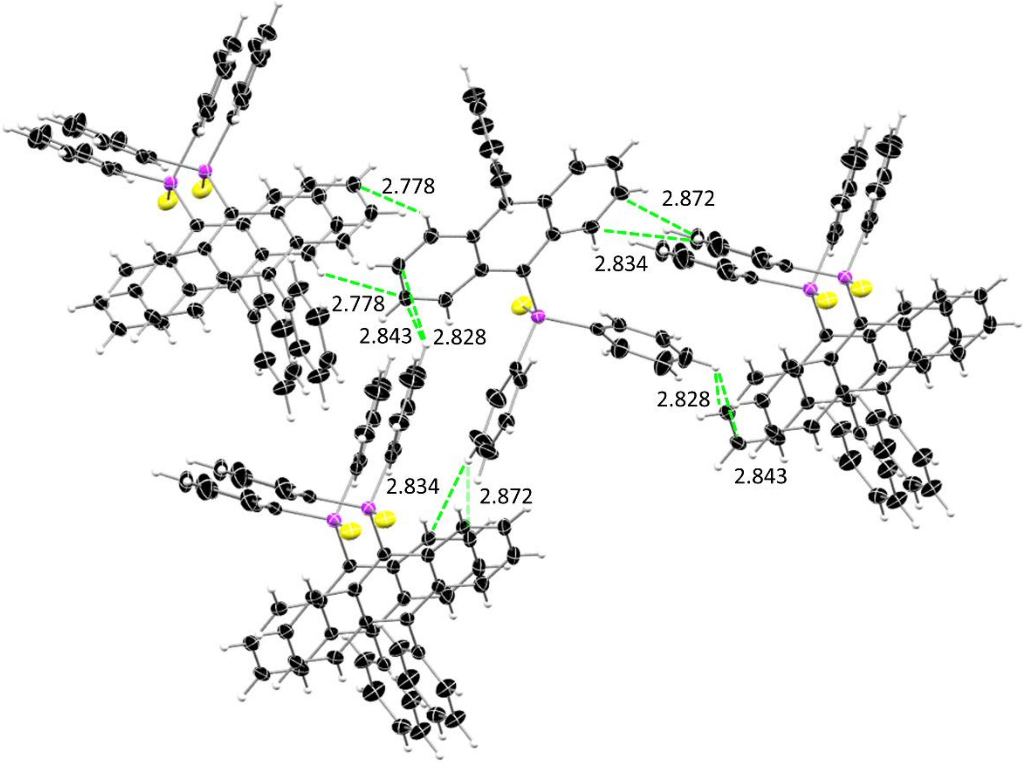

Fig. S19. Shortest C–H···π distances as found in the solid-state structure of **8**.

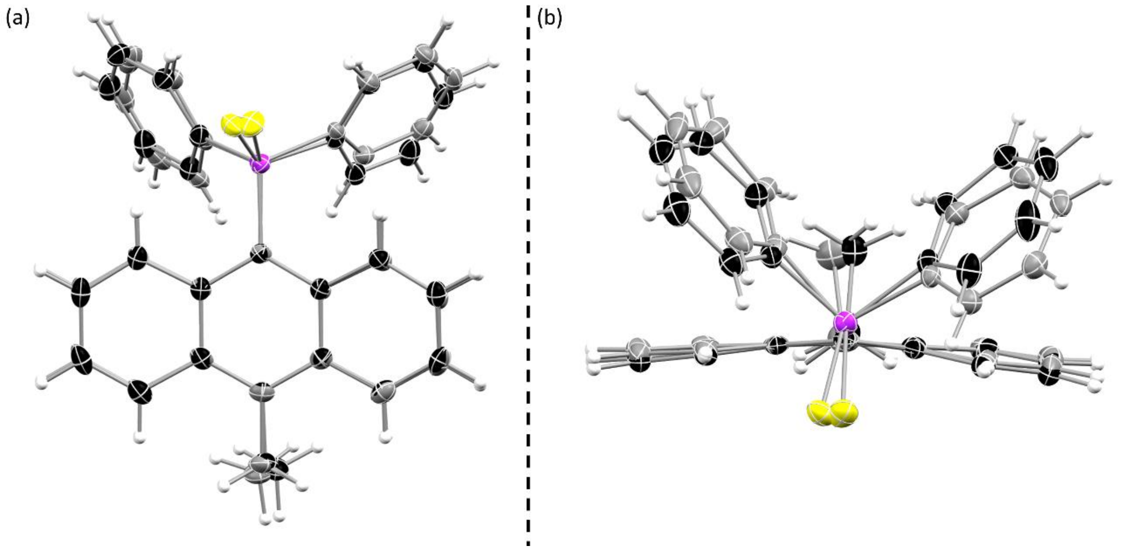

Fig. S20. (a) Front view and (b) side view of the structural overlay of the anthracene moieties of **7** (black) and **7a**. The co-crystallised solvent is omitted for clarity.

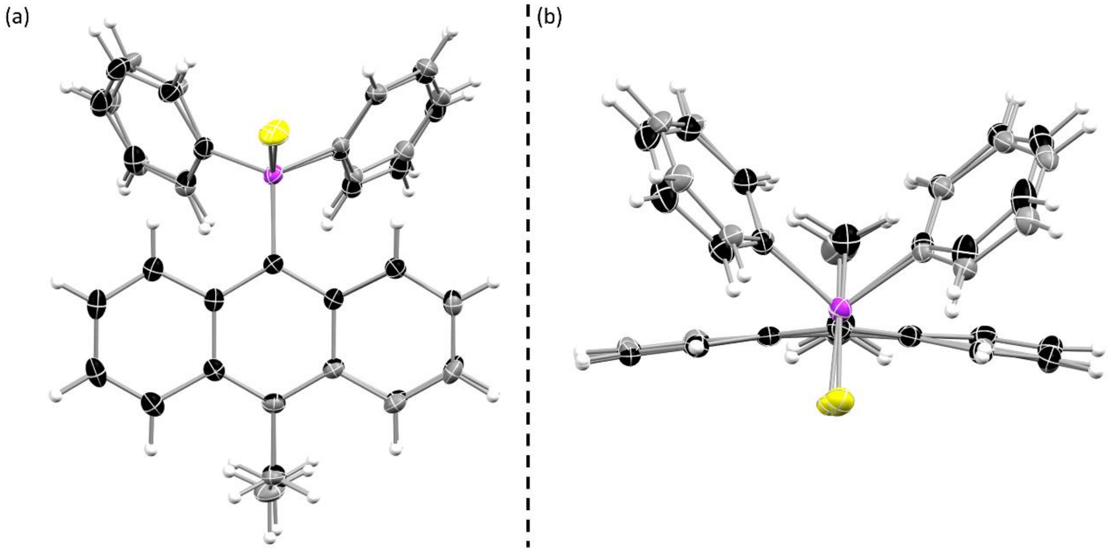

Fig. S21. (a) Front view and (b) side view of the structural overlay of the anthracene moieties of **7** (black) and **7d**. The co-crystallised solvent is omitted for clarity.

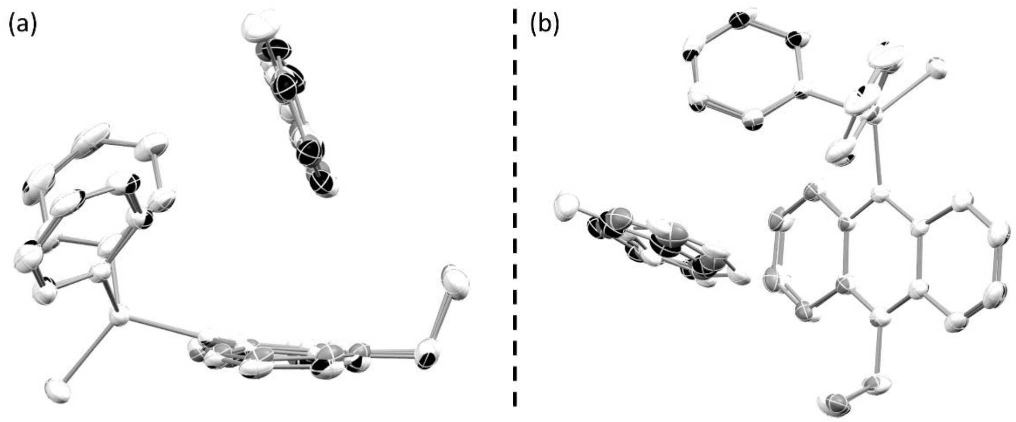

Fig. S22. (a) Side view of the overlay of the molecular structures **7a** (black), **7b** (grey) and **7c** (white) and b) side view. Hydrogen atoms are omitted for clarity.

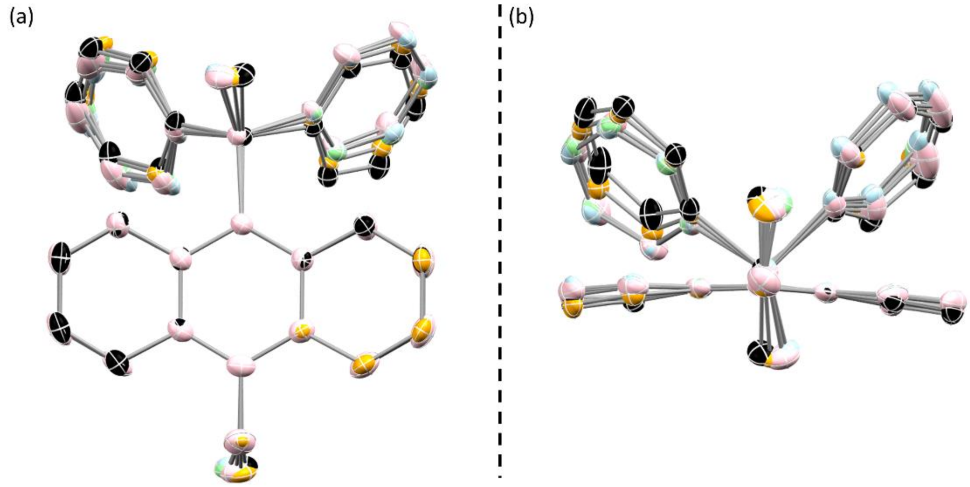

Fig. S23. (a) Front view of the overlay of the anthracene moieties of **7** (black) and its co-crystals **7a** (green), **7b** (blue), **7c** (red) and **7d** (organge) and (b) side view.

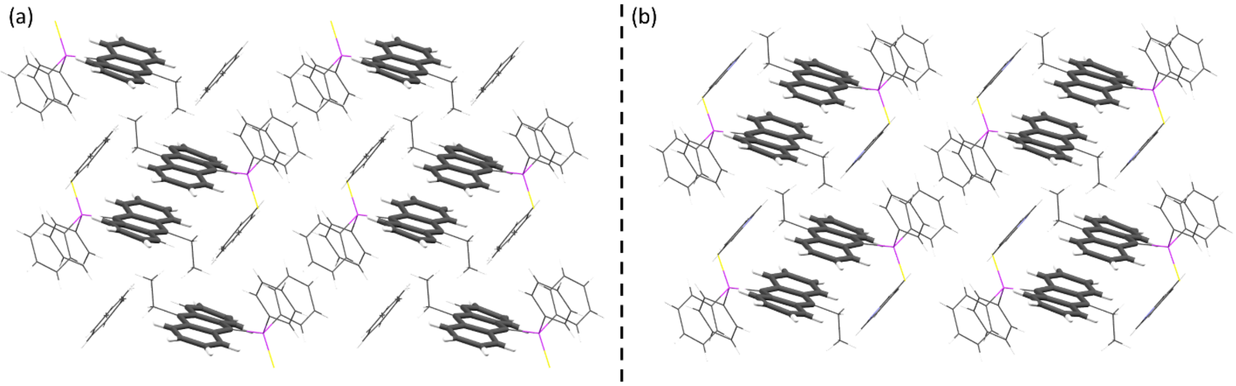

Fig. S24. Crystal packing of **7a** (a) and **7b** (b) showing the orientation of the anthracene moieties (bold).

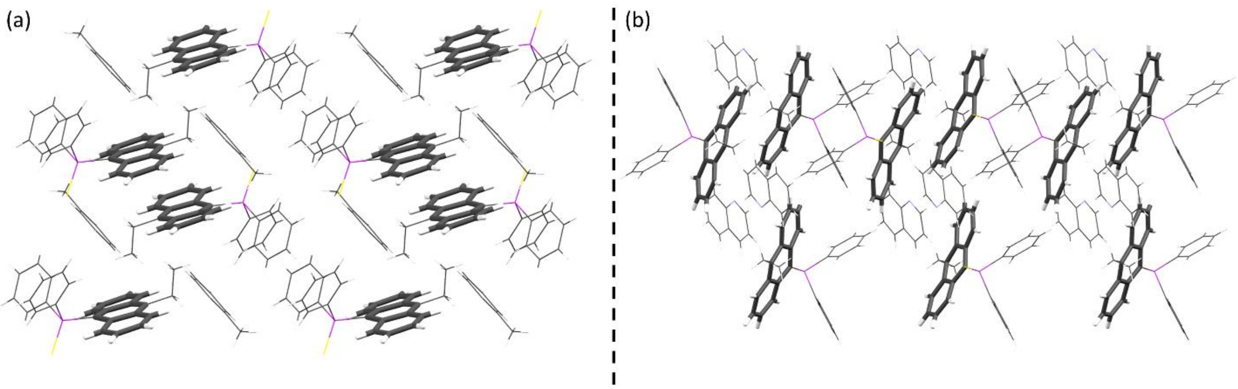

Fig. S 25. Crystal packing of **7c** (a) and **7d** (b) showing the orientation of the anthracene moieties (bold).

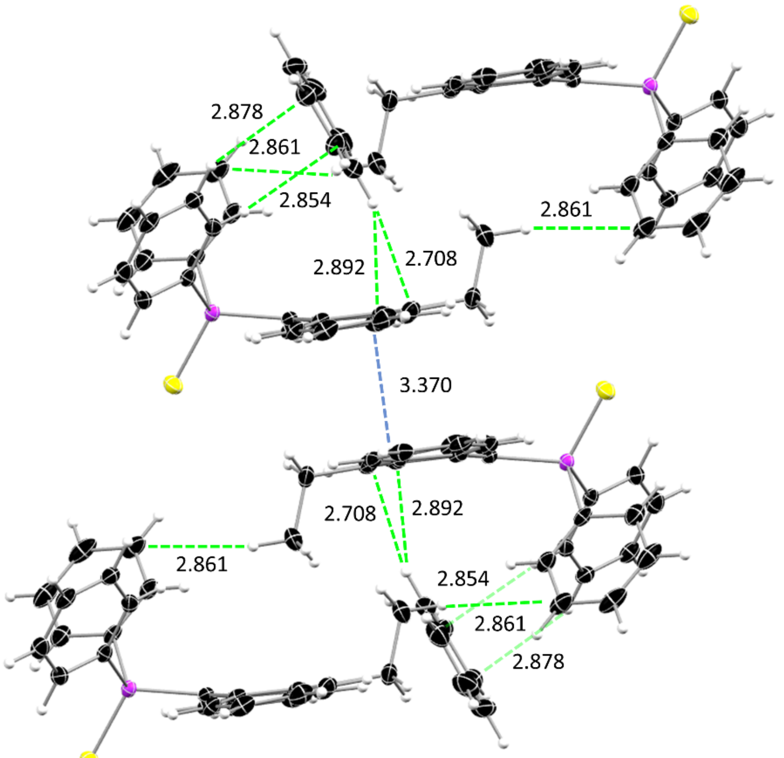

Fig. S26. Shortest C–H···π distances as found in the solid-state structure of **7a**.

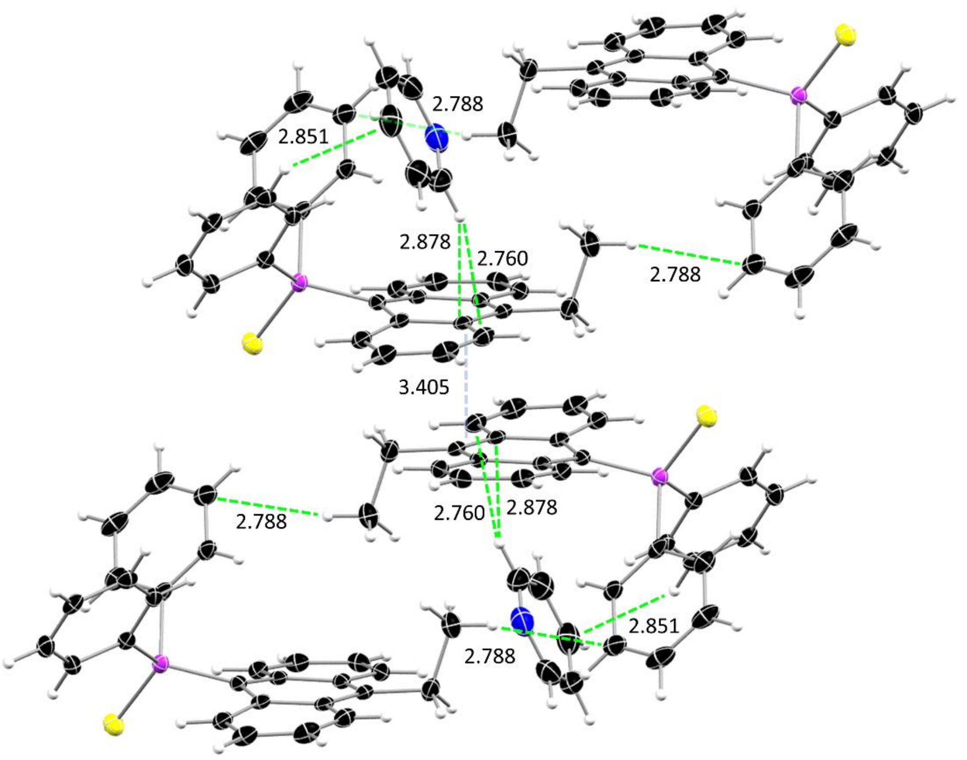

Fig. S27. Shortest C–H···π distances as found in the solid-state structure of **7b**.

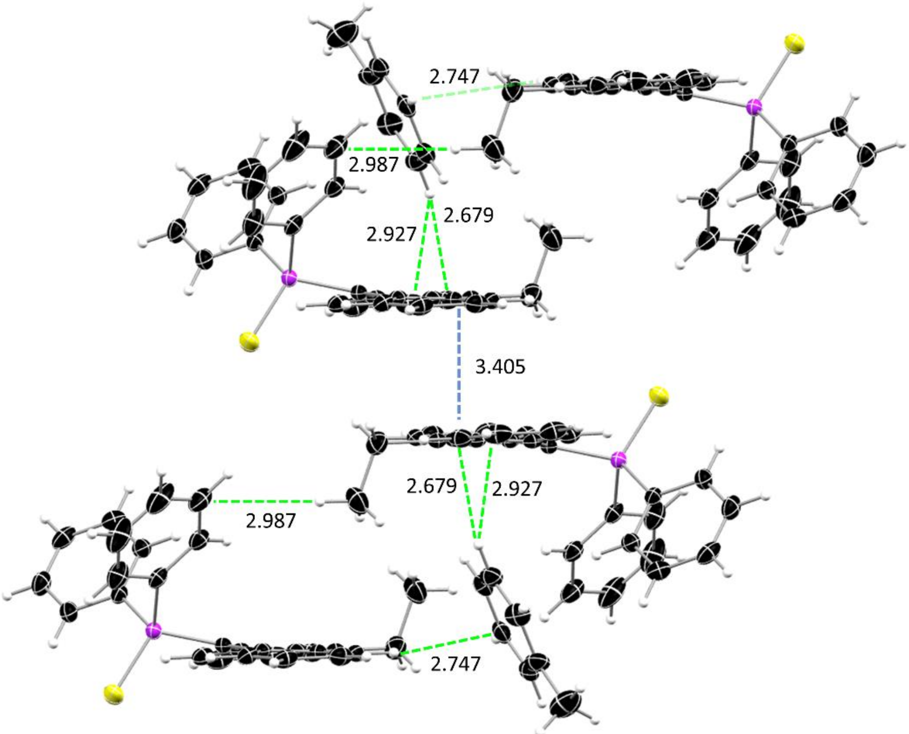

Fig. S28. Shortest C–H···π distances as found in the solid-state structure of **7c**.

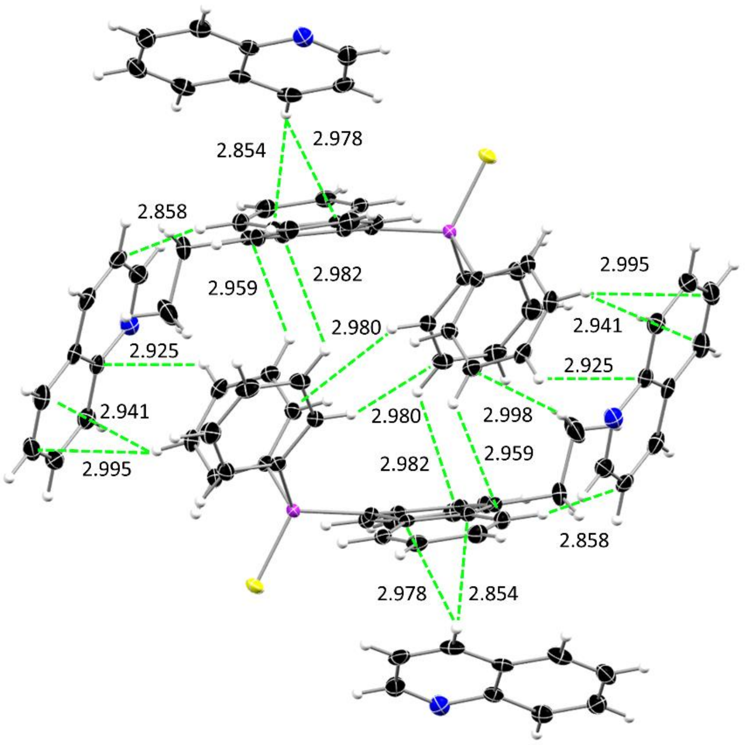

Fig. S29. Shortest C–H···π distances as found in the solid-state structure of **7d**.

Table S2. Crystallographic data of diphenylphosphanyl anthracens **1** – **4**.

Identification Code	**1**	**2**	**3**	**4**
CCDC	1991516	1991517	1991518	1991519
Empirical formula	$C_{26}H_{19}P$	$C_{27}H_{21}P$	$C_{28}H_{23}P$	$C_{32}H_{23}P\cdot½\ C_7H_8$
Formula weight [g/mol]	362.38	376.41	390.43	484.54
Temperature [K]	100(2)	100(2)	100(2)	100(2)
Wavelength [Å]	0.71073	0.71073	0.56086	0.71073
Crystal system	Triclinic	Triclinic	Triclinic	Monoclinic
space group	$P\bar{1}$	$P\bar{1}$	$P\bar{1}$	*C*2/c
a [Å]	10.826(2)	10.899(2)	9.013(2)	15.942(2)
b [Å]	12.274(2)	12.476(2)	9.655(2)	11.129(2)
c [Å]	14.531(3)	14.596(3)	13.134(3)	29.140(4)
α [°]	74.58(4)	93.83(2)	94.87(2)	90
β [°]	88.28(4)	97.89(3)	101.21(2)	102.79(3)
γ [°]	89.36(5)	90.27(2)	108.20(3)	90
Volume [$Å^3$]	1860.5(7)	1961.3(6)	1052.0(4)	5041.7(14)
Z	4	4	2	8
ρ_{calc}	1.294	1.275	1.233	1.277
μ [mm^{-1}]	0.155	0.150	0.082	0.133
F(000)	760	792	412	2040
θ range [°]	1.454 to 26.440	1.412 to 26.381	1.774 to 20.588	1.433 to 26.438
Reflections collected	83493	47776	82609	25605
Independent reflections	7675	8046	4337	5177
R(int)	0.0464	0.0347	0.0347	0.0653
Max. / min. transmission	0.7454 / 0.6943	0.7454 / 0.6942	0.7445 / 0.7252	0.7454 / 0.6671
Restraints / parameter	0 / 487	0 / 509	0 / 263	0 / 332
GooF	1.032	1.029	1.045	1.059
R1 / *w*R2 (I > 2σ(I))	0.0357 / 0.0839	0.0353 / 0.0873	0.0365 / 0.0938	0.0467 / 0.1133
R1 / *w*R2 (all data)	0.0422 / 0.0877	0.0432 / 0.0925	0.0452 / 0.0996	0.0660 / 0.1239
max. diff peak / hole [e $Å^{-3}$]	0.370 to -0.327	0.380 to -0.314	0.248 to -0.205	0.513 to -0.383

Table S3. Crystallographic data of thiophosphoranyl anthracens **5** – **8**.				
Identification Code	**5**	**6**	**7**	**8**
CCDC	1991520	1991521	1991522	1991523
Empirical formula	$C_{26}H_{19}PS$	$C_{27}H_{21}PS$	$C_{28}H_{23}PS$	$C_{32}H_{23}PS$
Formula weight [g/mol]	394.44	408.47	422.49	470.53
Temperature [K]	100(2)	100(2)	100(2)	100(2)
Wavelength [Å]	0.71073	0.71073	0.71073	0.71073
Crystal system	Triclinic	Monoclinic	Triclinic	Orthorhombic
space group	$P\bar{1}$	$P2_1/n$	$P\bar{1}$	$P2_12_12_1$
a [Å]	10.232(2)	17.128(2)	9.791(2)	6.746(2)
b [Å]	12.335(2)	13.796(2)	13.324(2)	16.049(2)
c [Å]	17.378(3)	17.412(3)	17.335(3)	22.369(3)
α [°]	101.61(2)	90	85.56(2)	90
β [°]	91.21(2)	90.070(2)	87.65(3)	90
γ [°]	112.00(3)	90	74.83(2)	90
Volume [Å^3]	1980.7(7)	4114.4(10)	2175.6(7)	2421.8(8)
Z	4	8	4	4
ρ_{calc}	1.323	1.319	1.290	1.291
μ [mm^{-1}]	0.253	0.246	0.235	0.219
F(000)	824	1712	888	984
θ range [°]	1.203 to 27.504	1.667 to 26.399	1.178 to 26.424	1.562 to 26.394
Reflections collected	521789	168507	52615	33453
Independent reflections	9063	8439	8949	4985
R(int)	0.0277	0.0479	0.0346	0.0645
Max. / min. transmission	0.7456 / 0.7041	0.7454 / 0.6960	0.7454 / 0.7168	0.7454 / 0.6168
Restraints / parameter	0 / 505	0 / 526	0 / 543	0 / 308
GooF	1.045	1.023	1.035	1.051
R1 / *w*R2 (I > 2σ(I))	0.0306 / 0.0787	0.0308 / 0.0773	0.0341 / 0.0838	0.0400 / 0.0970
R1 / *w*R2 (all data)	0.0353 / 0.0822	0.0373 / 0.0814	0.0418 / 0.0886	0.0500 / 0.1031
max. diff peak / hole [e Å^{-3}]	0.410 / -0.353	0.347 / -0.30	0.370 / -0.313	0.458 / -0.299

Table S4. Crystallographic data of host-guest co-crystals **7a** – **7d**.				
Identification Code	**7a**	**7b**	**7c**	**7d**
CCDC	1991524	1991525	1991526	1991527
Empirical formula	$C_{34}H_{29}PS$	$C_{33}H_{28}NPS$	$C_{35}H_{31}PS$	$C_{37}H_{30}NPS$
Formula weight [g/mol]	500.60	501.59	514.63	551.65
Temperature [K]	100(2)	100(2)	100(2)	100(2)
Wavelength [Å]	0.71073	0.71073	0.71073	0.71073
Crystal system	Triclinic	Triclinic	Triclinic	Monoclinic
space group	$P\bar{1}$	$P\bar{1}$	$P\bar{1}$	$P2_1/c$
a [Å]	9.218(2)	9.160(4)	9.411(2)	10.068(2)
b [Å]	10.125(2)	10.087(4)	10.333(2)	12.168(2)
c [Å]	15.728(3)	15.699(7)	15.806(3)	23.120(3)
α [°]	72.15(29	72.31(2)	71.39(2)	90
β [°]	78.83(3)	78.83(3)	88.10(3)	93.35(2)
γ [°]	70.10(2)	69.42(2)	68.50(2)	90
Volume [Å^3]	1307.3(5)	1287.6(10)	1349.2(5)	2827.5(8)
Z	2	2	2	4
ρ_{calc}	1.272	1.294	1.267	1.296
μ [mm^{-1}]	0.207	0.211	0.202	0.199
F(000)	528	528	544	1160
θ range [°]	1.367 to 26.375	1.368 to 25.714	2.209 to 26.372	1.765 to 26.405
Reflections collected	31345	30155	26376	66973
Independent reflections	5354	4920	5505	5801
R(int)	0.0526	0.0568	0.0187	0.0460
Max. / min. transmission	0.7454 / 0.6763	0.7453 / 0.6915	0.7454 / 0.7170	0.7454 / 0.6740
Restraints / parameter	390 / 381	0 / 326	450 / 401	0 / 362
GooF	1.028	1.036	1.034	1.023
R1 / *w*R2 (I > 2σ(I))	0.0382 / 0.0808	0.0397 / 0.0881	0.0343 / 0.0867	0.0295 / 0.0762
R1 / *w*R2 (all data)	0.0580 / 0.0888	0.0578 / 0.0966	0.0373 / 0.0889	0.0323 / 0.0785
max. diff peak / hole [e Å^{-3}]	0.380 / -0.290	0.741 / -0.385	0.412 / -0.494	0.347 / -0.377

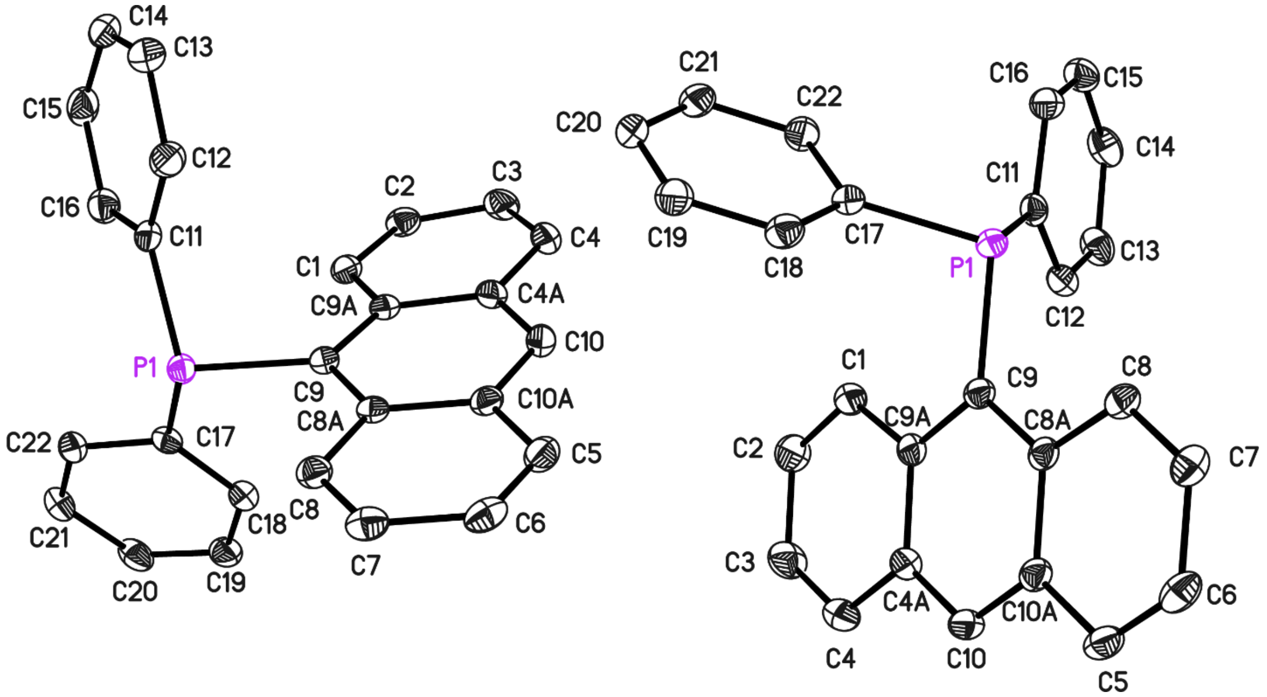

Fig. S30. Asymmetric unit of **1** with anisotropic displacement parameters at 50 % probability level. Hydrogen atoms are omitted for clarity.

Table S5. Bond lengths [Å] and angles [°] for **1**.			
P11-C171	1.8306(17)	C42-C4A2	1.429(2)
P11-C111	1.8412(16)	C52-C62	1.358(2)
P11-C91	1.8513(16)	C52-C10A2	1.433(2)
C11-C21	1.366(2)	C62-C72	1.422(2)
C11-C9A1	1.433(2)	C72-C82	1.364(2)
C21-C31	1.419(2)	C82-C8A2	1.434(2)
C31-C41	1.358(2)	C92-C9A2	1.422(2)
C41-C4A1	1.431(2)	C92-C8A2	1.423(2)
C51-C61	1.355(2)	C102-C10A2	1.390(2)
C51-C10A1	1.431(2)	C102-C4A2	1.396(2)
C61-C71	1.419(2)	C112-C122	1.400(2)
C71-C81	1.367(2)	C112-C162	1.407(2)
C81-C8A1	1.437(2)	C122-C132	1.394(2)
C91-C8A1	1.420(2)	C132-C142	1.388(2)
C91-C9A1	1.421(2)	C142-C152	1.389(2)
C101-C10A1	1.391(2)	C152-C162	1.389(2)
C101-C4A1	1.394(2)	C172-C222	1.400(2)
C111-C161	1.397(2)	C172-C182	1.402(2)

C111-C121	1.401(2)	C182-C192	1.387(2)
C121-C131	1.388(2)	C192-C202	1.389(2)
C131-C141	1.387(3)	C202-C212	1.387(2)
C141-C151	1.385(3)	C212-C222	1.394(2)
C151-C161	1.393(2)	C4A2-C9A2	1.441(2)
C171-C181	1.399(2)	C8A2-C10A2	1.446(2)
C171-C221	1.402(2)		
C181-C191	1.389(2)	C171-P11-C111	106.38(8)
C191-C201	1.386(2)	C171-P11-C91	102.92(7)
C201-C211	1.388(2)	C111-P11-C91	101.74(7)
C211-C221	1.389(2)	C21-C11-C9A1	121.76(15)
C4A1-C9A1	1.443(2)	C11-C21-C31	120.55(15)
C8A1-C10A1	1.445(2)	C41-C31-C21	120.24(15)
P12-C112	1.8302(17)	C31-C41-C4A1	120.88(15)
P12-C172	1.8408(16)	C61-C51-C10A1	121.15(15)
P12-C92	1.8510(17)	C51-C61-C71	119.89(14)
C12-C22	1.362(2)	C81-C71-C61	121.18(15)
C12-C9A2	1.435(2)	C71-C81-C8A1	121.30(15)
C22-C32	1.423(2)	C8A1-C91-C9A1	119.67(13)
C32-C42	1.359(2)	C8A1-C91-P11	116.65(11)
C9A1-C91-P11	123.63(11)	C52-C62-C72	119.75(14)
C10A1-C101-C4A1	121.79(14)	C82-C72-C62	121.11(15)
C161-C111-C121	118.15(14)	C72-C82-C8A2	121.66(14)
C161-C111-P11	126.76(12)	C9A2-C92-C8A2	119.27(14)
C121-C111-P11	115.02(12)	C9A2-C92-P12	124.63(11)
C131-C121-C111	121.17(15)	C8A2-C92-P12	116.05(11)
C141-C131-C121	119.79(16)	C10A2-C102-C4A2	121.71(14)
C151-C141-C131	119.92(16)	C122-C112-C162	118.27(14)
C141-C151-C161	120.26(16)	C122-C112-P12	125.07(12)
C151-C161-C111	120.61(15)	C162-C112-P12	116.28(12)
C181-C171-C221	118.44(14)	C132-C122-C112	120.37(14)
C181-C171-P11	122.24(12)	C142-C132-C122	120.63(15)
C221-C171-P11	118.73(11)	C132-C142-C152	119.59(15)
C191-C181-C171	120.52(14)	C142-C152-C162	120.15(15)
C201-C191-C181	120.37(15)	C152-C162-C112	120.91(14)

C191-C201-C211	119.87(15)	C222-C172-C182	118.33(14)
C201-C211-C221	119.96(15)	C222-C172-P12	125.19(12)
C211-C221-C171	120.80(14)	C182-C172-P12	116.25(11)
C101-C4A1-C41	120.81(14)	C192-C182-C172	120.91(14)
C101-C4A1-C9A1	119.66(14)	C182-C192-C202	120.08(15)
C41-C4A1-C9A1	119.52(14)	C212-C202-C192	119.86(15)
C91-C8A1-C81	123.41(14)	C202-C212-C222	120.21(15)
C91-C8A1-C10A1	119.76(14)	C212-C222-C172	120.57(14)
C81-C8A1-C10A1	116.84(13)	C102-C4A2-C42	120.32(14)
C91-C9A1-C11	123.69(14)	C102-C4A2-C9A2	119.68(14)
C91-C9A1-C4A1	119.47(13)	C42-C4A2-C9A2	120.00(14)
C11-C9A1-C4A1	116.82(14)	C92-C8A2-C82	123.29(14)
C101-C10A1-C51	120.92(14)	C92-C8A2-C10A2	119.97(14)
C101-C10A1-C8A1	119.42(13)	C82-C8A2-C10A2	116.73(13)
C51-C10A1-C8A1	119.63(14)	C92-C9A2-C12	123.41(14)
C112-P12-C172	104.92(7)	C92-C9A2-C4A2	119.87(13)
C112-P12-C92	106.22(7)	C12-C9A2-C4A2	116.72(14)
C172-P12-C92	102.49(7)	C102-C10A2-C52	120.84(14)
C22-C12-C9A2	121.29(15)	C102-C10A2-C8A2	119.48(13)
C12-C22-C32	121.60(15)	C52-C10A2-C8A2	119.68(14)
C42-C32-C22	119.26(15)		
C32-C42-C4A2	121.13(15)		
C62-C52-C10A2	121.07(15)		

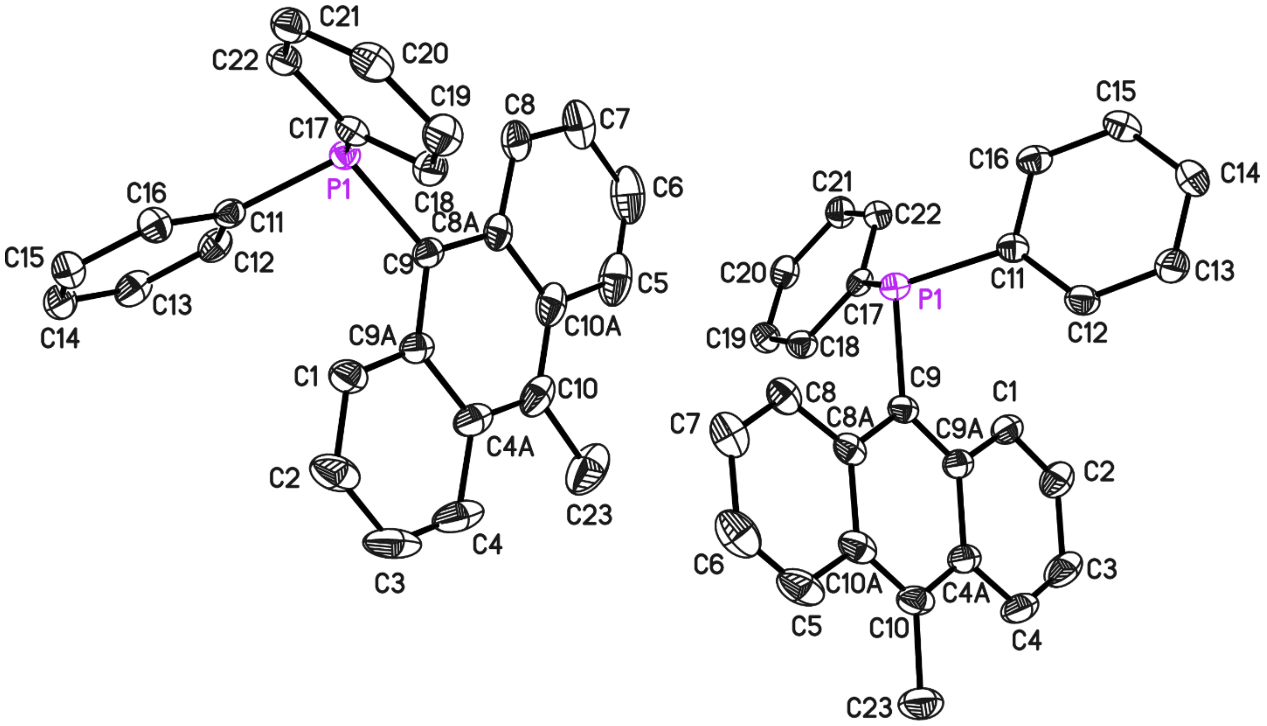

Fig. S31. Asymmetric unit of **2** with anisotropic displacement parameters at 50 % probability level. Hydrogen atoms are omitted for clarity. Hydrogen atoms attached to each C23 are disordered over two positions. The occupancies of the main positions refined to 0.70(3) and 0.75(3) respectively.

Table S6. Bond lengths [Å] and angles [°] for **2.**			
P11-C111	1.8393(15)	C32-C42	1.347(3)
P11-C171	1.8448(14)	C42-C4A2	1.438(2)
P11-C91	1.8488(14)	C52-C62	1.351(3)
C11-C21	1.360(2)	C52-C10A2	1.439(2)
C11-C9A1	1.430(2)	C62-C72	1.415(3)
C21-C31	1.418(2)	C72-C82	1.362(2)
C31-C41	1.352(3)	C82-C8A2	1.435(2)
C41-C4A1	1.436(2)	C92-C8A2	1.414(2)
C51-C61	1.356(3)	C92-C9A2	1.419(2)
C51-C10A1	1.439(2)	C102-C10A2	1.403(2)
C61-C71	1.412(3)	C102-C4A2	1.409(3)
C71-C81	1.358(2)	C102-C232	1.518(2)
C81-C8A1	1.437(2)	C112-C162	1.392(2)
C91-C8A1	1.414(2)	C112-C122	1.4022(19)
C91-C9A1	1.419(2)	C122-C132	1.386(2)
C101-C10A1	1.409(2)	C132-C142	1.384(2)
C101-C4A1	1.410(2)	C142-C152	1.386(2)

C101-C231	1.515(2)	C152-C162	1.390(2)
C111-C121	1.399(2)	C172-C182	1.3966(19)
C111-C161	1.402(2)	C172-C222	1.405(2)
C121-C131	1.388(2)	C182-C192	1.392(2)
C131-C141	1.385(2)	C192-C202	1.387(2)
C141-C151	1.387(2)	C202-C212	1.391(2)
C151-C161	1.386(2)	C212-C222	1.385(2)
C171-C221	1.397(2)	C4A2-C9A2	1.445(2)
C171-C181	1.400(2)	C8A2-C10A2	1.447(2)
C181-C191	1.391(2)		
C191-C201	1.385(2)	C111-P11-C171	109.21(6)
C201-C211	1.388(2)	C111-P11-C91	103.48(6)
C211-C221	1.392(2)	C171-P11-C91	101.26(7)
C4A1-C9A1	1.4473(19)	C21-C11-C9A1	122.25(14)
C8A1-C10A1	1.446(2)	C11-C21-C31	119.98(16)
P12-C172	1.8289(15)	C41-C31-C21	120.18(15)
P12-C112	1.8369(15)	C31-C41-C4A1	122.27(14)
P12-C92	1.8471(15)	C61-C51-C10A1	122.23(16)
C12-C22	1.366(2)	C51-C61-C71	120.39(15)
C12-C9A2	1.432(2)	C81-C71-C61	119.91(16)
C22-C32	1.413(3)	C71-C81-C8A1	122.38(15)
C8A1-C91-C9A1	119.47(13)	C12-C22-C32	120.16(18)
C8A1-C91-P11	116.69(11)	C42-C32-C22	120.45(16)
C9A1-C91-P11	123.83(11)	C32-C42-C4A2	122.11(16)
C10A1-C101-C4A1	120.06(13)	C62-C52-C10A2	122.35(17)
C10A1-C101-C231	119.41(15)	C52-C62-C72	120.02(16)
C4A1-C101-C231	120.52(15)	C82-C72-C62	120.33(17)
C121-C111-C161	117.61(13)	C72-C82-C8A2	122.07(16)
C121-C111-P11	121.73(11)	C8A2-C92-C9A2	119.25(13)
C161-C111-P11	120.02(11)	C8A2-C92-P12	116.58(10)
C131-C121-C111	121.10(14)	C9A2-C92-P12	123.76(11)
C141-C131-C121	120.38(14)	C10A2-C102-C4A2	119.62(14)
C131-C141-C151	119.34(14)	C10A2-C102-C232	120.52(17)
C161-C151-C141	120.41(14)	C4A2-C102-C232	119.86(17)
C151-C161-C111	121.04(13)	C162-C112-C122	118.35(13)

C221-C171-C181	117.72(13)	C162-C112-P12	125.12(11)
C221-C171-P11	124.30(11)	C52-C10A2-C8A2	117.75(16)
C181-C171-P11	117.23(11)	C122-C112-P12	116.50(11)
C191-C181-C171	121.34(13)	C132-C122-C112	120.68(14)
C201-C191-C181	120.14(13)	C142-C132-C122	120.39(14)
C191-C201-C211	119.38(13)	C132-C142-C152	119.45(14)
C201-C211-C221	120.51(14)	C142-C152-C162	120.44(14)
C211-C221-C171	120.91(13)	C152-C162-C112	120.66(13)
C101-C4A1-C41	122.23(14)	C182-C172-C222	118.46(13)
C101-C4A1-C9A1	120.14(14)	C182-C172-P12	123.38(11)
C41-C4A1-C9A1	117.63(14)	C222-C172-P12	117.62(10)
C91-C8A1-C81	122.09(13)	C192-C182-C172	120.53(13)
C91-C8A1-C10A1	120.30(13)	C202-C192-C182	120.44(14)
C81-C8A1-C10A1	117.61(13)	C192-C202-C212	119.55(14)
C91-C9A1-C11	122.34(13)	C222-C212-C202	120.27(14)
C91-C9A1-C4A1	119.96(13)	C212-C222-C172	120.72(13)
C11-C9A1-C4A1	117.70(13)	C102-C4A2-C42	121.80(15)
C101-C10A1-C51	122.58(14)	C102-C4A2-C9A2	120.55(14)
C101-C10A1-C8A1	119.97(14)	C42-C4A2-C9A2	117.65(16)
C51-C10A1-C8A1	117.44(15)	C92-C8A2-C82	122.34(13)
C172-P12-C112	105.27(7)	C92-C8A2-C10A2	120.19(14)
C172-P12-C92	106.46(7)	C82-C8A2-C10A2	117.47(14)
C112-P12-C92	101.52(6)	C92-C9A2-C12	122.26(13)
C22-C12-C9A2	121.67(16)	C92-C9A2-C4A2	119.83(14)
C102-C10A2-C52	121.92(15)	C12-C9A2-C4A2	117.91(14)
C102-C10A2-C8A2	120.33(14)		

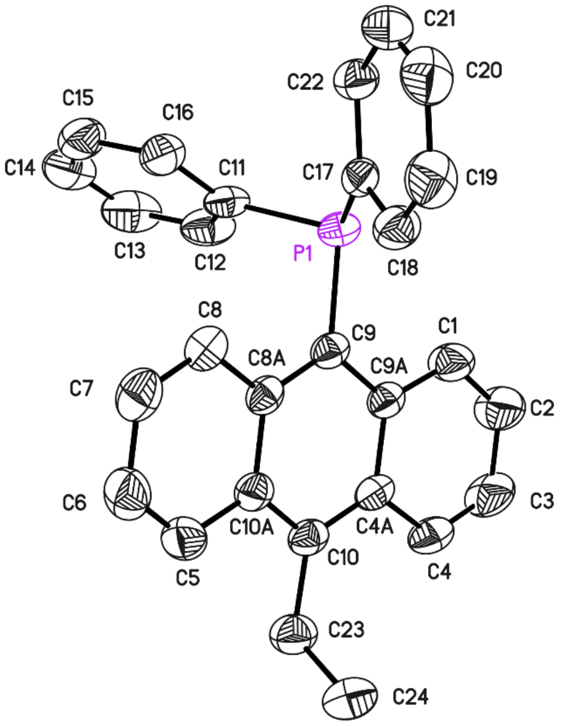

Fig. S32. Asymmetric unit of **3** with anisotropic displacement parameters at 50 % probability level. Hydrogen atoms are omitted for clarity.

Table S7. Bond lengths [Å] and angles [°] for **3**.			
P(1)-C(17)	1.8244(15)	C(1)-C(2)-C(3)	120.36(15)
P(1)-C(11)	1.8383(16)	C(4)-C(3)-C(2)	120.33(15)
P(1)-C(9)	1.8527(15)	C(3)-C(4)-C(4A)	121.73(15)
C(1)-C(2)	1.363(2)	C(10)-C(4A)-C(4)	121.53(14)
C(1)-C(9A)	1.428(2)	C(10)-C(4A)-C(9A)	120.30(13)
C(2)-C(3)	1.410(2)	C(4)-C(4A)-C(9A)	118.17(14)
C(3)-C(4)	1.350(2)	C(6)-C(5)-C(10A)	122.32(15)
C(4)-C(4A)	1.434(2)	C(5)-C(6)-C(7)	119.98(16)
C(4A)-C(10)	1.411(2)	C(8)-C(7)-C(6)	120.61(17)
C(4A)-C(9A)	1.441(2)	C(7)-C(8)-C(8A)	121.99(16)
C(5)-C(6)	1.346(2)	C(9)-C(8A)-C(8)	122.67(13)
C(5)-C(10A)	1.437(2)	C(9)-C(8A)-C(10A)	119.86(13)
C(6)-C(7)	1.406(3)	C(8)-C(8A)-C(10A)	117.47(13)
C(7)-C(8)	1.356(2)	C(9A)-C(9)-C(8A)	119.18(12)
C(8)-C(8A)	1.429(2)	C(9A)-C(9)-P(1)	124.61(11)
C(8A)-C(9)	1.419(2)	C(8A)-C(9)-P(1)	115.99(10)
C(8A)-C(10A)	1.4434(19)	C(9)-C(9A)-C(1)	122.19(13)

C(9)-C(9A)	1.415(2)	C(9)-C(9A)-C(4A)	120.18(13)
C(10)-C(10A)	1.402(2)	C(1)-C(9A)-C(4A)	117.62(13)
C(10)-C(23)	1.520(2)	C(10A)-C(10)-C(4A)	119.49(13)
C(11)-C(16)	1.386(2)	C(10A)-C(10)-C(23)	120.12(14)
C(11)-C(12)	1.397(2)	C(4A)-C(10)-C(23)	120.38(14)
C(12)-C(13)	1.377(3)	C(10)-C(10A)-C(5)	121.75(13)
C(13)-C(14)	1.370(3)	C(10)-C(10A)-C(8A)	120.64(13)
C(14)-C(15)	1.379(3)	C(5)-C(10A)-C(8A)	117.61(13)
C(15)-C(16)	1.391(2)	C(16)-C(11)-C(12)	118.11(15)
C(17)-C(18)	1.393(2)	C(16)-C(11)-P(1)	127.43(11)
C(17)-C(22)	1.396(2)	C(12)-C(11)-P(1)	114.40(13)
C(18)-C(19)	1.384(2)	C(13)-C(12)-C(11)	120.93(18)
C(19)-C(20)	1.379(3)	C(14)-C(13)-C(12)	120.39(17)
C(20)-C(21)	1.376(3)	C(13)-C(14)-C(15)	119.80(17)
C(21)-C(22)	1.385(2)	C(14)-C(15)-C(16)	120.14(19)
C(23)-C(24)	1.524(2)	C(11)-C(16)-C(15)	120.58(16)
		C(18)-C(17)-C(22)	118.05(14)
C(17)-P(1)-C(11)	106.62(7)	C(18)-C(17)-P(1)	122.73(11)
C(17)-P(1)-C(9)	106.50(7)	C(22)-C(17)-P(1)	118.36(11)
C(11)-P(1)-C(9)	101.29(7)	C(19)-C(18)-C(17)	120.68(15)
C(2)-C(1)-C(9A)	121.76(15)	C(20)-C(19)-C(18)	120.48(16)
C(21)-C(20)-C(19)	119.69(15)	C(21)-C(22)-C(17)	120.88(15)
C(20)-C(21)-C(22)	120.22(16)	C(10)-C(23)-C(24)	112.97(14)

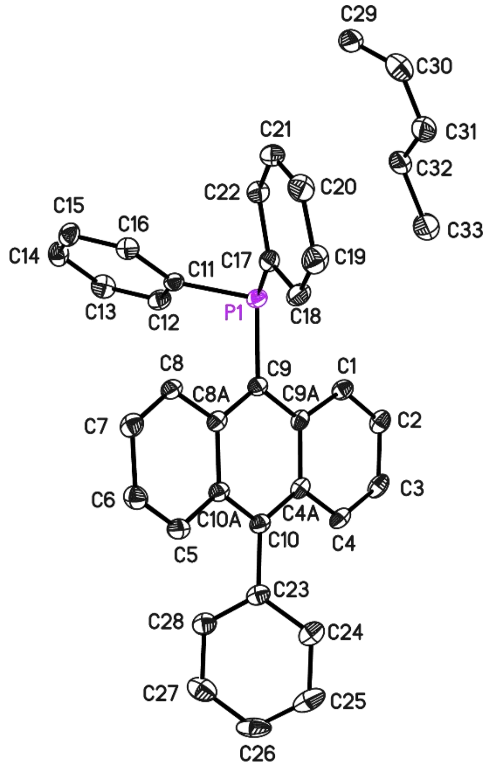

Fig. S33. Asymmetric unit of **4** with anisotropic displacement parameters at 50 % probability level. Hydrogen atoms are omitted for clarity. The toluene molecule is situated on a two-fold axis leading to disorder of the methyl hydrogen atoms.

Table S8. Bond lengths [Å] and angles [°] for **4**.			
P(1)-C(17)	1.825(2)	C(29)-C(30)#1	1.381(3)
P(1)-C(11)	1.8425(19)	C(29)-C(30)	1.382(3)
P(1)-C(9)	1.8568(19)	C(30)-C(31)	1.382(3)
C(1)-C(2)	1.359(3)	C(31)-C(32)	1.389(3)
C(1)-C(9A)	1.436(3)	C(32)-C(33)	1.512(5)
C(2)-C(3)	1.415(3)		
C(3)-C(4)	1.355(3)	C(17)-P(1)-C(11)	105.77(9)
C(4)-C(4A)	1.431(3)	C(17)-P(1)-C(9)	105.54(9)
C(4A)-C(10)	1.404(3)	C(11)-P(1)-C(9)	103.46(8)
C(4A)-C(9A)	1.446(3)	C(2)-C(1)-C(9A)	122.13(18)
C(5)-C(6)	1.356(3)	C(1)-C(2)-C(3)	120.58(18)
C(5)-C(10A)	1.432(3)	C(4)-C(3)-C(2)	119.92(18)
C(6)-C(7)	1.416(3)	C(3)-C(4)-C(4A)	121.68(18)
C(7)-C(8)	1.361(3)	C(10)-C(4A)-C(4)	121.08(17)
C(8)-C(8A)	1.432(3)	C(10)-C(4A)-C(9A)	120.08(16)
C(8A)-C(9)	1.415(3)	C(4)-C(4A)-C(9A)	118.84(17)

C(8A)-C(10A)	1.444(3)	C(6)-C(5)-C(10A)	121.28(18)
C(9)-C(9A)	1.418(3)	C(5)-C(6)-C(7)	119.97(18)
C(10)-C(10A)	1.410(3)	C(8)-C(7)-C(6)	120.89(18)
C(10)-C(23)	1.490(3)	C(7)-C(8)-C(8A)	121.63(18)
C(11)-C(16)	1.396(3)	C(9)-C(8A)-C(8)	122.71(17)
C(11)-C(12)	1.401(3)	C(9)-C(8A)-C(10A)	120.16(17)
C(12)-C(13)	1.382(3)	C(8)-C(8A)-C(10A)	117.12(16)
C(13)-C(14)	1.385(3)	C(8A)-C(9)-C(9A)	119.31(17)
C(14)-C(15)	1.384(3)	C(8A)-C(9)-P(1)	124.43(14)
C(15)-C(16)	1.389(3)	C(9A)-C(9)-P(1)	116.24(14)
C(17)-C(18)	1.397(3)	C(9)-C(9A)-C(1)	123.10(17)
C(17)-C(22)	1.400(3)	C(9)-C(9A)-C(4A)	120.16(17)
C(18)-C(19)	1.388(3)	C(1)-C(9A)-C(4A)	116.74(16)
C(19)-C(20)	1.387(3)	C(4A)-C(10)-C(10A)	120.01(17)
C(20)-C(21)	1.383(3)	C(4A)-C(10)-C(23)	120.01(17)
C(21)-C(22)	1.385(3)	C(10A)-C(10)-C(23)	119.98(17)
C(23)-C(28)	1.388(3)	C(10)-C(10A)-C(5)	120.81(17)
C(23)-C(24)	1.396(3)	C(10)-C(10A)-C(8A)	120.11(17)
C(24)-C(25)	1.387(3)	C(5)-C(10A)-C(8A)	119.06(17)
C(25)-C(26)	1.381(3)	C(16)-C(11)-C(12)	118.18(17)
C(26)-C(27)	1.380(3)	C(16)-C(11)-P(1)	126.49(15)
C(27)-C(28)	1.386(3)	C(12)-C(11)-P(1)	115.09(14)
C(13)-C(12)-C(11)	120.94(18)	C(28)-C(23)-C(10)	119.97(17)
C(12)-C(13)-C(14)	120.28(19)	C(24)-C(23)-C(10)	121.11(18)
C(15)-C(14)-C(13)	119.56(19)	C(25)-C(24)-C(23)	120.0(2)
C(14)-C(15)-C(16)	120.43(19)	C(26)-C(25)-C(24)	120.4(2)
C(15)-C(16)-C(11)	120.60(18)	C(27)-C(26)-C(25)	120.0(2)
C(18)-C(17)-C(22)	118.28(18)	C(26)-C(27)-C(28)	120.0(2)
C(18)-C(17)-P(1)	123.25(15)	C(27)-C(28)-C(23)	120.7(2)
C(22)-C(17)-P(1)	117.91(15)	C(30)#1-C(29)-C(30)	119.9(3)
C(19)-C(18)-C(17)	120.49(19)	C(29)-C(30)-C(31)	119.9(2)
C(20)-C(19)-C(18)	120.5(2)	C(30)-C(31)-C(32)	121.2(2)
C(21)-C(20)-C(19)	119.51(19)	C(31)-C(32)-C(31)#1	118.0(3)
C(20)-C(21)-C(22)	120.32(19)	C(31)-C(32)-C(33)	121.00(15)
C(21)-C(22)-C(17)	120.82(19)	C(31)#1-C(32)-C(33)	121.00(15)

C(28)-C(23)-C(24)	118.91(18)		

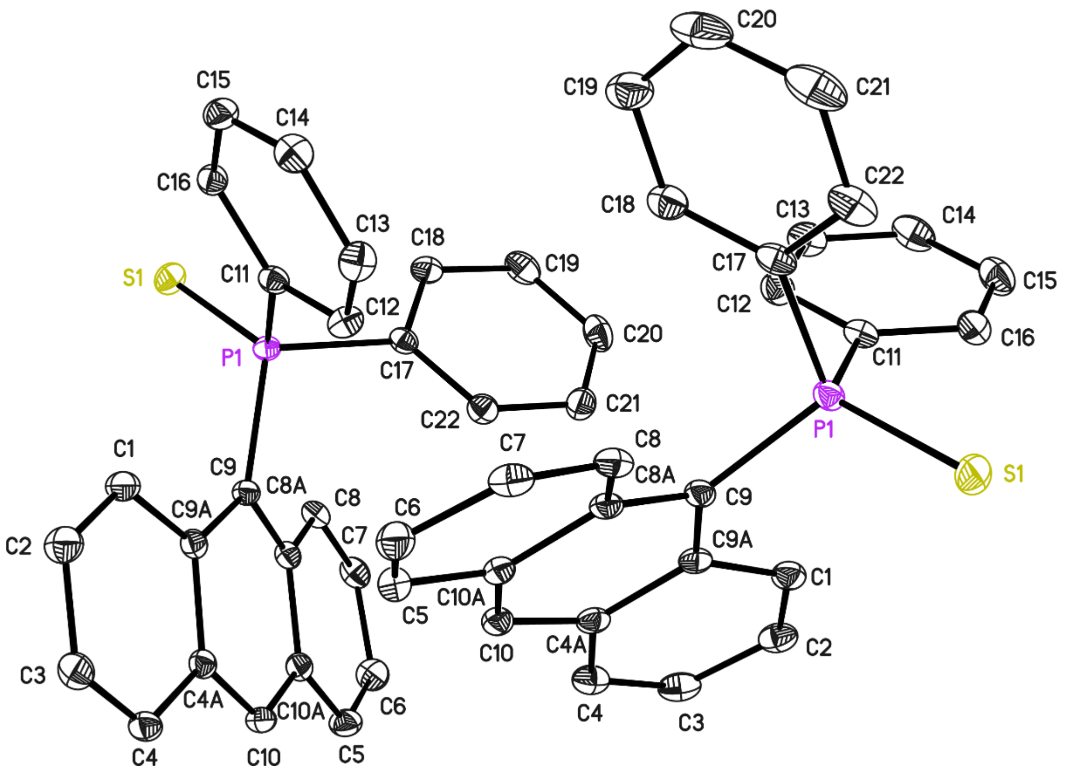

Fig. S34. Asymmetric unit of **5** with anisotropic displacement parameters at 50 % probability level. Hydrogen atoms are omitted for clarity.

Table S9. Bond lengths [Å] and angles [°] for **5**.			
C11-C21	1.3679(18)	C22-C32	1.423(2)
C11-C9A1	1.4301(18)	C32-C42	1.360(2)
S11-P11	1.9581(7)	C42-C4A2	1.4330(18)
P11-C111	1.8271(13)	C52-C62	1.3615(19)
P11-C171	1.8284(14)	C52-C10A2	1.4341(18)
P11-C91	1.8326(13)	C62-C72	1.4225(19)
C21-C31	1.4202(18)	C72-C82	1.3686(18)
C31-C41	1.3569(19)	C82-C8A2	1.4361(18)
C41-C4A1	1.4315(17)	C92-C9A2	1.4239(18)
C51-C61	1.3611(19)	C92-C8A2	1.4261(17)
C51-C10A1	1.4324(18)	C102-C4A2	1.3948(18)
C61-C71	1.4209(19)	C102-C10A2	1.3949(18)
C71-C81	1.3628(18)	C112-C122	1.3957(18)
C81-C8A1	1.4348(17)	C112-C162	1.3983(19)

C91-C8A1	1.4201(17)	C122-C132	1.3947(18)
C91-C9A1	1.4235(17)	C132-C142	1.389(2)
C101-C10A1	1.3932(17)	C142-C152	1.386(2)
C101-C4A1	1.3934(18)	C152-C162	1.3931(19)
C111-C161	1.3949(18)	C172-C222	1.3953(19)
C111-C121	1.4019(18)	C172-C182	1.399(2)
C121-C131	1.3901(19)	C182-C192	1.3919(19)
C131-C141	1.3928(19)	C192-C202	1.394(2)
C141-C151	1.3887(19)	C202-C212	1.381(3)
C151-C161	1.3973(18)	C212-C222	1.394(2)
C171-C181	1.3943(17)	C9A2-C4A2	1.4409(17)
C171-C221	1.3987(18)	C10A2-C8A2	1.4433(17)
C181-C191	1.3935(19)		
C191-C201	1.388(2)	C21-C11-C9A1	121.56(12)
C201-C211	1.3929(19)	C111-P11-C171	101.65(6)
C211-C221	1.3887(19)	C111-P11-C91	109.03(6)
C9A1-C4A1	1.4421(17)	C171-P11-C91	103.07(6)
C10A1-C8A1	1.4413(17)	C111-P11-S11	112.47(5)
C12-C22	1.3668(19)	C171-P11-S11	113.08(5)
C12-C9A2	1.4354(18)	C91-P11-S11	116.20(5)
P12-C172	1.8290(14)	C11-C21-C31	121.33(12)
P12-C112	1.8311(13)	C41-C31-C21	119.20(12)
P12-C92	1.8327(13)	C31-C41-C4A1	121.48(12)
P12-S12	1.9525(8)	C61-C51-C10A1	120.78(12)
C51-C61-C71	119.87(12)	C112-P12-C92	108.15(6)
C81-C71-C61	121.03(12)	C172-P12-S12	113.05(5)
C71-C81-C8A1	121.49(12)	C112-P12-S12	112.79(5)
C8A1-C91-C9A1	119.94(11)	C92-P12-S12	113.97(5)
C8A1-C91-P11	116.66(9)	C12-C22-C32	121.15(12)
C9A1-C91-P11	123.30(9)	C42-C32-C22	119.71(13)
C10A1-C101-C4A1	121.57(11)	C32-C42-C4A2	120.94(12)
C161-C111-C121	119.68(12)	C62-C52-C10A2	120.80(12)
C161-C111-P11	119.47(10)	C52-C62-C72	119.80(12)
C121-C111-P11	120.79(10)	C82-C72-C62	121.14(12)
C131-C121-C111	120.15(12)	C72-C82-C8A2	121.35(12)

C121-C131-C141	119.93(12)	C9A2-C92-C8A2	119.84(11)
C151-C141-C131	120.21(12)	C9A2-C92-P12	119.46(9)
C141-C151-C161	120.13(12)	C8A2-C92-P12	120.59(10)
C111-C161-C151	119.88(12)	C4A2-C102-C10A2	121.51(11)
C181-C171-C221	119.51(12)	C122-C112-C162	119.27(12)
C181-C171-P11	119.16(10)	C122-C112-P12	121.94(10)
C221-C171-P11	121.31(9)	C162-C112-P12	118.61(10)
C191-C181-C171	119.99(12)	C132-C122-C112	120.08(12)
C201-C191-C181	120.18(12)	C142-C132-C122	120.23(13)
C191-C201-C211	120.11(12)	C152-C142-C132	120.01(12)
C221-C211-C201	119.82(12)	C142-C152-C162	120.04(13)
C211-C221-C171	120.36(12)	C152-C162-C112	120.35(13)
C91-C9A1-C11	124.55(11)	C222-C172-C182	119.63(13)
C91-C9A1-C4A1	118.62(11)	C222-C172-P12	118.34(11)
C11-C9A1-C4A1	116.73(11)	C182-C172-P12	122.00(10)
C101-C4A1-C41	120.31(11)	C192-C182-C172	119.90(13)
C101-C4A1-C9A1	120.04(11)	C182-C192-C202	120.15(15)
C41-C4A1-C9A1	119.63(11)	C212-C202-C192	120.00(14)
C101-C10A1-C51	120.96(11)	C202-C212-C222	120.36(14)
C101-C10A1-C8A1	119.34(11)	C212-C222-C172	119.97(14)
C51-C10A1-C8A1	119.70(11)	C92-C9A2-C12	123.95(11)
C91-C8A1-C81	123.81(11)	C92-C9A2-C4A2	118.95(11)
C91-C8A1-C10A1	119.32(11)	C12-C9A2-C4A2	116.96(12)
C81-C8A1-C10A1	116.86(11)	C102-C4A2-C42	120.46(12)
C22-C12-C9A2	121.31(12)	C102-C4A2-C9A2	119.79(12)
C172-P12-C112	100.95(6)	C42-C4A2-C9A2	119.72(12)
C172-P12-C92	106.98(6)	C102-C10A2-C52	120.36(11)
C102-C10A2-C8A2	119.77(11)	C92-C8A2-C10A2	118.78(11)
C52-C10A2-C8A2	119.83(12)	C82-C8A2-C10A2	116.82(11)
C92-C8A2-C82	124.26(11)		

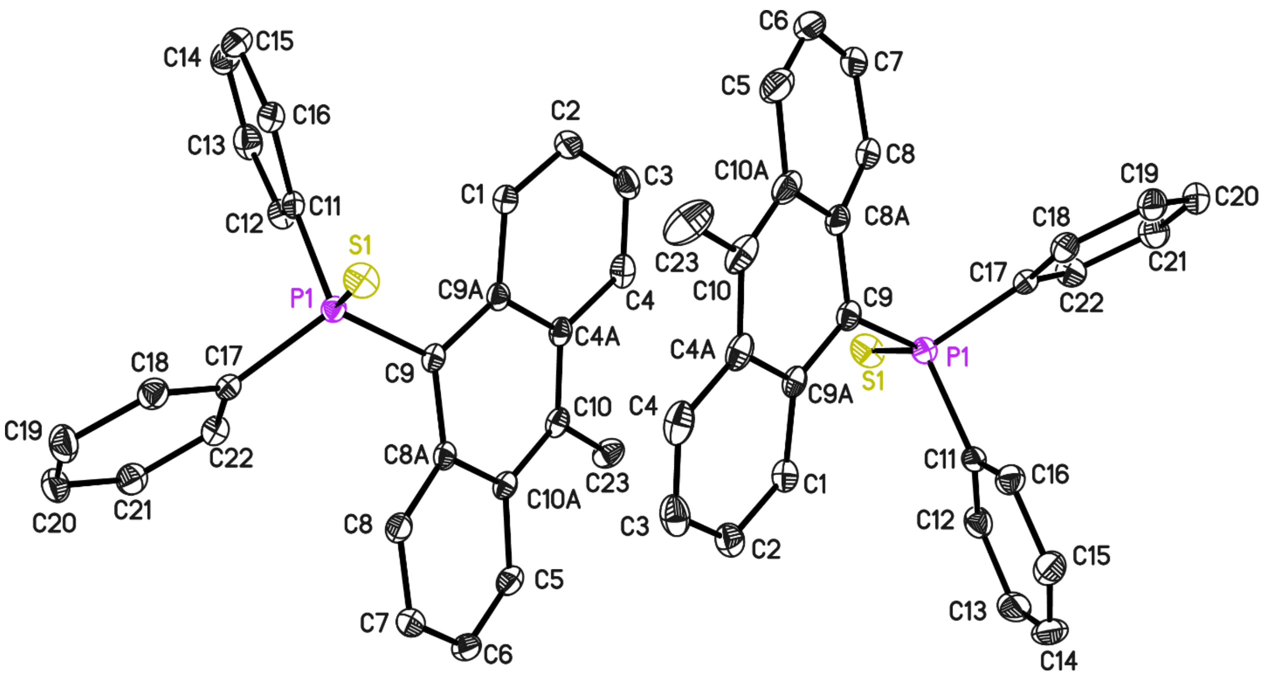

Fig. S35. Asymmetric unit of **6** with anisotropic displacement parameters at 50 % probability level. Hydrogen atoms are omitted for clarity. Hydrogen atoms attached to one C23 are disordered about two positions. Occupancy of the major component refines to 0.76(2).

Table S10. Bond lengths [Å] and angles [°] for **6**.			
S11-P11	1.9602(6)	C12-C9A2	1.432(2)
P11-C91	1.8248(14)	C22-C32	1.420(2)
P11-C171	1.8268(14)	C32-C42	1.354(2)
P11-C111	1.8336(14)	C42-C4A2	1.435(2)
C11-C21	1.362(2)	C52-C62	1.355(3)
C11-C9A1	1.4308(19)	C52-C10A2	1.434(2)
C21-C31	1.416(2)	C62-C72	1.416(3)
C31-C41	1.356(2)	C72-C82	1.368(2)
C41-C4A1	1.4348(19)	C82-C8A2	1.431(2)
C51-C61	1.359(2)	C92-C8A2	1.4164(19)
C51-C10A1	1.4313(19)	C92-C9A2	1.420(2)
C61-C71	1.419(2)	C102-C10A2	1.404(2)
C71-C81	1.366(2)	C102-C4A2	1.407(2)
C81-C8A1	1.4324(19)	C102-C232	1.510(2)
C91-C8A1	1.4144(18)	C112-C122	1.389(2)
C91-C9A1	1.4164(19)	C112-C162	1.399(2)
C101-C4A1	1.4077(19)	C122-C132	1.393(2)
C101-C10A1	1.4114(19)	C132-C142	1.380(3)

C101-C231	1.5098(19)	C142-C152	1.387(2)
C111-C161	1.3923(19)	C152-C162	1.387(2)
C111-C121	1.398(2)	C172-C222	1.391(2)
C121-C131	1.388(2)	C172-C182	1.399(2)
C131-C141	1.391(2)	C182-C192	1.388(2)
C141-C151	1.383(2)	C192-C202	1.386(2)
C151-C161	1.396(2)	C202-C212	1.383(2)
C171-C181	1.3911(19)	C212-C222	1.390(2)
C171-C221	1.399(2)	C4A2-C9A2	1.4441(19)
C181-C191	1.396(2)	C8A2-C10A2	1.443(2)
C191-C201	1.380(2)		
C201-C211	1.388(2)	C91-P11-C171	108.27(6)
C211-C221	1.388(2)	C91-P11-C111	106.26(6)
C4A1-C9A1	1.4419(19)	C171-P11-C111	98.91(6)
C8A1-C10A1	1.4379(19)	C91-P11-S11	114.98(5)
S12-P12	1.9606(6)	C171-P11-S11	113.58(5)
P12-C92	1.8201(14)	C111-P11-S11	113.42(5)
P12-C172	1.8252(14)	C21-C11-C9A1	121.92(13)
P12-C112	1.8253(14)	C11-C21-C31	120.30(14)
C12-C22	1.362(2)	C41-C31-C21	120.09(13)
C31-C41-C4A1	121.87(14)	C51-C10A1-C8A1	117.99(12)
C61-C51-C10A1	121.98(13)	C92-P12-C172	108.08(6)
C51-C61-C71	119.90(13)	C92-P12-C112	107.77(6)
C81-C71-C61	120.26(13)	C172-P12-C112	98.92(6)
C71-C81-C8A1	121.54(13)	C92-P12-S12	113.44(5)
C8A1-C91-C9A1	119.28(12)	C172-P12-S12	114.25(5)
C8A1-C91-P11	121.23(10)	C112-P12-S12	113.25(5)
C9A1-C91-P11	119.48(10)	C22-C12-C9A2	121.99(13)
C4A1-C101-C10A1	119.18(12)	C12-C22-C32	120.51(14)
C4A1-C101-C231	120.93(13)	C42-C32-C22	119.74(14)
C10A1-C101-C231	119.87(13)	C32-C42-C4A2	122.07(14)
C161-C111-C121	119.92(13)	C62-C52-C10A2	121.84(16)
C161-C111-P11	119.90(11)	C52-C62-C72	120.19(15)
C121-C111-P11	120.12(10)	C82-C72-C62	120.41(16)
C131-C121-C111	119.80(13)	C72-C82-C8A2	121.31(15)

C121-C131-C141	120.31(14)	C8A2-C92-C9A2	119.46(13)
C151-C141-C131	119.93(14)	C8A2-C92-P12	119.19(11)
C141-C151-C161	120.31(14)	C9A2-C92-P12	121.26(10)
C111-C161-C151	119.72(14)	C10A2-C102-C4A2	119.63(14)
C181-C171-C221	119.63(13)	C10A2-C102-C232	121.41(14)
C181-C171-P11	117.74(11)	C4A2-C102-C232	118.91(15)
C221-C171-P11	122.32(10)	C122-C112-C162	119.97(13)
C171-C181-C191	119.97(13)	C122-C112-P12	119.10(11)
C201-C191-C181	120.24(14)	C162-C112-P12	120.92(11)
C191-C201-C211	119.90(14)	C112-C122-C132	119.71(15)
C201-C211-C221	120.48(14)	C142-C132-C122	120.22(15)
C211-C221-C171	119.74(13)	C132-C142-C152	120.25(15)
C101-C4A1-C41	121.91(13)	C162-C152-C142	120.12(16)
C101-C4A1-C9A1	119.99(12)	C152-C162-C112	119.69(14)
C41-C4A1-C9A1	118.09(12)	C222-C172-C182	119.71(13)
C91-C8A1-C81	122.89(12)	C222-C172-P12	118.15(11)
C91-C8A1-C10A1	119.09(12)	C182-C172-P12	121.66(11)
C81-C8A1-C10A1	117.98(12)	C192-C182-C172	119.88(14)
C91-C9A1-C11	122.51(12)	C202-C192-C182	120.21(14)
C91-C9A1-C4A1	119.72(12)	C212-C202-C192	119.95(14)
C11-C9A1-C4A1	117.71(12)	C202-C212-C222	120.44(14)
C101-C10A1-C51	121.34(12)	C212-C222-C172	119.77(14)
C101-C10A1-C8A1	120.63(12)	C102-C4A2-C42	121.27(14)
C102-C4A2-C9A2	120.44(14)	C92-C9A2-C4A2	119.01(13)
C42-C4A2-C9A2	118.27(13)	C12-C9A2-C4A2	117.38(13)
C92-C8A2-C82	122.28(14)	C102-C10A2-C52	121.86(14)
C92-C8A2-C10A2	119.46(13)	C102-C10A2-C8A2	120.12(13)
C82-C8A2-C10A2	118.15(13)	C52-C10A2-C8A2	117.92(15)
C92-C9A2-C12	123.55(13)		

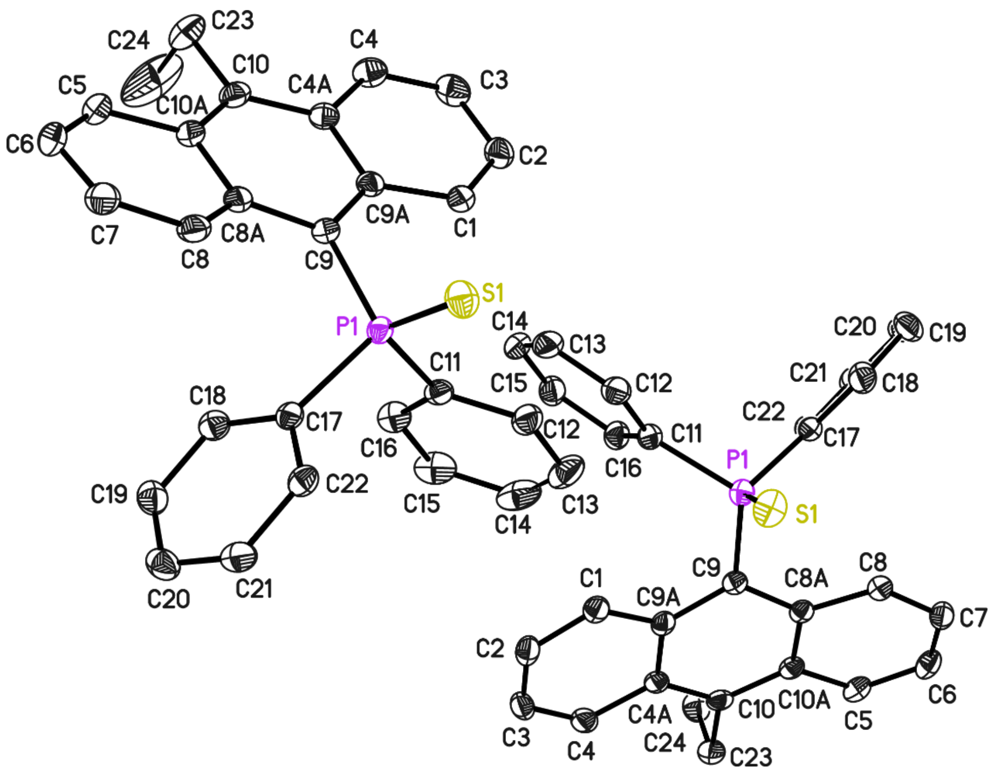

Fig. S36. Asymmetric unit of **7** with anisotropic displacement parameters at 50 % probability level. Hydrogen atoms are omitted for clarity.

Table S11. Bond lengths [Å] and angles [°] for **7**.

S11-P11	1.9530(8)	C12-C22	1.359(2)
P11-C91	1.8243(16)	C12-C9A2	1.434(2)
P11-C171	1.8282(16)	C22-C32	1.410(3)
P11-C111	1.8295(15)	C32-C42	1.358(2)
C11-C21	1.361(2)	C42-C4A2	1.436(2)
C11-C9A1	1.435(2)	C52-C62	1.359(2)
C21-C31	1.410(2)	C52-C10A2	1.438(2)
C31-C41	1.358(2)	C62-C72	1.413(2)
C41-C4A1	1.433(2)	C72-C82	1.365(2)
C51-C61	1.358(2)	C82-C8A2	1.430(2)
C51-C10A1	1.439(2)	C92-C9A2	1.413(2)
C61-C71	1.414(3)	C92-C8A2	1.418(2)
C71-C81	1.364(2)	C102-C10A2	1.410(2)
C81-C8A1	1.432(2)	C102-C4A2	1.412(2)
C91-C9A1	1.414(2)	C102-C232	1.510(2)
C91-C8A1	1.418(2)	C112-C122	1.391(2)

C111-C121	1.392(2)	C112-C162	1.392(2)
C111-C161	1.393(2)	C122-C132	1.394(2)
C121-C131	1.396(2)	C132-C142	1.377(3)
C131-C141	1.376(3)	C142-C152	1.390(3)
C141-C151	1.386(3)	C152-C162	1.386(2)
C151-C161	1.387(2)	C172-C182	1.387(2)
C171-C221	1.390(2)	C172-C222	1.398(2)
C171-C181	1.398(2)	C182-C192	1.393(2)
C181-C191	1.389(2)	C192-C202	1.377(3)
C191-C201	1.389(2)	C202-C212	1.384(3)
C201-C211	1.385(2)	C212-C222	1.382(2)
C211-C221	1.395(2)	C232-C242	1.529(2)
C101-C10A1	1.406(2)	C4A2-C9A2	1.439(2)
C101-C4A1	1.410(2)	C8A2-C10A2	1.435(2)
C101-C231	1.512(2)		
C231-C241	1.513(3)	C91-P11-C171	108.33(7)
C4A1-C9A1	1.442(2)	C91-P11-C111	108.95(7)
C8A1-C10A1	1.441(2)	C171-P11-C111	98.50(7)
S12-P12	1.9554(7)	C91-P11-S11	113.43(6)
P12-C92	1.8195(16)	C171-P11-S11	113.58(6)
P12-C112	1.8267(17)	C111-P11-S11	112.97(6)
P12-C172	1.8270(16)	C21-C11-C9A1	121.74(15)
C11-C21-C31	120.41(15)	C11-C9A1-C4A1	117.61(13)
C41-C31-C21	120.29(15)	C101-C10A1-C51	122.06(15)
C31-C41-C4A1	121.74(15)	C101-C10A1-C8A1	120.52(14)
C61-C51-C10A1	122.28(16)	C51-C10A1-C8A1	117.39(14)
C51-C61-C71	119.69(15)	C92-P12-C112	107.67(7)
C81-C71-C61	120.49(16)	C92-P12-C172	106.09(7)
C71-C81-C8A1	121.62(16)	C112-P12-C172	99.29(7)
C9A1-C91-C8A1	119.50(14)	C92-P12-S12	114.89(5)
C9A1-C91-P11	120.74(11)	C112-P12-S12	113.33(6)
C8A1-C91-P11	119.69(11)	C172-P12-S12	114.18(6)
C121-C111-C161	119.93(14)	C22-C12-C9A2	121.50(15)
C121-C111-P11	118.84(12)	C12-C22-C32	120.43(15)
C161-C111-P11	120.96(12)	C42-C32-C22	120.45(15)

C111-C121-C131	119.82(17)	C32-C42-C4A2	121.53(16)
C141-C131-C121	119.88(17)	C62-C52-C10A2	121.63(15)
C131-C141-C151	120.47(16)	C52-C62-C72	120.15(14)
C141-C151-C161	120.20(17)	C82-C72-C62	120.20(15)
C151-C161-C111	119.68(16)	C72-C82-C8A2	121.61(15)
C221-C171-C181	119.72(14)	C9A2-C92-C8A2	119.19(14)
C221-C171-P11	118.17(12)	C9A2-C92-P12	120.84(11)
C181-C171-P11	121.77(12)	C8A2-C92-P12	119.97(11)
C191-C181-C171	119.80(15)	C10A2-C102-C4A2	119.48(14)
C201-C191-C181	120.33(15)	C10A2-C102-C232	119.47(14)
C211-C201-C191	120.00(15)	C4A2-C102-C232	121.05(14)
C201-C211-C221	120.06(15)	C122-C112-C162	119.89(15)
C171-C221-C211	120.08(15)	C122-C112-P12	118.93(13)
C10A1-C101-C4A1	119.36(14)	C162-C112-P12	121.09(12)
C10A1-C101-C231	120.91(14)	C112-C122-C132	119.70(16)
C4A1-C101-C231	119.73(15)	C142-C132-C122	120.15(16)
C101-C231-C241	111.14(16)	C132-C142-C152	120.33(16)
C101-C4A1-C41	121.36(14)	C162-C152-C142	119.86(17)
C101-C4A1-C9A1	120.46(14)	C152-C162-C112	120.06(16)
C41-C4A1-C9A1	118.17(14)	C182-C172-C222	119.46(15)
C91-C8A1-C81	122.70(14)	C182-C172-P12	120.12(13)
C91-C8A1-C10A1	119.29(14)	C222-C172-P12	120.28(12)
C81-C8A1-C10A1	117.94(14)	C172-C182-C192	119.83(17)
C91-C9A1-C11	122.78(14)	C202-C192-C182	120.37(16)
C91-C9A1-C4A1	119.49(13)	C192-C202-C212	120.08(16)
C222-C212-C202	120.07(17)	C82-C8A2-C10A2	118.04(13)
C212-C222-C172	120.18(15)	C92-C9A2-C12	121.95(14)
C102-C232-C242	110.59(13)	C92-C9A2-C4A2	119.89(13)
C102-C4A2-C42	122.06(14)	C12-C9A2-C4A2	118.08(14)
C102-C4A2-C9A2	120.02(13)	C102-C10A2-C8A2	120.43(13)
C42-C4A2-C9A2	117.91(14)	C102-C10A2-C52	121.76(14)
C92-C8A2-C82	122.39(14)	C8A2-C10A2-C52	117.80(14)
C92-C8A2-C10A2	119.47(13)		

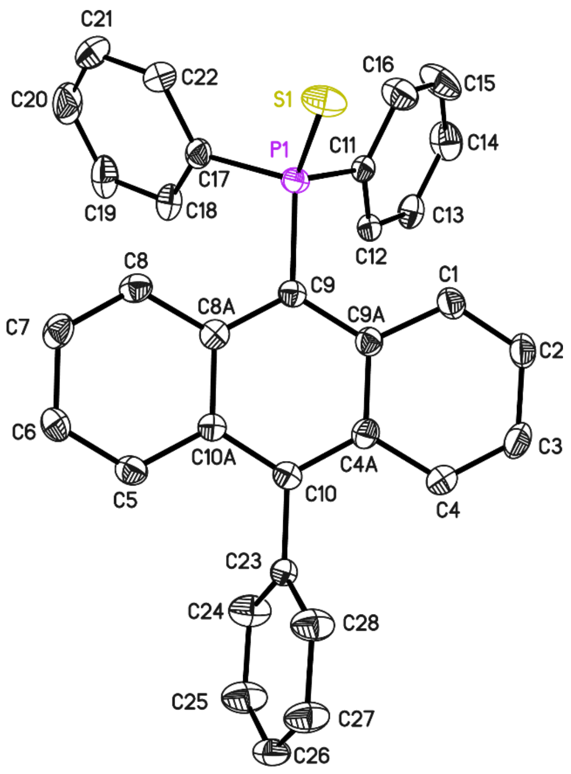

Fig. S37. Asymmetric unit of **8** with anisotropic displacement parameters at 50 % probability level. Hydrogen atoms are omitted for clarity.

Table S12. Bond lengths [Å] and angles [°] for **8**.

P(1)-C(11)	1.823(3)	C(27)-C(28)	1.386(4)
P(1)-C(17)	1.823(3)		
P(1)-C(9)	1.830(3)	C(11)-P(1)-C(17)	98.24(13)
P(1)-S(1)	1.9550(12)	C(11)-P(1)-C(9)	108.53(15)
C(1)-C(2)	1.364(5)	C(17)-P(1)-C(9)	108.98(15)
C(1)-C(9A)	1.427(4)	C(11)-P(1)-S(1)	113.65(12)
C(2)-C(3)	1.420(5)	C(17)-P(1)-S(1)	113.32(13)
C(3)-C(4)	1.359(5)	C(9)-P(1)-S(1)	113.07(9)
C(4)-C(4A)	1.426(4)	C(2)-C(1)-C(9A)	121.7(3)
C(4A)-C(10)	1.402(4)	C(1)-C(2)-C(3)	120.6(3)
C(4A)-C(9A)	1.442(5)	C(4)-C(3)-C(2)	119.5(3)
C(5)-C(6)	1.362(4)	C(3)-C(4)-C(4A)	121.9(4)
C(5)-C(10A)	1.429(4)	C(10)-C(4A)-C(4)	121.5(3)
C(10)-C(10A)	1.409(4)	C(10)-C(4A)-C(9A)	120.0(3)
C(10)-C(23)	1.504(3)	C(4)-C(4A)-C(9A)	118.5(3)
C(6)-C(7)	1.415(5)	C(6)-C(5)-C(10A)	121.6(3)

C(10A)-C(8A)	1.438(4)	C(4A)-C(10)-C(10A)	120.0(2)
C(7)-C(8)	1.371(5)	C(4A)-C(10)-C(23)	119.9(3)
C(8)-C(8A)	1.433(4)	C(10A)-C(10)-C(23)	120.0(3)
C(8A)-C(9)	1.414(4)	C(5)-C(6)-C(7)	119.5(3)
C(12)-C(13)	1.383(4)	C(10)-C(10A)-C(5)	120.3(3)
C(12)-C(11)	1.399(5)	C(10)-C(10A)-C(8A)	120.4(3)
C(9)-C(9A)	1.419(4)	C(5)-C(10A)-C(8A)	119.3(3)
C(14)-C(15)	1.375(6)	C(8)-C(7)-C(6)	120.8(3)
C(14)-C(13)	1.380(5)	C(7)-C(8)-C(8A)	121.7(3)
C(11)-C(16)	1.379(5)	C(9)-C(8A)-C(8)	123.5(3)
C(15)-C(16)	1.383(5)	C(9)-C(8A)-C(10A)	119.4(3)
C(17)-C(22)	1.385(5)	C(8)-C(8A)-C(10A)	117.0(3)
C(17)-C(18)	1.389(5)	C(13)-C(12)-C(11)	119.9(3)
C(18)-C(19)	1.394(5)	C(8A)-C(9)-C(9A)	119.5(2)
C(19)-C(20)	1.391(6)	C(8A)-C(9)-P(1)	121.1(2)
C(20)-C(21)	1.384(7)	C(9A)-C(9)-P(1)	119.3(2)
C(21)-C(22)	1.391(5)	C(9)-C(9A)-C(1)	123.0(3)
C(23)-C(28)	1.381(4)	C(9)-C(9A)-C(4A)	119.5(3)
C(23)-C(24)	1.387(4)	C(1)-C(9A)-C(4A)	117.4(3)
C(24)-C(25)	1.384(4)	C(15)-C(14)-C(13)	119.5(3)
C(25)-C(26)	1.373(4)	C(14)-C(13)-C(12)	120.4(4)
C(26)-C(27)	1.379(4)	C(16)-C(11)-C(12)	119.1(3)
C(16)-C(11)-P(1)	117.7(3)	C(20)-C(21)-C(22)	119.8(4)
C(12)-C(11)-P(1)	122.8(3)	C(17)-C(22)-C(21)	120.4(4)
C(14)-C(15)-C(16)	120.6(4)	C(28)-C(23)-C(24)	118.6(3)
C(11)-C(16)-C(15)	120.3(4)	C(28)-C(23)-C(10)	121.2(2)
C(22)-C(17)-C(18)	119.7(3)	C(24)-C(23)-C(10)	120.1(2)
C(22)-C(17)-P(1)	118.3(3)	C(25)-C(24)-C(23)	120.4(3)
C(18)-C(17)-P(1)	121.8(3)	C(26)-C(25)-C(24)	120.8(3)
C(17)-C(18)-C(19)	120.3(4)	C(25)-C(26)-C(27)	119.0(3)
C(20)-C(19)-C(18)	119.4(4)	C(26)-C(27)-C(28)	120.6(3)
C(21)-C(20)-C(19)	120.4(4)	C(23)-C(28)-C(27)	120.5(3)

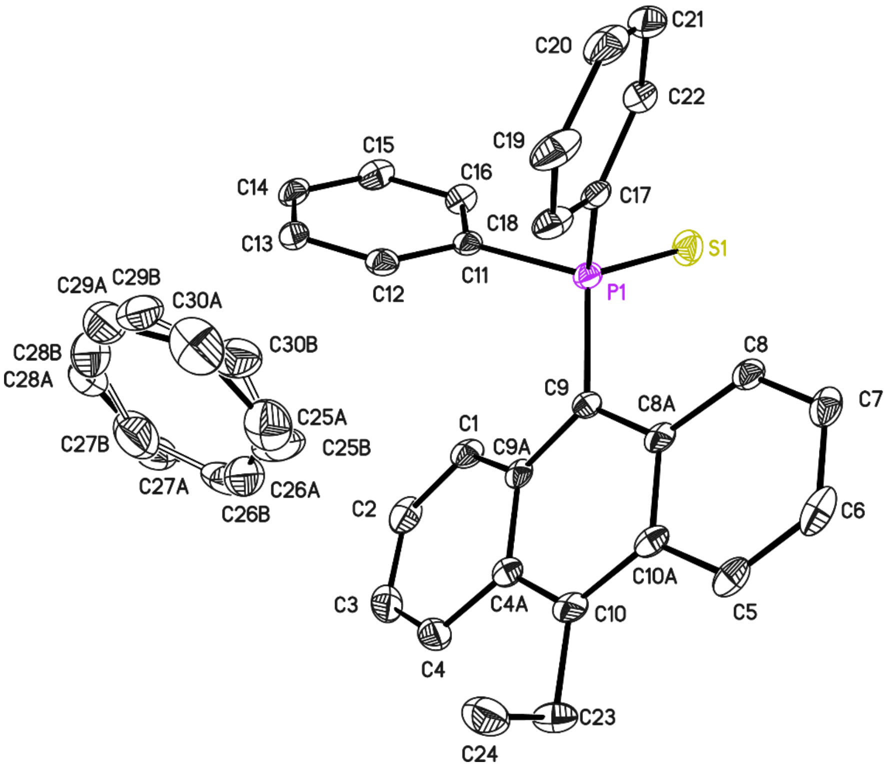

Fig. S38. Asymmetric unit of **7a** with anisotropic displacement parameters at 50 % probability level. Hydrogen atoms are omitted for clarity. The benzene molecule is disordered over two positions. It was refined with distance restraints and restraints for the anisotropic displacement parameters.[4] The occupancy of the major component refined to 0.61(2).

Table S13. Bond lengths [Å] and angles [°] for **7a.**

S(1)-P(1)	1.9628(8)	C(32A)-C(33A)	1.381(6)
C(1)-C(2)	1.360(3)	C(33A)-C(34A)	1.382(7)
C(1)-C(9A)	1.431(2)	C(29B)-C(30B)	1.372(9)
P(1)-C(11)	1.8229(18)	C(29B)-C(34B)	1.384(10)
P(1)-C(9)	1.8304(17)	C(30B)-C(31B)	1.375(10)
P(1)-C(17)	1.8336(18)	C(31B)-C(32B)	1.377(11)
C(2)-C(3)	1.408(3)	C(32B)-C(33B)	1.370(11)
C(3)-C(4)	1.356(3)	C(33B)-C(34B)	1.381(10)
C(4)-C(4A)	1.435(2)		
C(4A)-C(10)	1.407(2)	C(2)-C(1)-C(9A)	122.02(17)
C(4A)-C(9A)	1.444(2)	C(11)-P(1)-C(9)	109.52(8)
C(5)-C(6)	1.360(3)	C(11)-P(1)-C(17)	99.99(8)
C(5)-C(10A)	1.431(2)	C(9)-P(1)-C(17)	105.62(8)
C(10)-C(10A)	1.411(3)	C(11)-P(1)-S(1)	113.02(6)
C(10)-C(23)	1.514(2)	C(9)-P(1)-S(1)	114.23(6)
C(6)-C(7)	1.412(3)	C(17)-P(1)-S(1)	113.32(7)

C(10A)-C(8A)	1.439(2)	C(1)-C(2)-C(3)	120.28(18)
C(7)-C(8)	1.363(2)	C(4)-C(3)-C(2)	120.12(17)
C(8)-C(8A)	1.430(2)	C(3)-C(4)-C(4A)	122.07(17)
C(8A)-C(9)	1.419(2)	C(10)-C(4A)-C(4)	121.96(16)
C(12)-C(13)	1.385(2)	C(10)-C(4A)-C(9A)	120.43(16)
C(12)-C(11)	1.391(2)	C(4)-C(4A)-C(9A)	117.60(16)
C(9)-C(9A)	1.418(2)	C(6)-C(5)-C(10A)	121.98(17)
C(14)-C(13)	1.383(3)	C(4A)-C(10)-C(10A)	119.57(15)
C(14)-C(15)	1.384(3)	C(4A)-C(10)-C(23)	120.82(16)
C(23)-C(24)	1.527(3)	C(10A)-C(10)-C(23)	119.60(16)
C(15)-C(16)	1.391(3)	C(5)-C(6)-C(7)	119.95(17)
C(16)-C(11)	1.389(2)	C(10)-C(10A)-C(5)	121.60(16)
C(17)-C(18)	1.393(3)	C(10)-C(10A)-C(8A)	120.46(16)
C(17)-C(22)	1.393(3)	C(5)-C(10A)-C(8A)	117.94(16)
C(18)-C(19)	1.394(3)	C(8)-C(7)-C(6)	120.41(17)
C(19)-C(20)	1.384(3)	C(7)-C(8)-C(8A)	121.66(16)
C(20)-C(21)	1.380(3)	C(9)-C(8A)-C(8)	122.41(15)
C(21)-C(22)	1.391(3)	C(9)-C(8A)-C(10A)	119.64(15)
C(29A)-C(30A)	1.373(8)	C(8)-C(8A)-C(10A)	117.92(15)
C(29A)-C(34A)	1.380(8)	C(13)-C(12)-C(11)	120.05(16)
C(30A)-C(31A)	1.387(7)	C(9A)-C(9)-C(8A)	119.46(15)
C(31A)-C(32A)	1.381(7)	C(9A)-C(9)-P(1)	122.45(13)
C(8A)-C(9)-P(1)	118.08(12)	C(20)-C(19)-C(18)	119.9(2)
C(9)-C(9A)-C(1)	122.93(15)	C(21)-C(20)-C(19)	120.26(19)
C(9)-C(9A)-C(4A)	119.46(15)	C(20)-C(21)-C(22)	120.01(19)
C(1)-C(9A)-C(4A)	117.53(15)	C(21)-C(22)-C(17)	120.4(2)
C(13)-C(14)-C(15)	120.20(17)	C(30A)-C(29A)-C(34A)	119.9(7)
C(14)-C(13)-C(12)	120.10(17)	C(29A)-C(30A)-C(31A)	119.9(8)
C(10)-C(23)-C(24)	111.24(15)	C(32A)-C(31A)-C(30A)	120.2(8)
C(14)-C(15)-C(16)	119.97(17)	C(31A)-C(32A)-C(33A)	119.7(7)
C(11)-C(16)-C(15)	119.90(17)	C(32A)-C(33A)-C(34A)	119.8(6)
C(16)-C(11)-C(12)	119.77(16)	C(29A)-C(34A)-C(33A)	120.4(6)
C(16)-C(11)-P(1)	119.80(13)	C(30B)-C(29B)-C(34B)	119.9(10)
C(12)-C(11)-P(1)	120.40(13)	C(29B)-C(30B)-C(31B)	120.0(11)
C(18)-C(17)-C(22)	119.13(17)	C(30B)-C(31B)-C(32B)	120.1(12)

C(18)-C(17)-P(1)	122.32(13)	C(33B)-C(32B)-C(31B)	120.0(12)
C(22)-C(17)-P(1)	118.44(15)	C(32B)-C(33B)-C(34B)	120.1(12)
C(17)-C(18)-C(19)	120.24(18)	C(33B)-C(34B)-C(29B)	119.7(10)

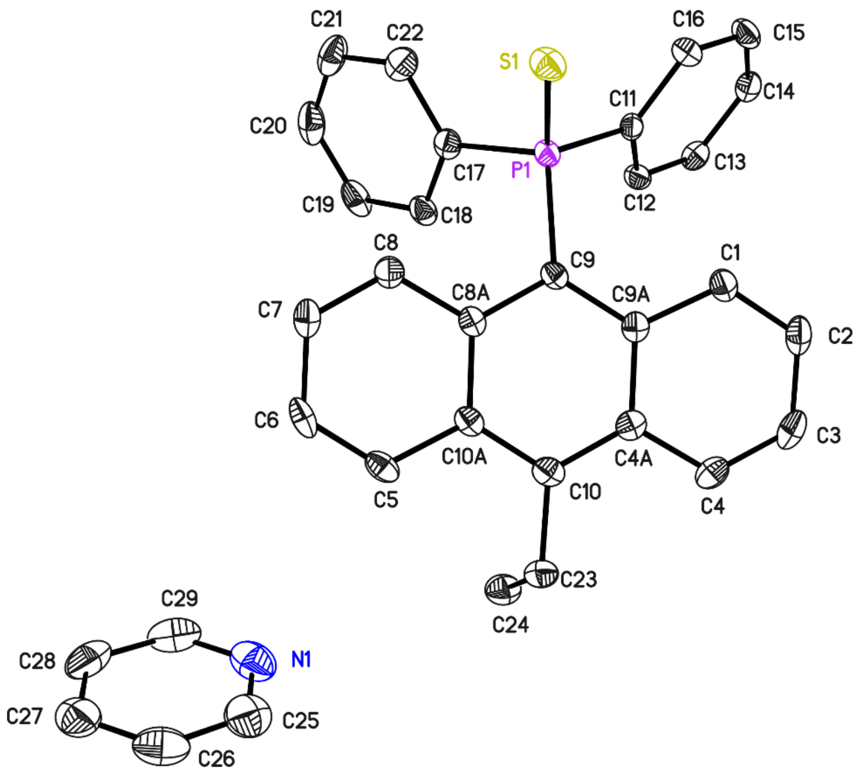

Fig. S39. Asymmetric unit of **7b** with anisotropic displacement parameters at 50 % probability level. Hydrogen atoms are omitted for clarity.

Table 14. Bond lengths [Å] and angles [°] for **7b**.			
P(1)-C(11)	1.821(2)	C(27)-C(28)	1.382(4)
P(1)-C(9)	1.826(2)	C(28)-C(29)	1.367(4)
P(1)-C(17)	1.836(2)		
P(1)-S(1)	1.9617(11)	C(11)-P(1)-C(9)	109.52(9)
C(1)-C(2)	1.363(3)	C(11)-P(1)-C(17)	100.39(9)
C(1)-C(9A)	1.436(3)	C(9)-P(1)-C(17)	105.20(9)
N(1)-C(25)	1.331(3)	C(11)-P(1)-S(1)	112.61(7)
N(1)-C(29)	1.334(3)	C(9)-P(1)-S(1)	114.95(7)
C(2)-C(3)	1.408(3)	C(17)-P(1)-S(1)	112.97(8)
C(3)-C(4)	1.354(3)	C(2)-C(1)-C(9A)	121.9(2)
C(4)-C(4A)	1.437(3)	C(25)-N(1)-C(29)	116.1(2)

C(4A)-C(10)	1.409(3)	C(1)-C(2)-C(3)	120.37(19)
C(4A)-C(9A)	1.441(3)	C(4)-C(3)-C(2)	120.07(19)
C(5)-C(6)	1.357(3)	C(3)-C(4)-C(4A)	122.1(2)
C(5)-C(10A)	1.432(3)	C(10)-C(4A)-C(4)	121.81(18)
C(10)-C(10A)	1.410(3)	C(10)-C(4A)-C(9A)	120.44(18)
C(10)-C(23)	1.513(3)	C(4)-C(4A)-C(9A)	117.75(18)
C(6)-C(7)	1.409(3)	C(6)-C(5)-C(10A)	121.72(19)
C(10A)-C(8A)	1.440(3)	C(4A)-C(10)-C(10A)	119.48(18)
C(7)-C(8)	1.359(3)	C(4A)-C(10)-C(23)	121.04(18)
C(8)-C(8A)	1.431(3)	C(10A)-C(10)-C(23)	119.44(18)
C(8A)-C(9)	1.420(3)	C(5)-C(6)-C(7)	120.11(18)
C(12)-C(13)	1.385(3)	C(10)-C(10A)-C(5)	121.50(18)
C(12)-C(11)	1.391(3)	C(10)-C(10A)-C(8A)	120.37(17)
C(9)-C(9A)	1.416(3)	C(5)-C(10A)-C(8A)	118.12(18)
C(14)-C(15)	1.382(3)	C(8)-C(7)-C(6)	120.50(19)
C(14)-C(13)	1.384(3)	C(7)-C(8)-C(8A)	121.76(19)
C(23)-C(24)	1.532(3)	C(9)-C(8A)-C(8)	122.51(18)
C(15)-C(16)	1.389(3)	C(9)-C(8A)-C(10A)	119.85(17)
C(16)-C(11)	1.394(3)	C(8)-C(8A)-C(10A)	117.63(17)
C(17)-C(18)	1.393(3)	C(13)-C(12)-C(11)	119.97(18)
C(17)-C(22)	1.393(3)	C(9A)-C(9)-C(8A)	119.13(17)
C(18)-C(19)	1.391(3)	C(9A)-C(9)-P(1)	123.19(14)
C(19)-C(20)	1.387(3)	C(8A)-C(9)-P(1)	117.64(15)
C(20)-C(21)	1.382(3)	C(9)-C(9A)-C(1)	122.78(18)
C(21)-C(22)	1.390(3)	C(9)-C(9A)-C(4A)	119.76(17)
C(25)-C(26)	1.375(4)	C(1)-C(9A)-C(4A)	117.41(18)
C(26)-C(27)	1.355(4)	C(15)-C(14)-C(13)	120.03(18)
C(14)-C(13)-C(12)	120.29(19)	C(19)-C(18)-C(17)	120.1(2)
C(10)-C(23)-C(24)	110.85(17)	C(20)-C(19)-C(18)	119.9(2)
C(14)-C(15)-C(16)	120.18(19)	C(21)-C(20)-C(19)	120.3(2)
C(15)-C(16)-C(11)	119.85(19)	C(20)-C(21)-C(22)	119.9(2)
C(12)-C(11)-C(16)	119.67(18)	C(21)-C(22)-C(17)	120.3(2)
C(12)-C(11)-P(1)	120.63(14)	N(1)-C(25)-C(26)	123.6(2)
C(16)-C(11)-P(1)	119.68(15)	C(27)-C(26)-C(25)	119.5(2)
C(18)-C(17)-C(22)	119.46(19)	C(26)-C(27)-C(28)	118.0(2)

C(18)-C(17)-P(1)	121.92(16)	C(29)-C(28)-C(27)	118.9(2)
C(22)-C(17)-P(1)	118.51(16)	N(1)-C(29)-C(28)	123.9(2)

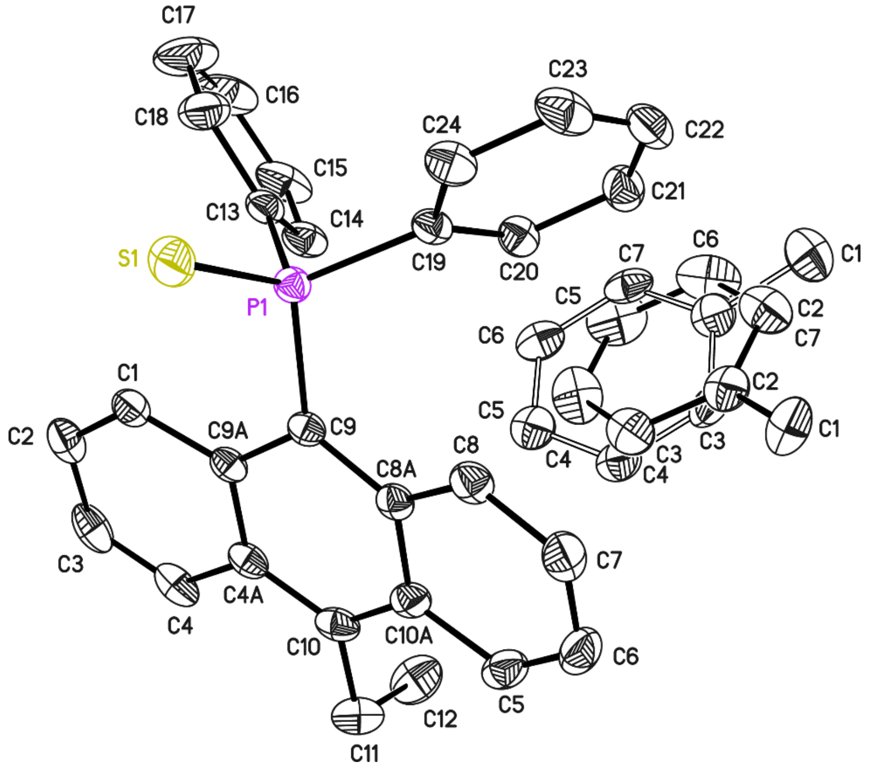

Fig. S40. Asymmetric unit of **7c** with anisotropic displacement parameters at 50 % probability level. Hydrogen atoms are omitted for clarity. The toluene molecule is disordered over two positions. It was refined with distance restraints and restraints for the anisotropic displacement parameters.[5] The occupancy of the major component refined to 0.662(4).

Table S15. Bond lengths [Å] and angles [°] for **7c**.			
C12-C22	1.489(7)	C(9)-C(9A)	1.4209(18)
C22-C72	1.379(9)	C(11)-C(12)	1.390(2)
C22-C32	1.403(8)	C(11)-C(16)	1.392(2)
C32-C42	1.379(9)	C(12)-C(13)	1.393(2)
C42-C52	1.401(9)	C(13)-C(14)	1.383(3)
C52-C62	1.382(7)	C(14)-C(15)	1.371(3)
C62-C72	1.405(8)	C(15)-C(16)	1.389(3)
C11-C21	1.491(4)	C(18)-C(19)	1.387(2)
C21-C31	1.381(5)	C(18)-C(17)	1.3919(19)
C21-C71	1.392(4)	C(19)-C(20)	1.382(2)
C31-C41	1.376(6)	C(20)-C(21)	1.378(2)
C41-C51	1.380(6)	C(21)-C(22)	1.391(2)
C51-C61	1.385(5)	C(22)-C(17)	1.3927(19)

C61-C71	1.378(4)		
P(1)-C(17)	1.8201(15)	C72-C22-C32	119.4(6)
P(1)-C(9)	1.8281(13)	C72-C22-C12	119.2(6)
P(1)-C(11)	1.8301(14)	C32-C22-C12	121.4(6)
P(1)-S(1)	1.9617(7)	C42-C32-C22	120.2(6)
C(1)-C(2)	1.364(2)	C32-C42-C52	120.6(7)
C(1)-C(9A)	1.4335(19)	C62-C52-C42	119.1(6)
C(2)-C(3)	1.412(2)	C52-C62-C72	120.4(6)
C(3)-C(4)	1.354(2)	C22-C72-C62	120.2(6)
C(4)-C(4A)	1.4356(19)	C31-C21-C71	118.5(4)
C(4A)-C(10)	1.407(2)	C31-C21-C11	120.9(3)
C(4A)-C(9A)	1.4367(19)	C71-C21-C11	120.5(3)
C(24)-C(23)	1.513(2)	C41-C31-C21	121.8(4)
C(23)-C(10)	1.533(2)	C31-C41-C51	119.5(4)
C(10)-C(10A)	1.410(2)	C41-C51-C61	119.4(4)
C(6)-C(5)	1.356(2)	C71-C61-C51	121.0(4)
C(6)-C(7)	1.408(2)	C61-C71-C21	119.8(3)
C(5)-C(10A)	1.435(2)	C(17)-P(1)-C(9)	110.40(6)
C(10A)-C(8A)	1.4415(19)	C(17)-P(1)-C(11)	100.37(6)
C(7)-C(8)	1.363(2)	C(9)-P(1)-C(11)	105.25(6)
C(8)-C(8A)	1.4337(19)	C(17)-P(1)-S(1)	112.27(5)
C(8A)-C(9)	1.4158(18)	C(9)-P(1)-S(1)	113.96(5)
C(11)-P(1)-S(1)	113.59(5)	C(8A)-C(9)-C(9A)	119.45(12)
C(2)-C(1)-C(9A)	121.77(13)	C(8A)-C(9)-P(1)	122.78(10)
C(1)-C(2)-C(3)	120.20(14)	C(9A)-C(9)-P(1)	117.77(10)
C(4)-C(3)-C(2)	120.09(13)	C(9)-C(9A)-C(1)	122.63(12)
C(3)-C(4)-C(4A)	122.08(14)	C(9)-C(9A)-C(4A)	119.56(12)
C(10)-C(4A)-C(4)	121.68(13)	C(1)-C(9A)-C(4A)	117.80(12)
C(10)-C(4A)-C(9A)	120.51(12)	C(12)-C(11)-C(16)	119.49(14)
C(4)-C(4A)-C(9A)	117.81(13)	C(12)-C(11)-P(1)	122.32(11)
C(24)-C(23)-C(10)	111.26(13)	C(16)-C(11)-P(1)	118.15(12)
C(4A)-C(10)-C(10A)	119.68(12)	C(11)-C(12)-C(13)	119.95(15)
C(4A)-C(10)-C(23)	119.61(13)	C(14)-C(13)-C(12)	119.84(17)
C(10A)-C(10)-C(23)	120.70(13)	C(15)-C(14)-C(13)	120.52(16)
C(5)-C(6)-C(7)	120.24(14)	C(14)-C(15)-C(16)	120.12(17)

C(6)-C(5)-C(10A)	121.87(14)	C(15)-C(16)-C(11)	120.05(17)
C(10)-C(10A)-C(5)	121.76(13)	C(19)-C(18)-C(17)	119.89(13)
C(10)-C(10A)-C(8A)	120.32(12)	C(20)-C(19)-C(18)	120.30(14)
C(5)-C(10A)-C(8A)	117.92(13)	C(21)-C(20)-C(19)	120.15(14)
C(8)-C(7)-C(6)	120.32(14)	C(20)-C(21)-C(22)	120.16(14)
C(7)-C(8)-C(8A)	121.84(14)	C(21)-C(22)-C(17)	119.93(14)
C(9)-C(8A)-C(8)	122.88(12)	C(18)-C(17)-C(22)	119.58(13)
C(9)-C(8A)-C(10A)	119.55(12)	C(18)-C(17)-P(1)	120.90(10)
C(8)-C(8A)-C(10A)	117.51(12)	C(22)-C(17)-P(1)	119.47(11)

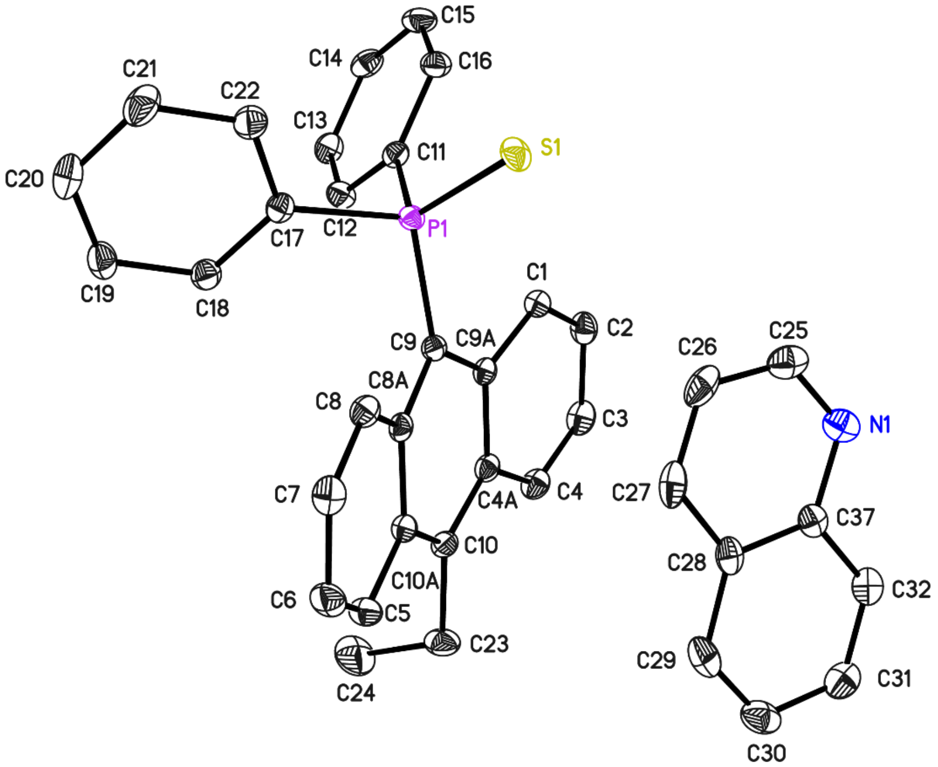

Fig. S 41. Asymmetric unit of **7d** with anisotropic displacement parameters at 50 % probability level. Hydrogen atoms are omitted for clarity.

Table 16. Bond lengths [Å] and angles [°] for **7d**.			
S(1)-P(1)	1.9559(5)	C(29)-C(30)	1.367(2)
C(1)-C(2)	1.3607(17)	C(29)-C(28)	1.4131(19)
C(1)-C(9A)	1.4301(16)	C(31)-C(30)	1.414(2)
P(1)-C(11)	1.8227(13)	C(28)-C(37)	1.4201(17)
P(1)-C(9)	1.8247(12)	C(28)-C(27)	1.4216(19)
P(1)-C(17)	1.8262(13)	C(27)-C(26)	1.363(2)

N(1)-C(25)	1.3163(18)	C(26)-C(25)	1.406(2)
N(1)-C(37)	1.3697(17)		
C(2)-C(3)	1.4174(19)	C(2)-C(1)-C(9A)	122.06(11)
C(3)-C(4)	1.3584(18)	C(11)-P(1)-C(9)	107.47(5)
C(4)-C(4A)	1.4349(17)	C(11)-P(1)-C(17)	98.47(5)
C(4A)-C(10)	1.4108(17)	C(9)-P(1)-C(17)	107.15(5)
C(4A)-C(9A)	1.4424(16)	C(11)-P(1)-S(1)	113.84(4)
C(5)-C(6)	1.3598(19)	C(9)-P(1)-S(1)	114.51(4)
C(5)-C(10A)	1.4361(17)	C(17)-P(1)-S(1)	113.99(4)
C(10)-C(10A)	1.4108(17)	C(25)-N(1)-C(37)	117.04(12)
C(10)-C(23)	1.5153(17)	C(1)-C(2)-C(3)	120.06(12)
C(6)-C(7)	1.4144(19)	C(4)-C(3)-C(2)	120.27(11)
C(10A)-C(8A)	1.4413(16)	C(3)-C(4)-C(4A)	121.68(12)
C(7)-C(8)	1.3633(17)	C(10)-C(4A)-C(4)	121.19(11)
C(8)-C(8A)	1.4318(16)	C(10)-C(4A)-C(9A)	120.73(11)
C(8A)-C(9)	1.4169(16)	C(4)-C(4A)-C(9A)	118.02(11)
C(12)-C(13)	1.3864(17)	C(6)-C(5)-C(10A)	121.92(12)
C(12)-C(11)	1.3958(17)	C(10A)-C(10)-C(4A)	118.97(11)
C(9)-C(9A)	1.4174(16)	C(10A)-C(10)-C(23)	120.64(11)
C(14)-C(15)	1.3872(19)	C(4A)-C(10)-C(23)	120.37(11)
C(14)-C(13)	1.3902(18)	C(5)-C(6)-C(7)	120.21(11)
C(23)-C(24)	1.530(2)	C(10)-C(10A)-C(5)	121.63(11)
C(15)-C(16)	1.3935(18)	C(10)-C(10A)-C(8A)	120.64(11)
C(16)-C(11)	1.3906(17)	C(5)-C(10A)-C(8A)	117.70(11)
C(17)-C(22)	1.3905(17)	C(8)-C(7)-C(6)	120.15(12)
C(17)-C(18)	1.3973(17)	C(7)-C(8)-C(8A)	121.81(11)
C(18)-C(19)	1.3857(18)	C(9)-C(8A)-C(8)	122.34(11)
C(19)-C(20)	1.3895(19)	C(9)-C(8A)-C(10A)	119.56(10)
C(20)-C(21)	1.383(2)	C(8)-C(8A)-C(10A)	118.05(10)
C(21)-C(22)	1.3959(18)	C(13)-C(12)-C(11)	120.28(11)
C(32)-C(31)	1.366(2)	C(8A)-C(9)-C(9A)	119.28(10)
C(32)-C(37)	1.4170(18)	C(8A)-C(9)-P(1)	119.22(9)
C(9A)-C(9)-P(1)	121.44(9)	C(21)-C(20)-C(19)	120.17(12)
C(9)-C(9A)-C(1)	122.67(11)	C(20)-C(21)-C(22)	120.32(12)
C(9)-C(9A)-C(4A)	119.43(10)	C(17)-C(22)-C(21)	119.65(12)

C(1)-C(9A)-C(4A)	117.74(10)	C(31)-C(32)-C(37)	120.15(12)
C(15)-C(14)-C(13)	120.18(12)	C(30)-C(29)-C(28)	120.35(12)
C(12)-C(13)-C(14)	119.82(12)	C(32)-C(31)-C(30)	120.84(13)
C(10)-C(23)-C(24)	112.37(11)	C(29)-C(30)-C(31)	120.20(13)
C(14)-C(15)-C(16)	120.12(12)	C(29)-C(28)-C(37)	119.45(12)
C(11)-C(16)-C(15)	119.85(12)	C(29)-C(28)-C(27)	123.38(12)
C(16)-C(11)-C(12)	119.76(11)	C(37)-C(28)-C(27)	117.17(12)
C(16)-C(11)-P(1)	119.65(9)	C(26)-C(27)-C(28)	119.46(13)
C(12)-C(11)-P(1)	120.50(9)	C(27)-C(26)-C(25)	118.71(13)
C(22)-C(17)-C(18)	119.71(11)	N(1)-C(25)-C(26)	124.78(13)
C(22)-C(17)-P(1)	118.32(9)	N(1)-C(37)-C(32)	118.19(11)
C(18)-C(17)-P(1)	121.72(9)	N(1)-C(37)-C(28)	122.81(12)
C(19)-C(18)-C(17)	120.34(12)	C(32)-C(37)-C(28)	119.00(12)
C(18)-C(19)-C(20)	119.81(12)		

S3. Photophysical data

Table S17. Photophysical data of **1** – **8** in diluted THF solution (10^{-5} M).

	λ_{abs} ($S_0 \rightarrow S_1$) / nm	λ_{em}[a] / nm	τ[b] / ns	Φ_F[a] / %
**1**	340 / 358 / 377 / 397	-	-	-
**2**	346 / 367 / 385 / 406	-	-	-
**3**	348 / 368 / 386 / 406	-	-	-
**4**	351 / 370 / 388 / 410	-	-	-
**5**	352 / 371 / 392 / 411	462	9.3	< 1.0
**6**	357 / 376 / 400 / 420	480	1.4	6.7 ± 0.4
**7**	358 / /375 / 400 / 421	477	1.8	7.6 ± 0.2
**8**	355 / 373 / 400 / 421	472	0.5	2.4 ± 0.1

[a] λ_{ex} = 375 nm; [b] λ_{ex} = 375 nm, detection at λ_{em}.

Table S18. Full widths at half maximum (FWHM) of the emission spectra of **5** – **8** in diluted THF solution (10^{-5} M) and in the solid-state.

	FWHM in THF / nm	FWHM in the solid-state / nm
**5**	80	56
**6**	87	63
**7**	87	71
**7a**	-	59
**7b**	-	67
**7c**	-	63
**7d**	-	74
**8**	81	61

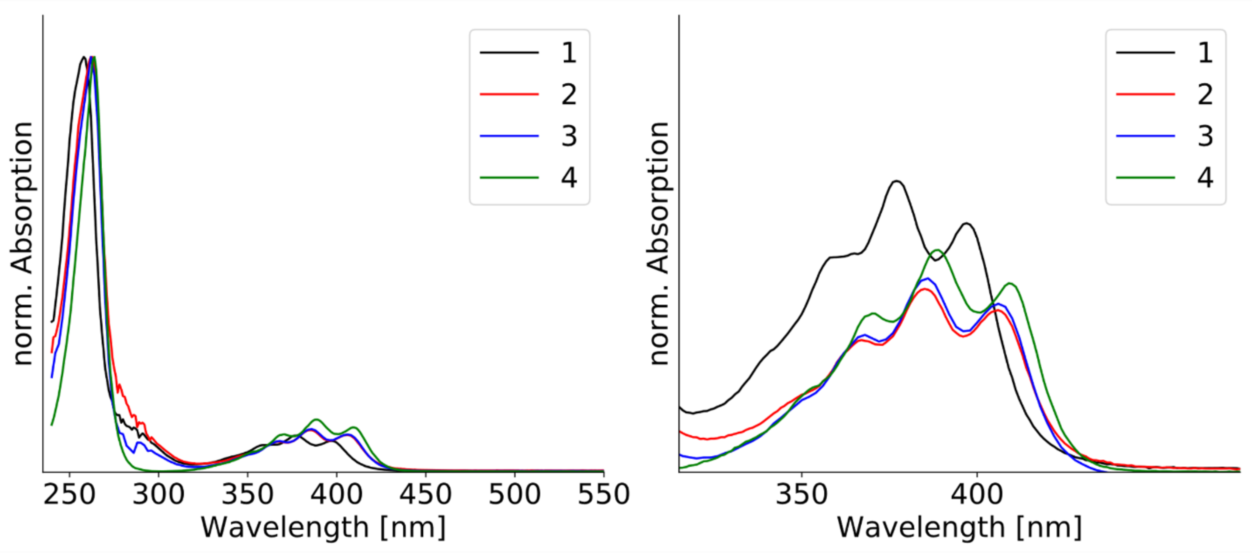

Fig. S42. UV/VIS spectra of [9-PPh$_2$-($C_{14}H_9$) (**1**), [9-PPh$_2$-10-Me-($C_{14}H_8$) (**2**), [9-PPh$_2$-10-Et-($C_{14}H_8$) (**3**), [9-PPh$_2$-10-Ph-($C_{14}H_8$) (**4**) in diluted THF-solution (10^{-5} M). The right figure shows an excerpt of the region around 320 – 475 nm.

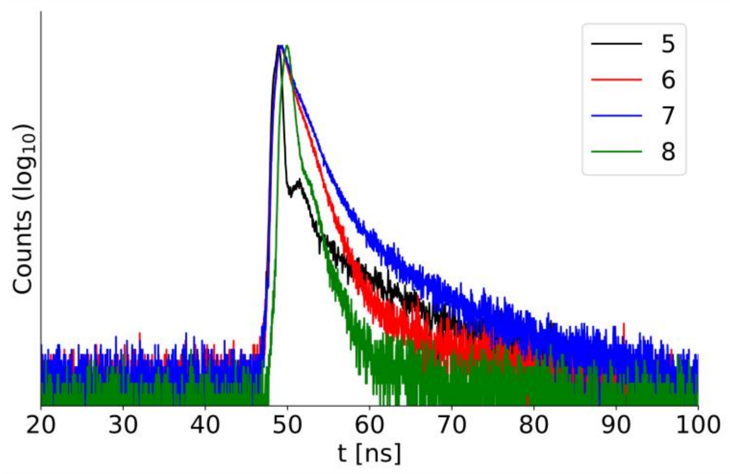

Fig. S43. Fluorescence lifetime decay plots of thiophosphoranyl anthracenes (**5**-**8**) in THF solution (10^{-5} M, λ_{ex} = 375 nm).

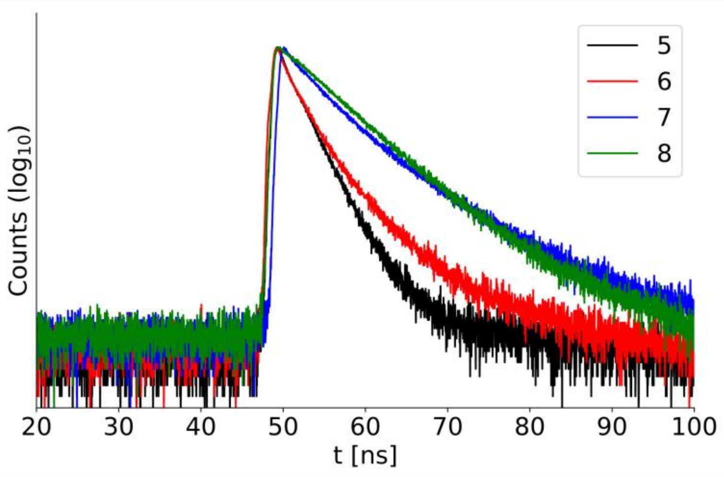

Fig. S44. Fluorescence lifetime decay plots of thiophosphoranyl anthracenes (**5**-**8**) in the solid-state (λ_{ex} = 375 nm).

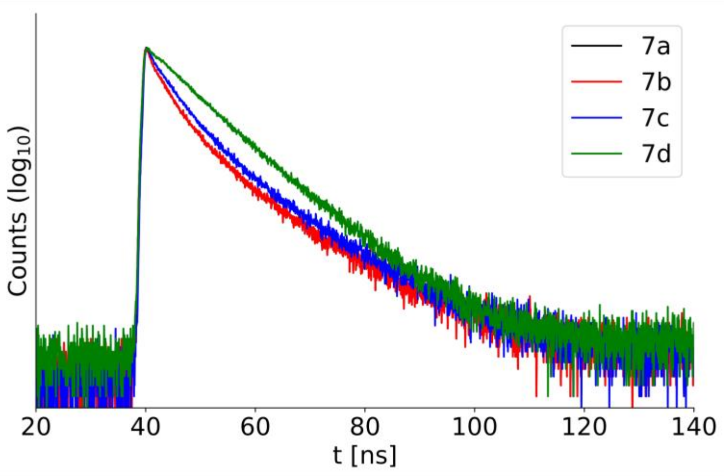

Fig. S45. Fluorescence lifetime decay plots of co-crystals (**7a**-**7d**) the solid-state (λ_{ex} = 375 nm).

S4. Computations

Theoretical geometry optimizations were carried out at the D3-B3LYP//def2-TZVP level. Starting points for the optimizations were the solid-state structures. The optimised structures can be found in Figure S46. Additional geometry optimizations were conducted starting from the respective other geometry by removing or adding the sulfur atom from the calculation respectively. At the TZVP level, these calculations did not yield the same minimum structures but resulted in alternatives of higher energy, which therefore do not represent the global minimum. These are depicted in Figure 47. The energy difference between the solid-state structure and the one derived from the other compound is 16.364 kJ/mol for 1 and 13.983 kJ/mol for 5 respectively. These structures therefore represent only a local minimum.

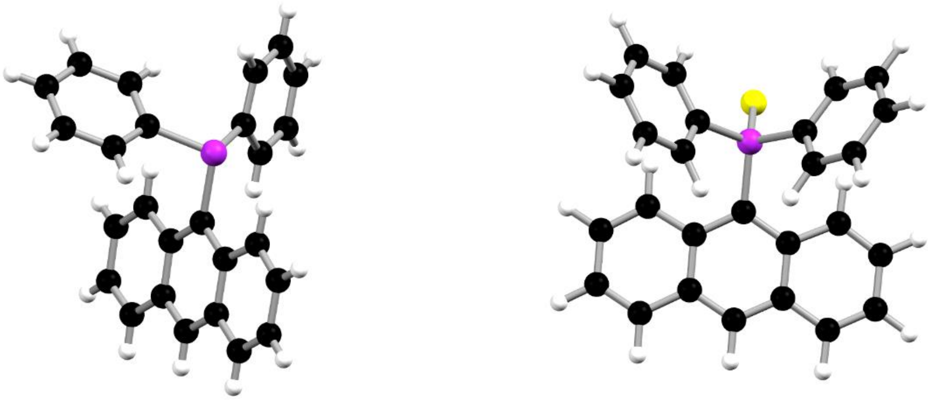

Fig. S 46. Optimised structures starting from the solid-state structures. Geometry optimizations were carried out at the D3-B3LYP//def2-TZVP level.

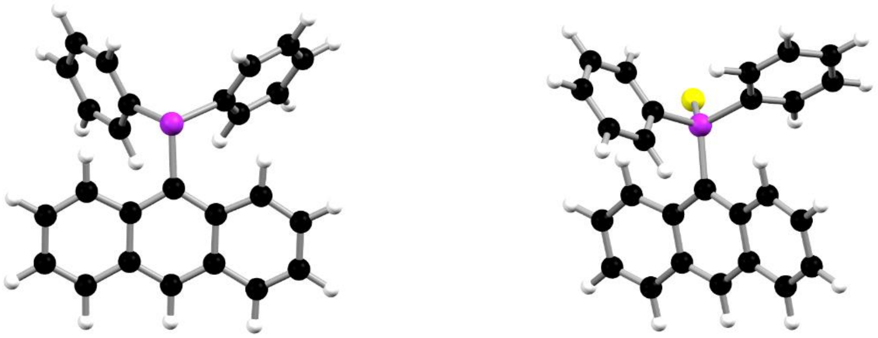

Fig. S 47. Optimised structures starting from the solid-state structures of the respective other compound by adding or removing a sulfur atom. Geometry optimizations were carried out at the D3-B3LYP//def2-TZVP level.

Table S19. Coordinates of the optimised structure **1.**			
P	8.78826	9.59222	11.29138

C	6.267	9.89257	9.23397
H	7.05033	10.62678	9.30926
C	5.21382	10.13823	8.40199
H	5.1822	11.06455	7.84229
C	4.16247	9.19825	8.25727
H	3.33243	9.41436	7.59678
C	4.21214	8.02451	8.9458
H	3.42464	7.28766	8.83985
C	6.45857	4.92487	11.99484
H	5.64773	4.22707	11.82184
C	7.49106	4.59889	12.8198
H	7.5166	3.63688	13.31562
C	8.53957	5.53099	13.03166
H	9.35832	5.27004	13.69059
C	8.53063	6.75058	12.42086
H	9.33726	7.44496	12.6041
C	7.43367	8.39641	10.87466
C	5.35579	6.51195	10.49237
H	4.55488	5.79424	10.35126
C	9.70644	9.85762	9.72349
C	9.44467	9.17502	8.53267
H	8.60393	8.4987	8.46981
C	10.25682	9.35485	7.41803
H	10.03605	8.81551	6.50517
C	11.34008	10.22338	7.46916
H	11.96778	10.36514	6.59851
C	11.62062	10.89928	8.65262
H	12.47147	11.56683	8.70964
C	10.82219	10.70497	9.77138
H	11.073	11.21123	10.69637
C	7.87243	11.16147	11.60222
C	6.86953	11.12489	12.57795
H	6.61557	10.18439	13.0529
C	6.18058	12.27534	12.93422
H	5.39968	12.22161	13.6827
C	6.49196	13.49268	12.33593
H	5.95747	14.39147	12.61601
C	7.49454	13.54281	11.37566
H	7.74367	14.48331	10.89944
C	8.17885	12.38813	11.00992
H	8.93913	12.44803	10.24276
C	5.30064	7.72745	9.81765
C	7.4748	7.14387	11.53873
C	6.36186	8.68761	9.99386
C	6.41363	6.18762	11.33307

Table S20. Coordinates of the optimised structure **1** after addition of a Sulfur atom at the Phosphorous.			
P	8.44391	9.77169	11.48908
S	9.05224	9.53577	13.33873
C	5.67256	9.94679	9.63797
H	6.4034	10.73002	9.55224
C	4.44667	10.14788	9.07277
H	4.24146	11.08752	8.57548
C	3.44661	9.14513	9.1141
H	2.47351	9.33151	8.67836
C	3.73814	7.93952	9.67411
H	3.00559	7.14112	9.68046
C	6.93343	4.7433	11.51074
H	6.1532	3.99392	11.45077
C	8.19525	4.40646	11.89446
H	8.43776	3.38418	12.15513
C	9.20152	5.40323	11.93189
H	10.21299	5.12946	12.20408
C	8.92009	6.70371	11.62814
H	9.7197	7.42406	11.67011
C	7.26389	8.45622	10.91371
C	5.33135	6.40613	10.70758
H	4.5697	5.63454	10.68455
C	9.77206	9.7739	10.23231
C	9.4973	9.44406	8.90381
H	8.49805	9.14425	8.61424
C	10.50796	9.48645	7.95139
H	10.29004	9.22451	6.92375
C	11.79737	9.85619	8.32032
H	12.58553	9.88393	7.57836
C	12.07536	10.18076	9.64404
H	13.07961	10.46197	9.93493
C	11.06674	10.13944	10.59918
H	11.27612	10.3765	11.63463
C	7.67075	11.42299	11.35843
C	6.62869	11.73261	12.23646
H	6.28778	10.99	12.9458
C	6.04047	12.98701	12.2056
H	5.22632	13.21452	12.8819
C	6.49979	13.95419	11.31448
H	6.04334	14.93582	11.29644
C	7.55203	13.65928	10.4575
H	7.92348	14.41102	9.77247
C	8.13597	12.39616	10.47493
H	8.94741	12.17434	9.7955
C	5.02173	7.67579	10.23712
C	7.60828	7.1171	11.24236

C	6.01702	8.72258	10.29068
C	6.60841	6.08434	11.15268

Table S21: Coordinates of the optimised structure **5.**			
C	4.86606	8.29389	7.59362
H	5.32736	8.88175	6.81601
S	3.73209	10.94363	4.80944
P	3.33549	9.03028	4.90195
C	5.63835	7.84033	8.62283
H	6.69945	8.05484	8.6266
C	5.06978	7.10933	9.69674
H	5.70335	6.74481	10.49513
C	3.72427	6.90277	9.73223
H	3.26629	6.38147	10.56442
C	-0.76802	7.77778	7.99629
H	-1.16745	7.2453	8.85125
C	-1.59806	8.40767	7.11954
H	-2.67111	8.37544	7.25823
C	-1.0483	9.14179	6.038
H	-1.70673	9.69057	5.37671
C	0.29919	9.18261	5.82821
H	0.68283	9.78528	5.02024
C	2.61403	8.4868	6.50274
C	1.49547	7.25277	8.7755
H	1.06652	6.72109	9.61771
C	4.77951	7.93831	4.57127
C	4.74549	6.57015	4.85002
H	3.88825	6.13423	5.34461
C	5.82532	5.76316	4.52363
H	5.79364	4.70549	4.75288
C	6.94819	6.31267	3.91113
H	7.79096	5.68174	3.65807
C	6.98541	7.67278	3.631
H	7.85693	8.10716	3.15763
C	5.90538	8.485	3.96116
H	5.93352	9.54767	3.75657
C	2.20797	8.42292	3.5802
C	2.01697	9.214	2.45032
H	2.47798	10.19241	2.40454
C	1.23105	8.75342	1.39901
H	1.08766	9.37622	0.525
C	0.6295	7.50336	1.47175
H	0.01406	7.1473	0.65516
C	0.81562	6.71108	2.60111
H	0.34199	5.73979	2.6682

C	1.60223	7.1663	3.64895
H	1.72062	6.54972	4.52948
C	3.45776	8.06124	7.5575
C	2.87732	7.39343	8.69529
C	0.64777	7.82777	7.83394
C	1.21019	8.49892	6.68923

Table S22. Coordinates of the optimised structure **5** after the Sulfur at the Phosphorous was removed.			
C	4.84298	8.02716	7.56803
H	5.35372	8.56121	6.78483
P	3.27633	9.09545	4.8956
C	5.58669	7.49166	8.57879
H	6.66357	7.60274	8.56409
C	4.9686	6.80199	9.65202
H	5.57562	6.36672	10.43564
C	3.61056	6.72136	9.7023
H	3.11699	6.23154	10.53346
C	-0.80799	8.02509	8.06425
H	-1.23801	7.5243	8.9237
C	-1.59692	8.72369	7.20164
H	-2.66668	8.7807	7.35761
C	-1.00594	9.40296	6.10634
H	-1.62739	9.99472	5.44625
C	0.33702	9.32682	5.8786
H	0.74962	9.87205	5.04332
C	2.6029	8.45517	6.50675
C	1.41037	7.29081	8.80004
H	0.95182	6.79217	9.64666
C	4.77537	8.07628	4.52611
C	4.81655	6.68282	4.62568
H	3.95109	6.14208	4.9842
C	5.96431	5.98476	4.2828
H	5.98413	4.90562	4.37163
C	7.08948	6.66735	3.82502
H	7.98436	6.11996	3.55662
C	7.05755	8.05074	3.71311
H	7.9267	8.5887	3.35553
C	5.90576	8.75046	4.06378
H	5.88629	9.83072	3.9829
C	2.17748	8.29451	3.6351
C	2.08624	8.93579	2.3985
H	2.59994	9.87792	2.24598
C	1.34441	8.38085	1.3609
H	1.28657	8.89186	0.40783

C	0.67308	7.17953	1.55127
H	0.08897	6.74829	0.74802
C	0.74904	6.53724	2.78364
H	0.22262	5.60406	2.94224
C	1.49532	7.08898	3.81598
H	1.53004	6.58624	4.77327
C	3.4169	7.9324	7.54378
C	2.79606	7.30443	8.6867
C	0.60427	7.95224	7.88
C	1.20673	8.57934	6.73142

S5. NMR Spectra

[9-$PPh_2(C_{14}H_9)$] (1)

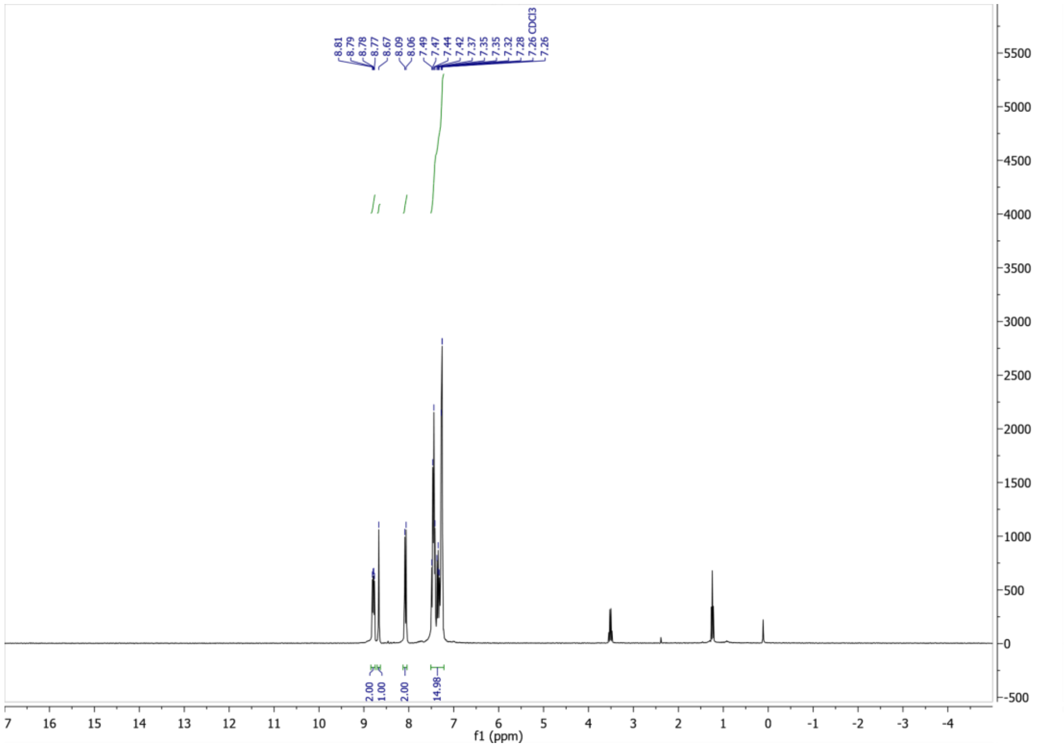

Fig. S48 1H-NMR spectrum of [9-$PPh_2(C_{14}H_9)$] (**1**) in $CDCl_3$.

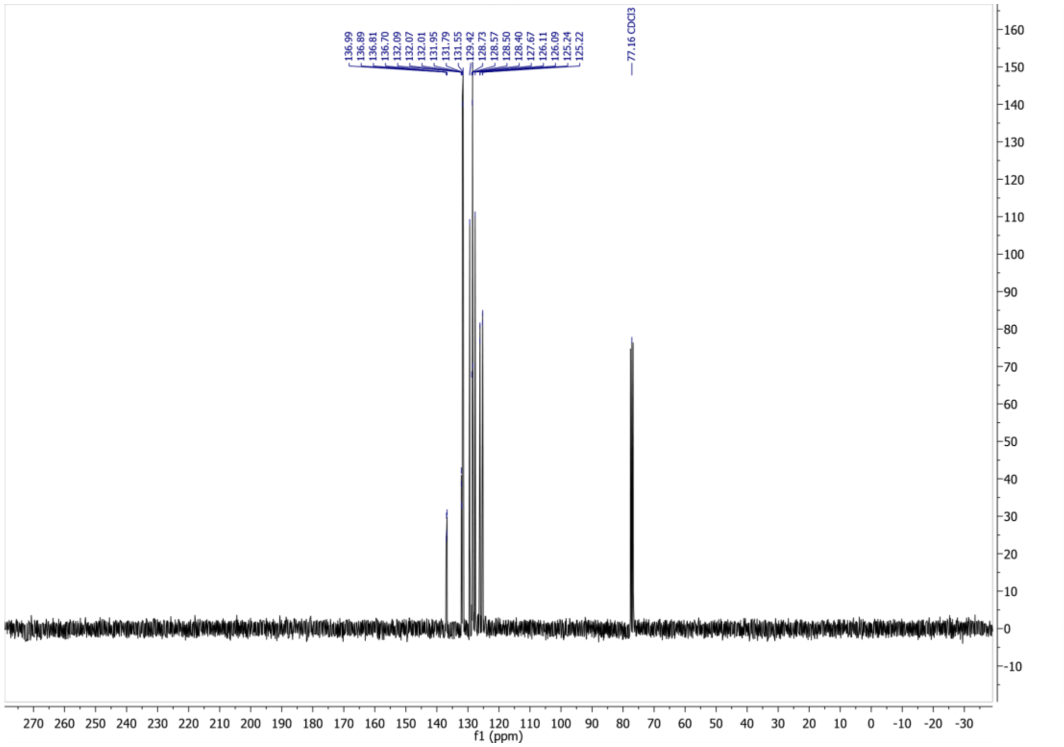

Fig. S49 13C{1H}-NMR spectrum of [9-$PPh_2(C_{14}H_9)$] (**1**) in $CDCl_3$.

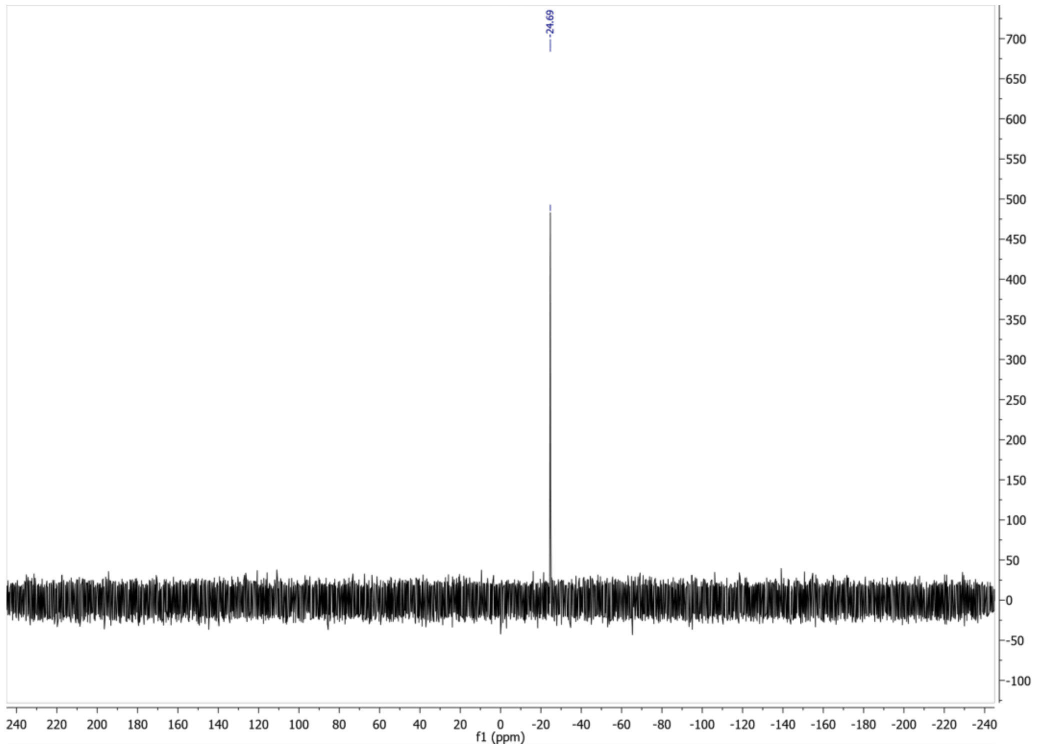

Fig. S50 31P{1H}-NMR spectrum of [9-$PPh_2(C_{14}H_9)$] (**1**) in $CDCl_3$.

[9-PPh_2-10-Me-($C_{14}H_8$)] (**2**)

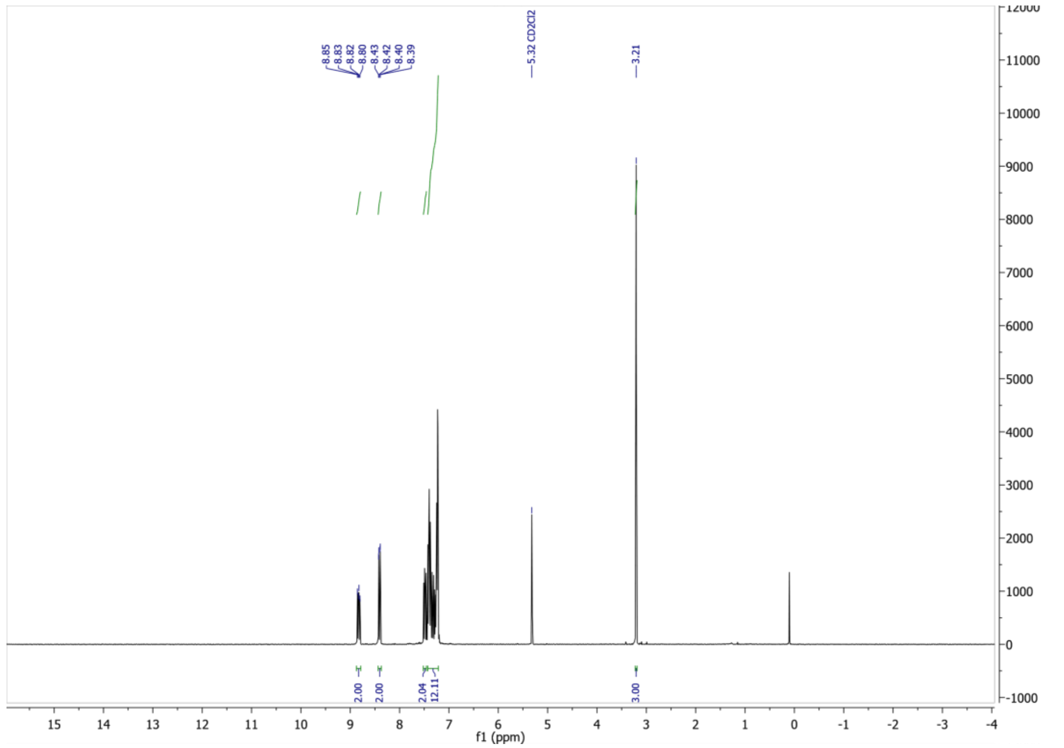

Fig. S51 1H-NMR spectrum of [9-PPh_2-10-Me-($C_{14}H_8$)] (**2**) in CD_2Cl_2.

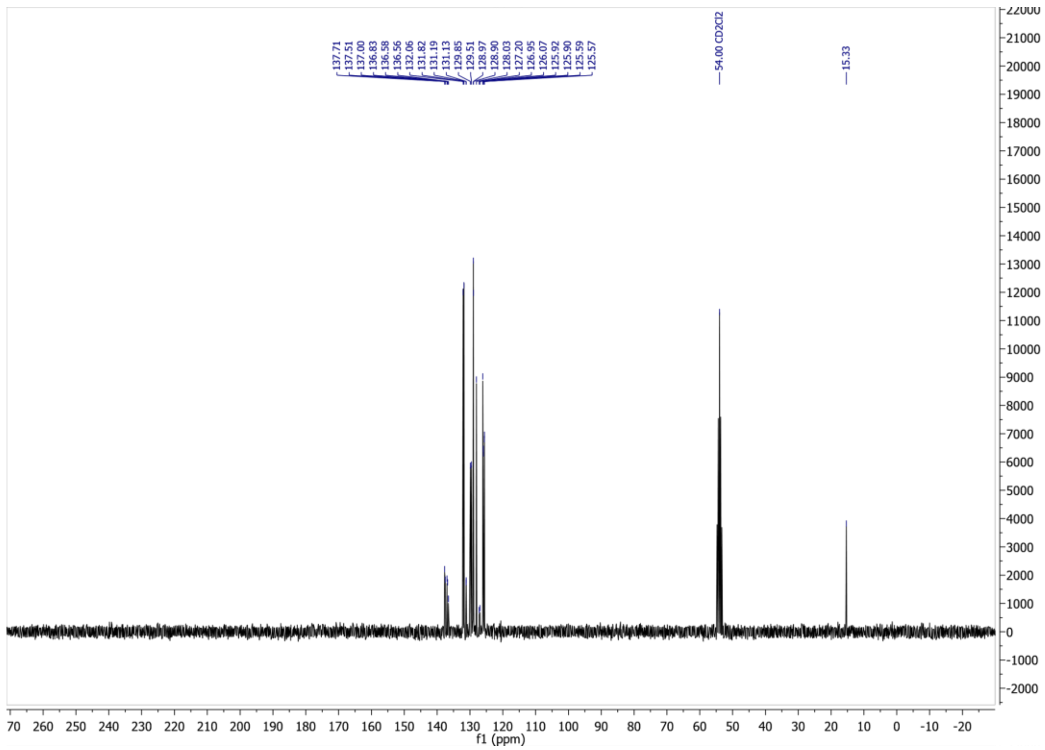

Fig. S52 13C{1H}-NMR spectrum of [9-PPh_2-10-Me-($C_{14}H_8$)] (**2**) in CD_2Cl_2.

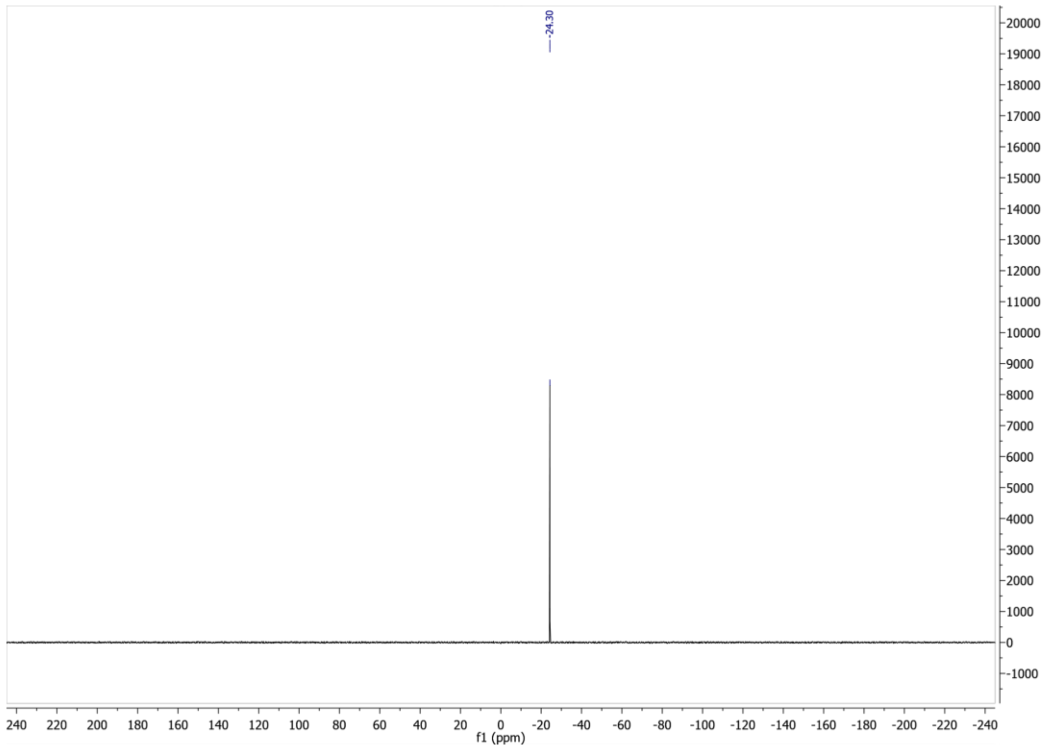

Fig. S53 31P{1H} spectrum of [9-PPh_2-10-Me-($C_{14}H_8$)] (**2**) in CD_2Cl_2.

[9-PPh$_2$-10-Et-($C_{14}H_8$)] (**3**)

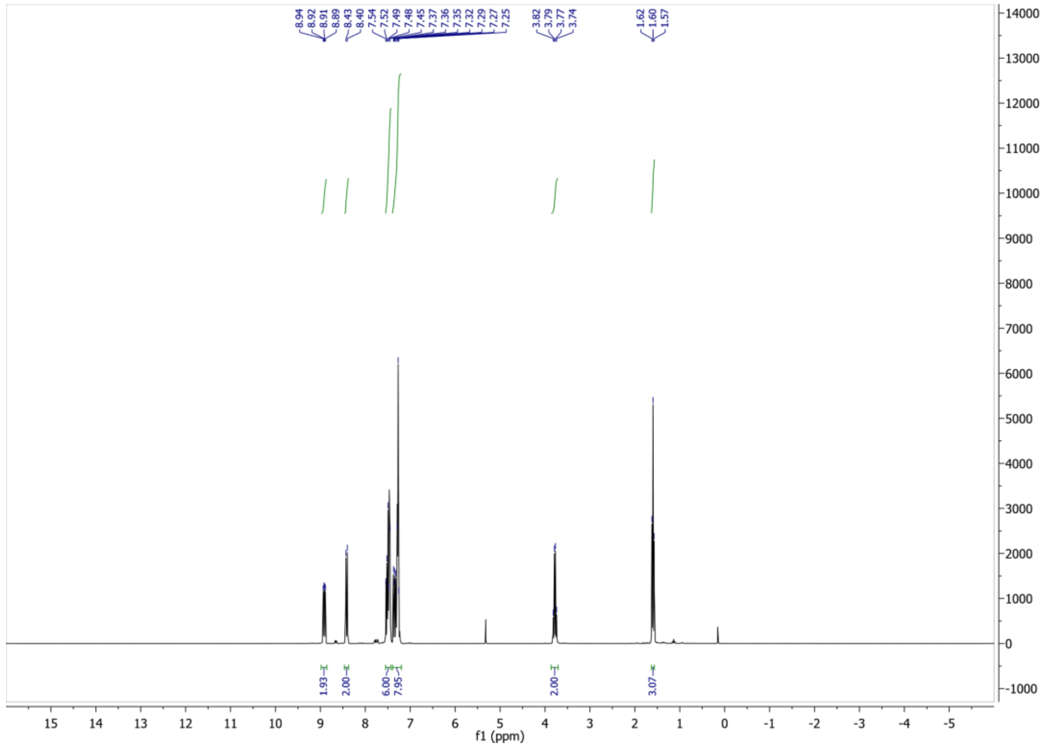

Fig. S54 1H-NMR spectrum of [9-PPh$_2$-10-Et-($C_{14}H_8$)] (**3**) in $CDCl_3$. The signal at 5.3 ppm belongs to DCM which was used for crystallisation.

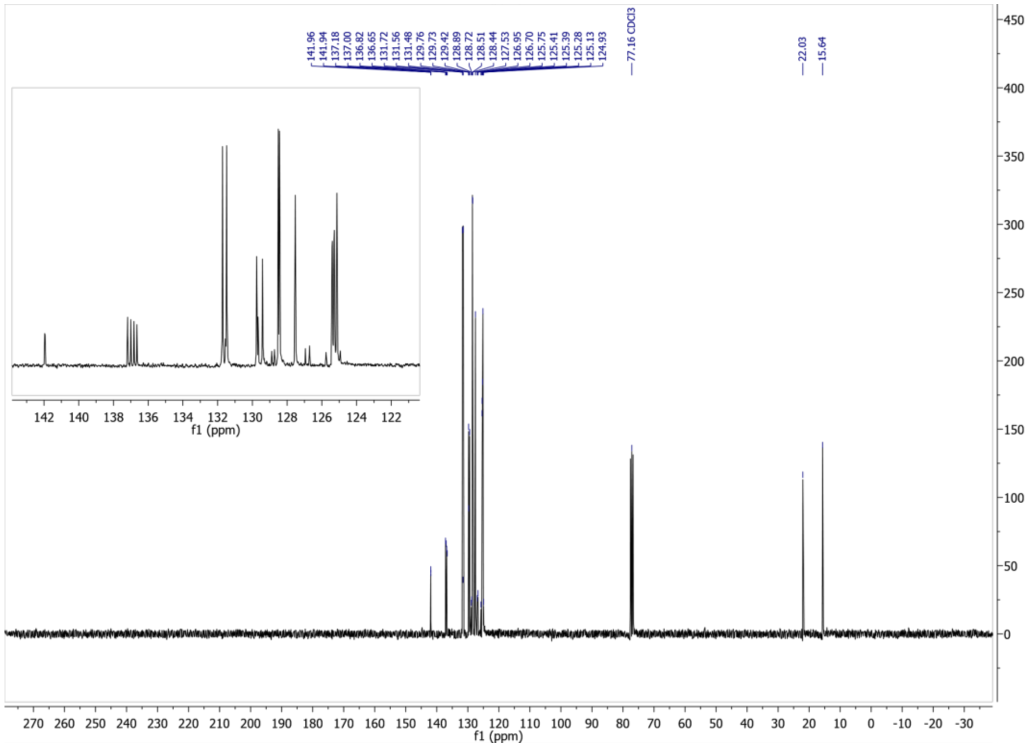

Fig. S55 13C{1H}-NMR spectrum of [9-PPh$_2$-10-Et-($C_{14}H_8$)] (**3**) in $CDCl_3$.

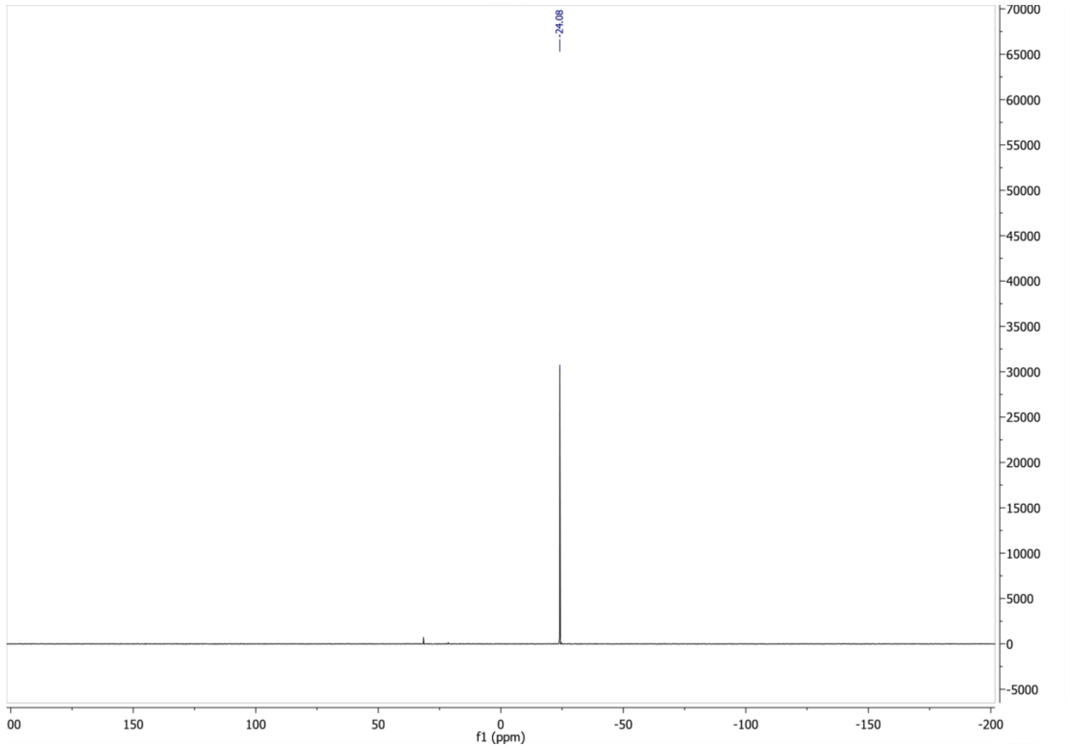

Fig. S56 31P{1H}-NMR spectrum of [9-PPh$_2$-10-Et-($C_{14}H_8$)] (**3**) in $CDCl_3$.

9-PPh$_2$-10-Ph-($C_{14}H_8$)] (**4**)

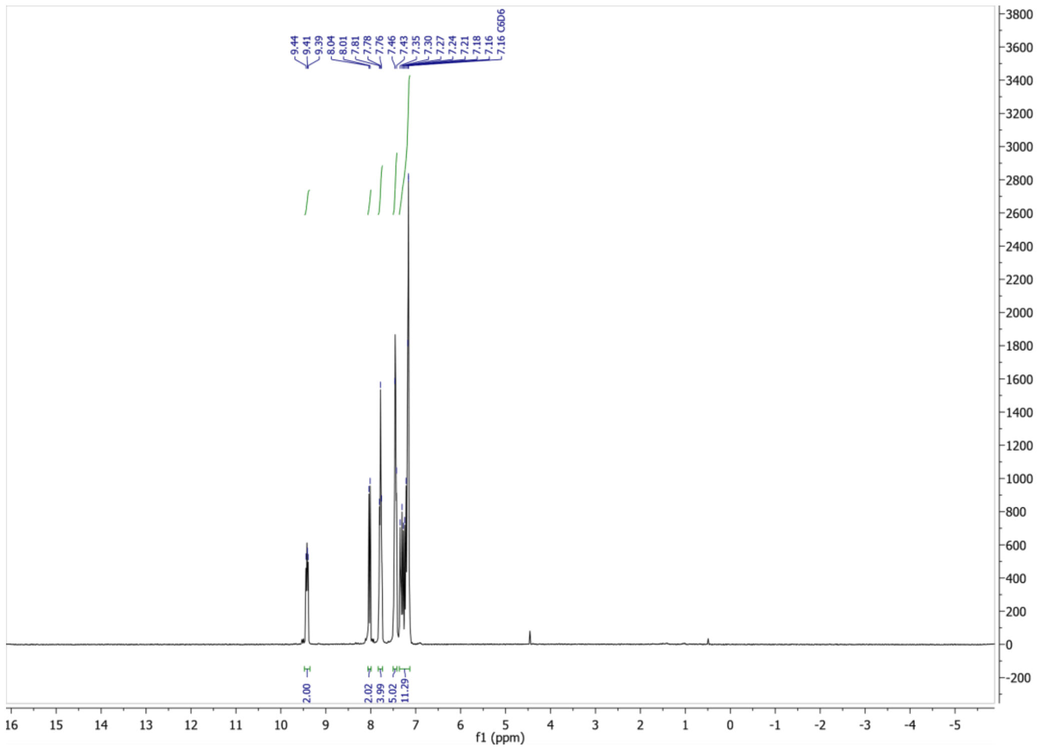

Fig. S57 1H-NMR spectrum of [9-PPh$_2$-10-Ph-($C_{14}H_8$)] (**4**) in C_6D_6.

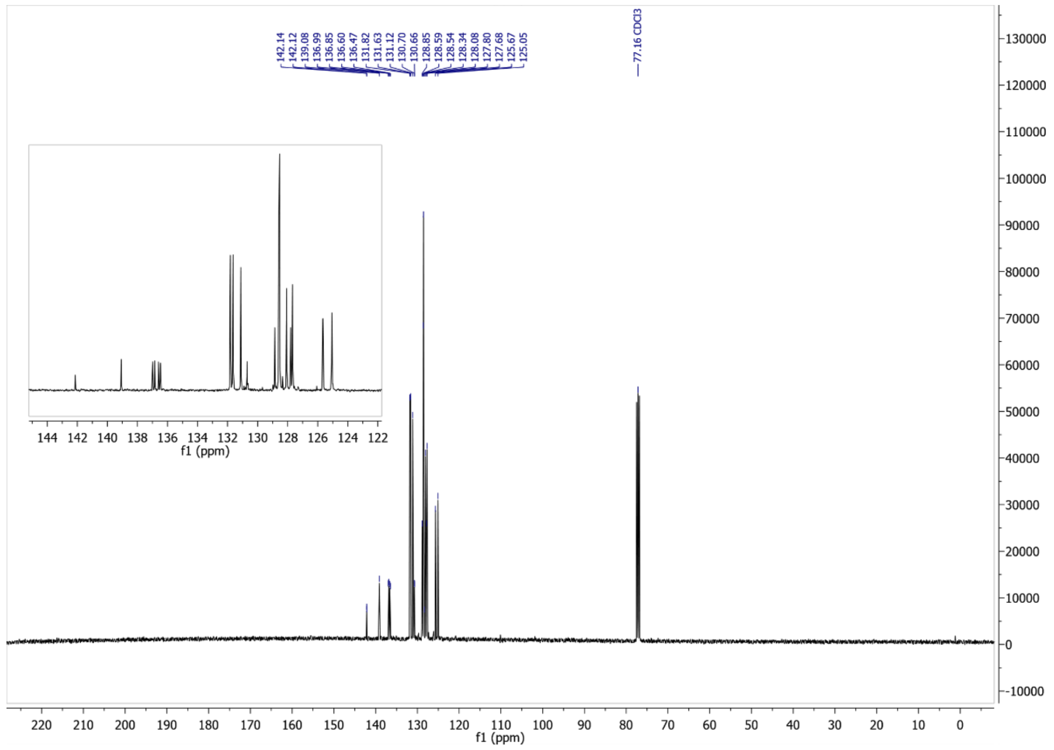

Fig. S58 13C{1H}-NMR spectrum of [9-PPh_2-10-Ph-($C_{14}H_8$)] (**4**) in $CDCl_3$.

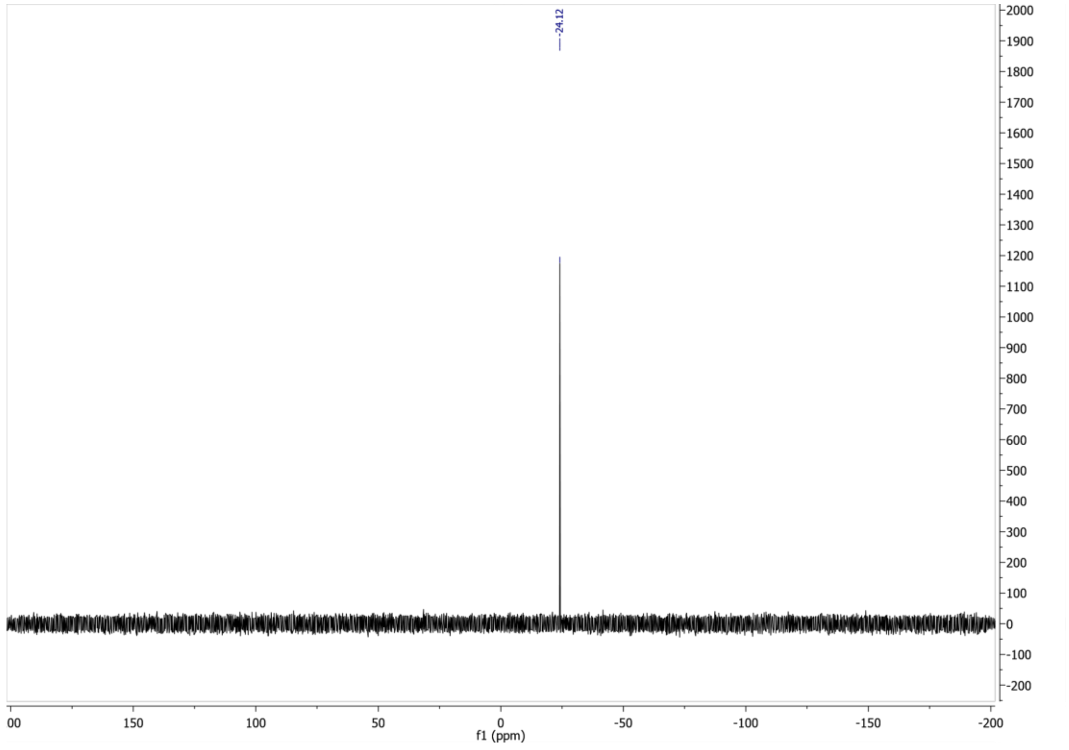

Fig. S59. 31P{1H}-NMR spectrum of [9-PPh_2-10-Ph-($C_{14}H_8$)] (**4**) in $CDCl_3$.

[9-(S)PPh$_2$($C_{14}H_9$)] (**5**)

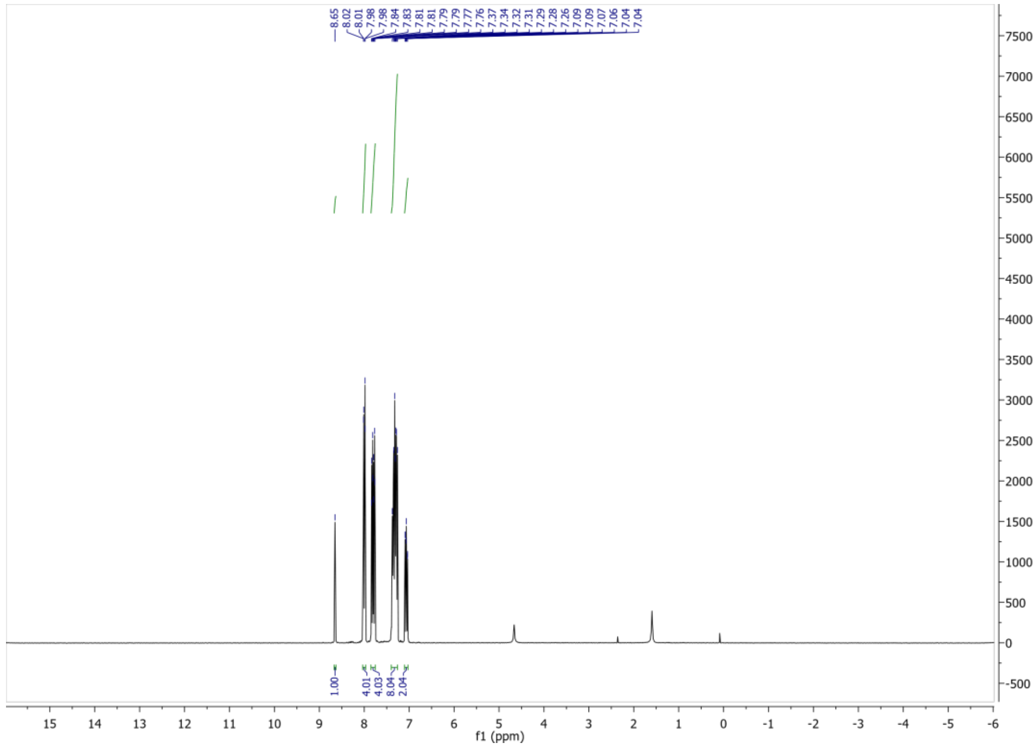

Fig. S60. 1H-NMR spectrum of [9-(S)PPh$_2$ ($C_{14}H_9$)] (**5**) in $CDCl_3$.

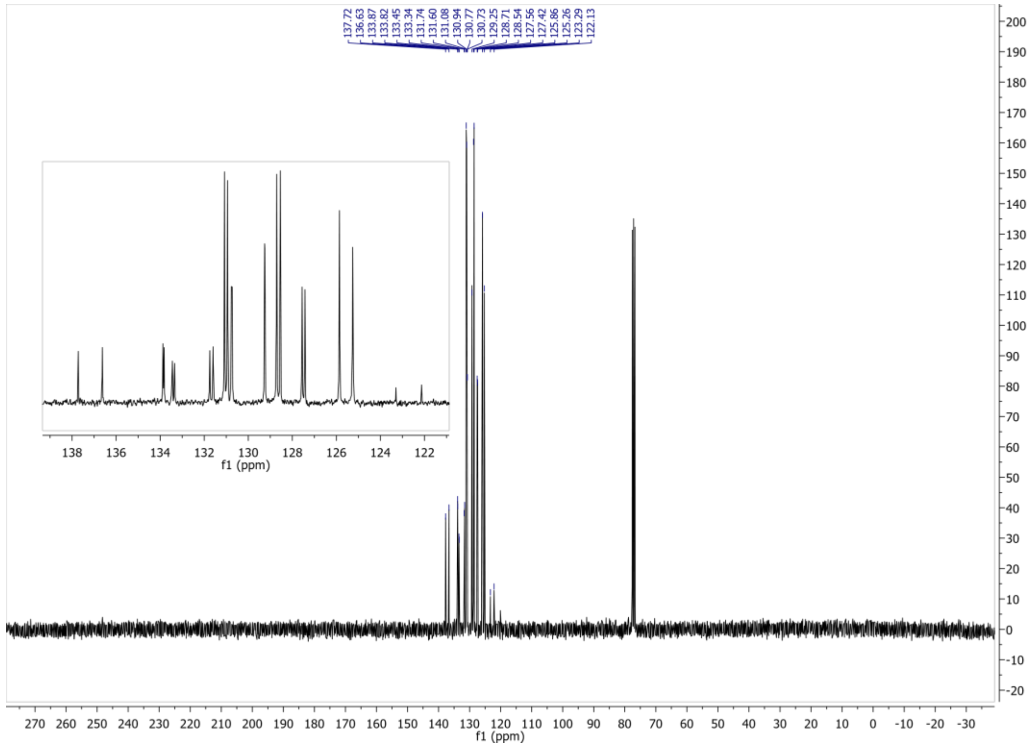

Fig. S61. 13C{1H}-NMR spectrum of [9-(S)PPh$_2$($C_{14}H_9$)] (**5**) in $CDCl_3$.

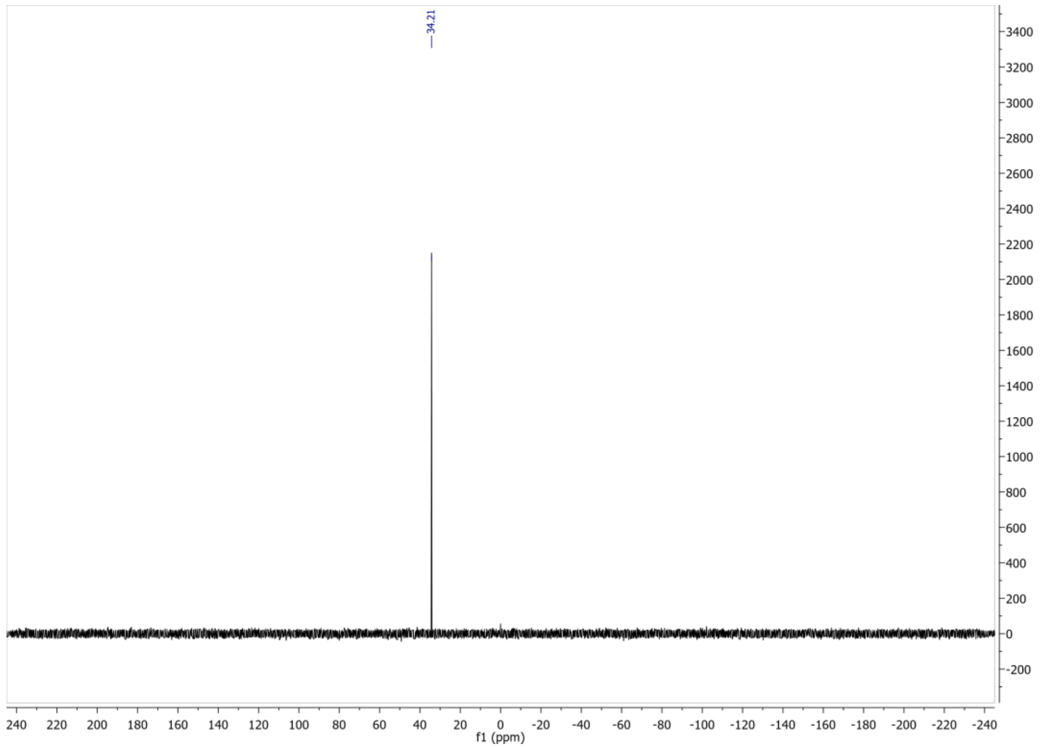

Fig. S62. 31P{1H}-NMR spectrum of[9-(S)PPh$_2$($C_{14}H_9$)] (**5**) in $CDCl_3$.

[9-(S)PPh$_2$-10-Me-($C_{14}H_8$)] (**6**)

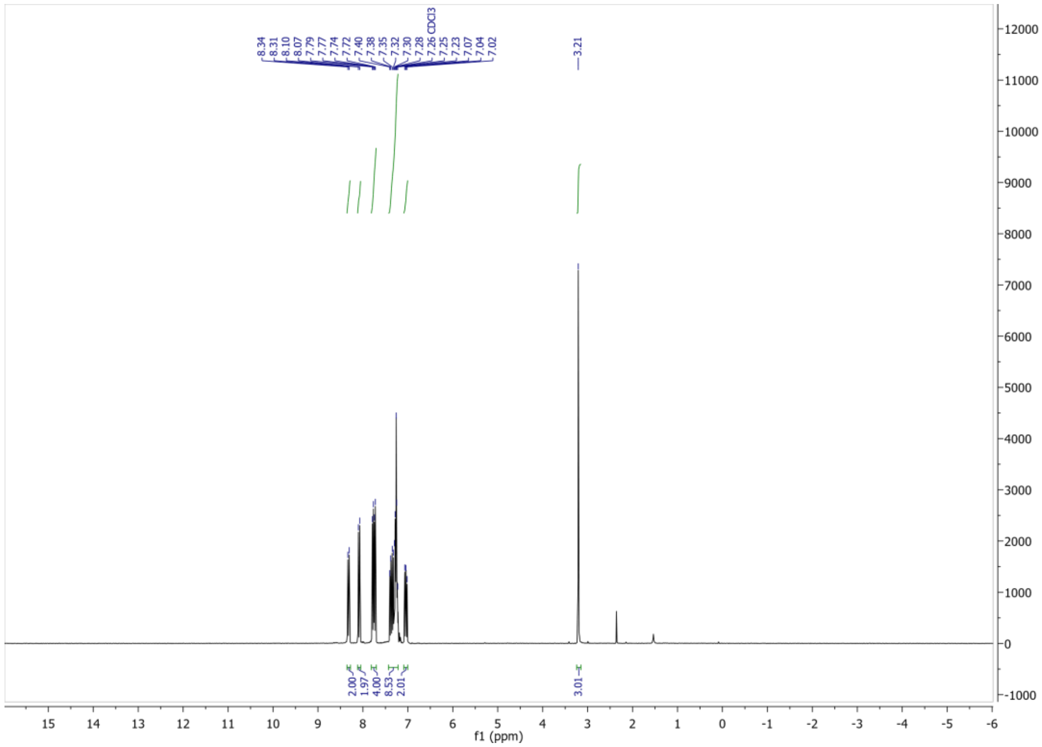

Fig. S63. 1H-NMR spectrum of [9-(S)PPh$_2$ 10-Me($C_{14}H_8$)] (**6**) in $CDCl_3$.

.

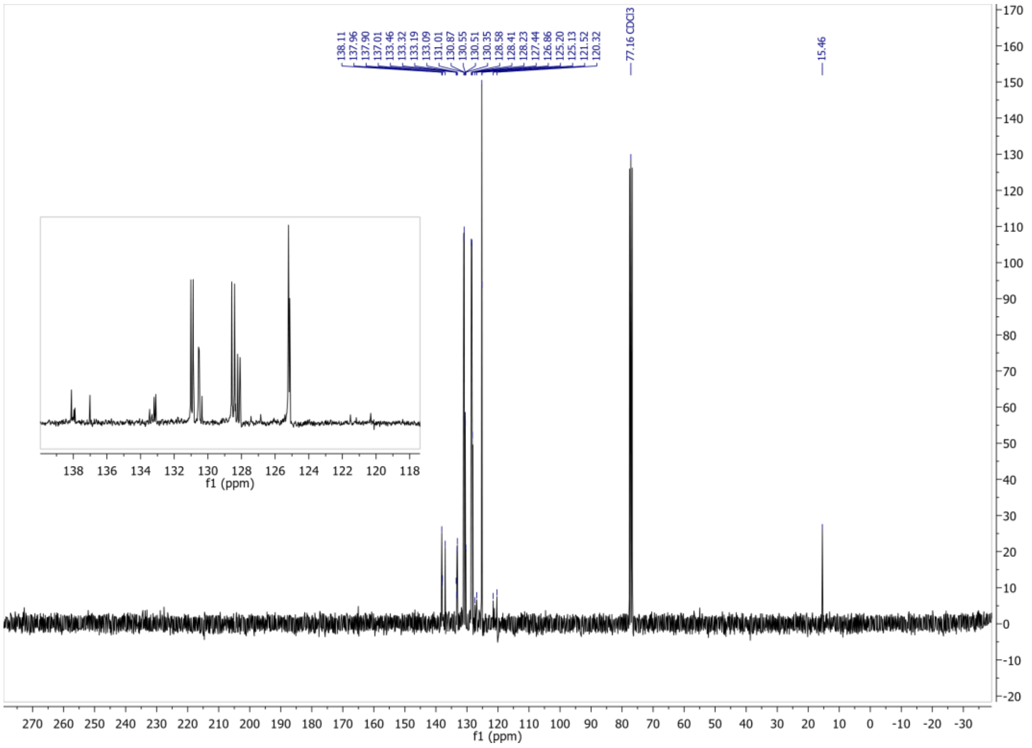

Fig. S64. 13C{1H}-NMR spectrum of [9-(S)PPh$_2$-10-Me-($C_{14}H_8$)] (**6**) in $CDCl_3$.

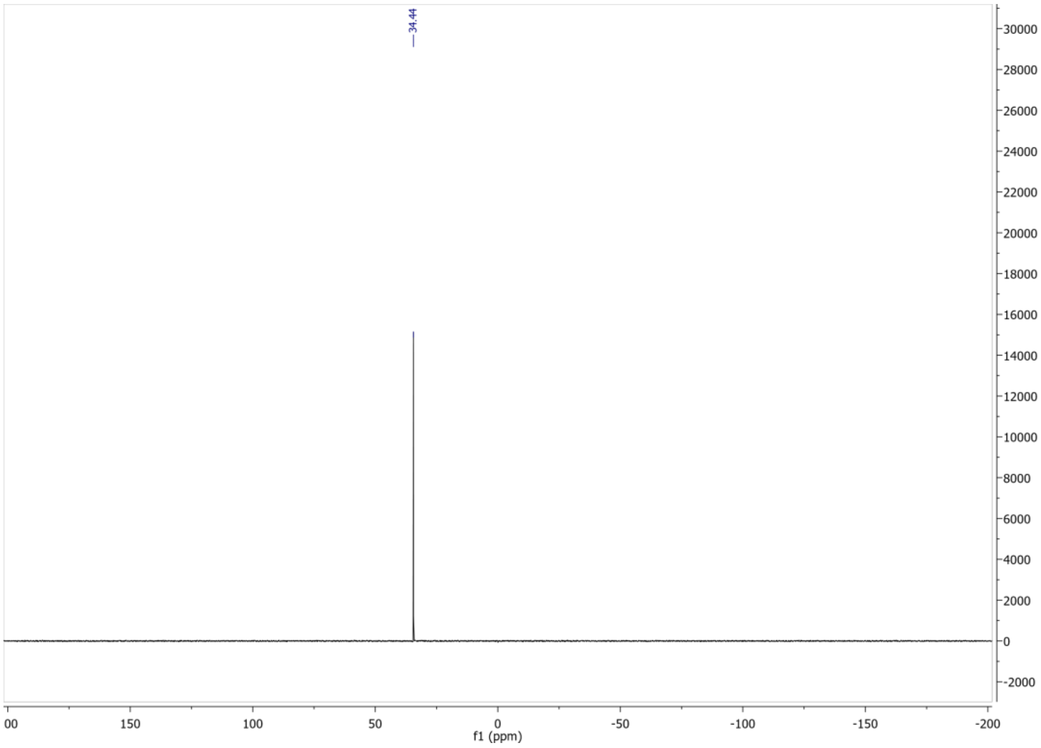

Fig. S65. 31P{1H}-NMR spectrum of [9-(S)PPh$_2$-10-Me-($C_{14}H_8$)] (**6**) in $CDCl_3$.

[9-(S)PPh$_2$-10-Et-($C_{14}H_8$] (**7**)

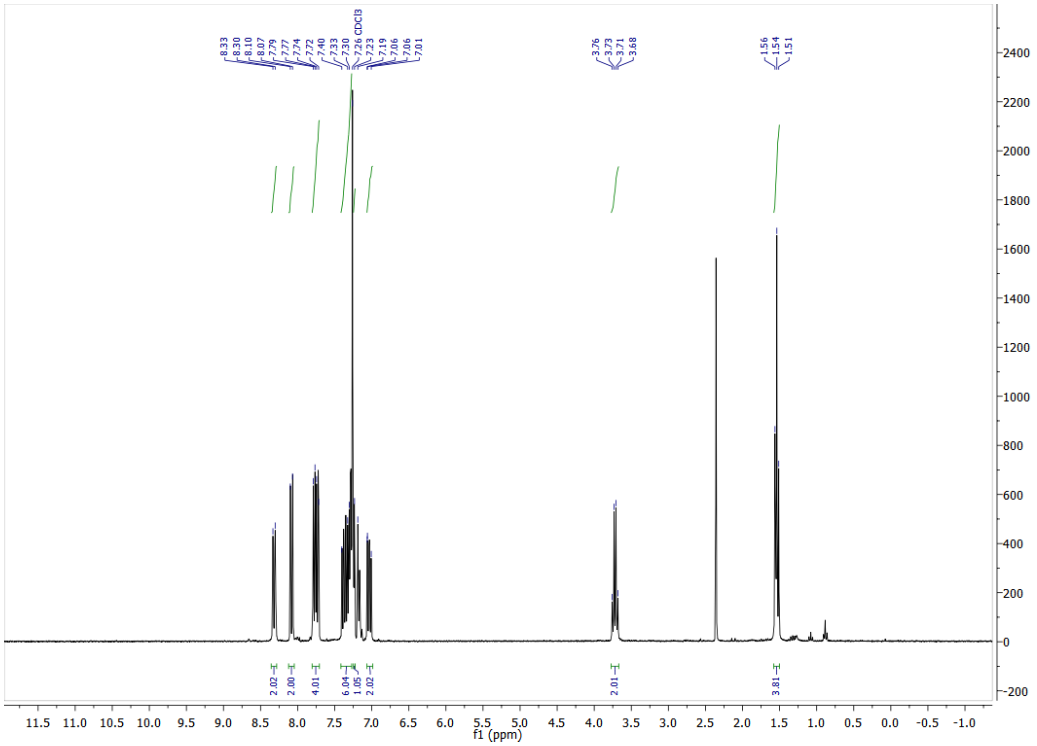

Fig. S66. 1H-NMR spectrum of 9-(S)PPh$_2$-10-Et-($C_{14}H_8$] (**7**) in $CDCl_3$. The compound contains co-crystallised toluene which could not be removed completely upon drying.

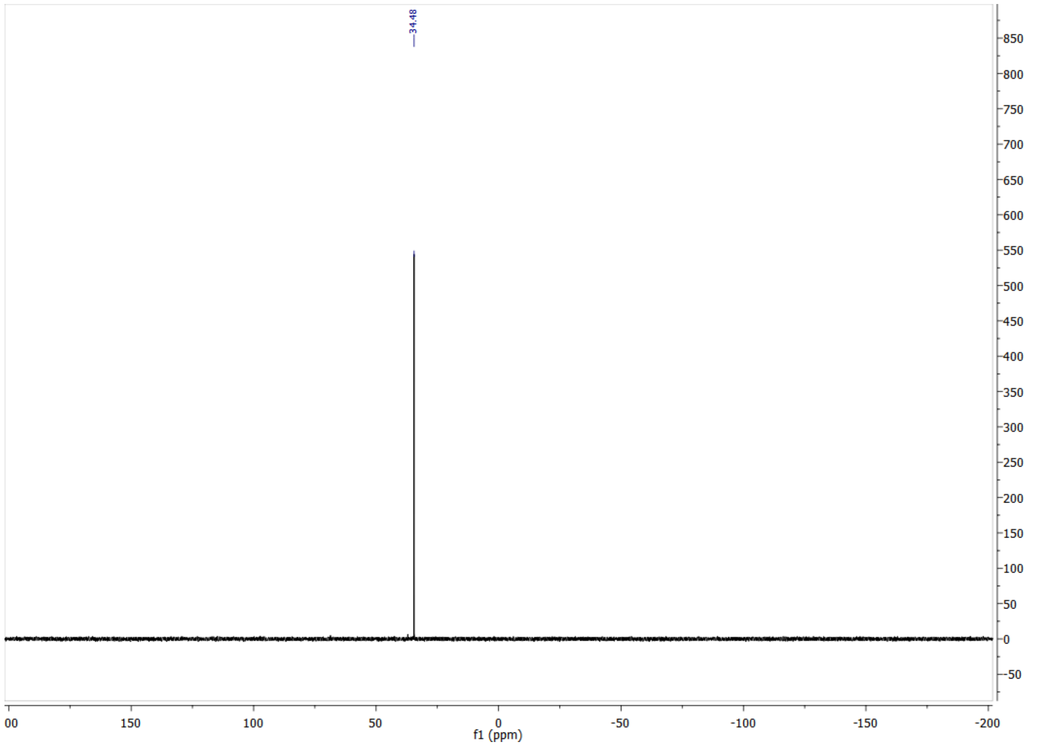

Fig. S67. 31P{1H}-NMR spectrum of [9-(S)PPh$_2$-10-Et-($C_{14}H_8$)] (**7**) in $CDCl_3$.

[9-(S)PPh$_2$-10-Ph-($C_{14}H_8$)] (**8**)

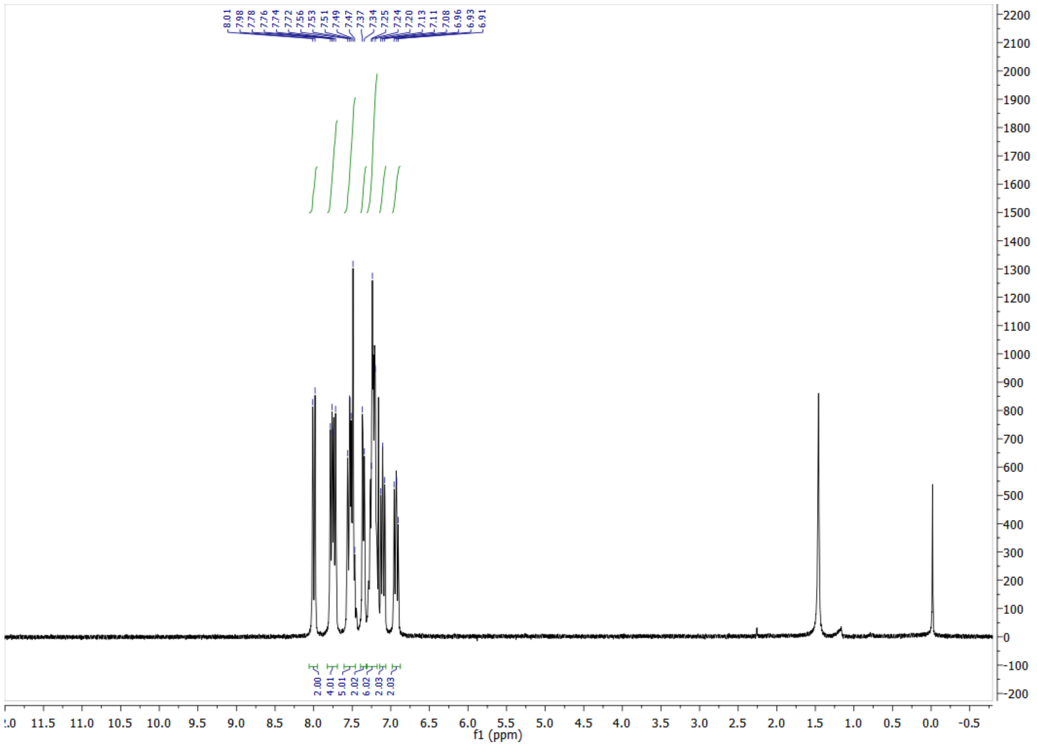

Fig. S68: 1H-NMR spectrum of spectrum of [9-(S)PPh$_2$ 10-Ph($C_{14}H_8$)] (**8**) in C_6D_6.

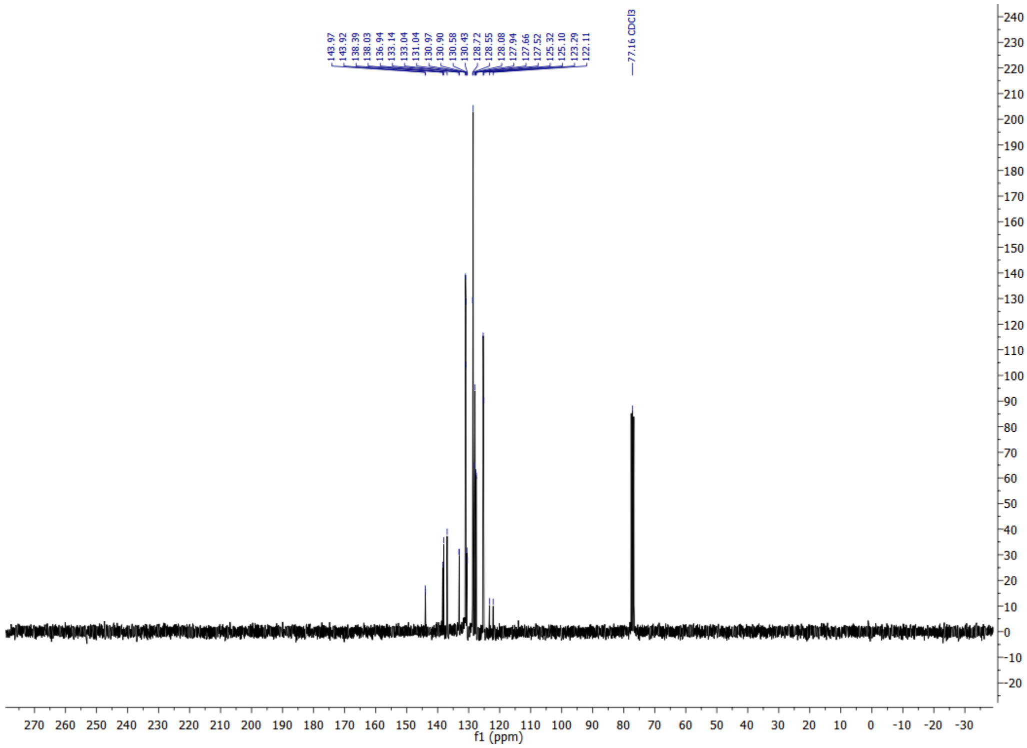

Fig. S69. 13C{1H}-NMR spectrum of [9-(S)PPh$_2$-10-Ph-($C_{14}H_8$)] (**8**) in $CDCl_3$.

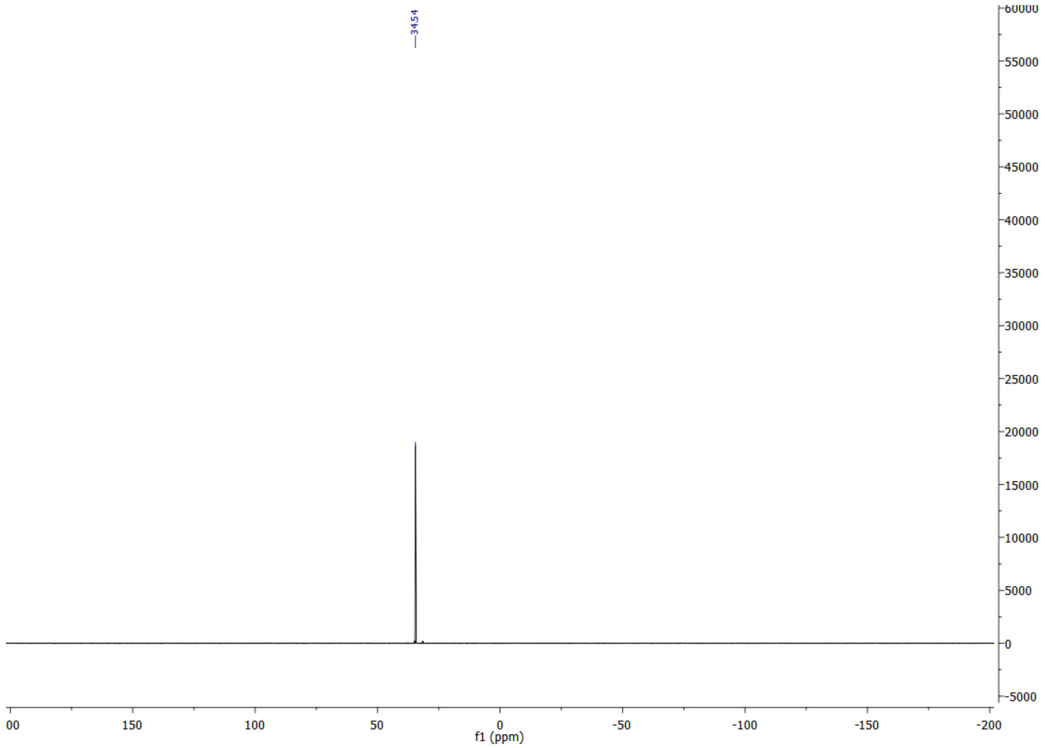

Fig. S70. 31P{1H}-NMR spectrum of [9-(S)PPh$_2$-10-Ph-($C_{14}H_8$)] (**8**) in $CDCl_3$.

S6. References

[1] G. Baccolini, C. Boga, M. Mazzacurati, *J. Org. Chem.* **2005**, *70*, 4774.

[2] M. Hayashi, T. Matsuura, I. Tanaka, H. Ohta, Y. Watanabe, *Org. Lett.* **2013**, *15*, 628.

[3] A. T. Breshears, A. C. Behrle, C. L. Barnes, C. H. Laber, G. A. Baker, J. R. Walensky, *Polyhedron* **2015**, *100*, 333.

[4] A. Thorn, B. Dittrich, G. M. Sheldrick, Acta Crystallogr. **2012**, *A68*, 448.
